# Disorganization of language and working memory systems in frontal versus temporal lobe epilepsy

**DOI:** 10.1093/brain/awac150

**Published:** 2022-05-02

**Authors:** Lorenzo Caciagli, Casey Paquola, Xiaosong He, Christian Vollmar, Maria Centeno, Britta Wandschneider, Urs Braun, Karin Trimmel, Sjoerd B Vos, Meneka K Sidhu, Pamela J Thompson, Sallie Baxendale, Gavin P Winston, John S Duncan, Dani S Bassett, Matthias J Koepp, Boris C Bernhardt

**Affiliations:** Department of Bioengineering, University of Pennsylvania, Philadelphia, Pennsylvania 19104, USA; Department of Clinical and Experimental Epilepsy, UCL Queen Square Institute of Neurology, London WC1N 3BG, UK; MRI Unit, Epilepsy Society, Chalfont St Peter, Buckinghamshire SL9 0RJ, UK; Multimodal Imaging and Connectome Analysis Laboratory, McConnell Brain Imaging Centre, Montreal Neurological Institute, Montreal, Quebec H3A 2B4, Canada; Department of Bioengineering, University of Pennsylvania, Philadelphia, Pennsylvania 19104, USA; Department of Clinical and Experimental Epilepsy, UCL Queen Square Institute of Neurology, London WC1N 3BG, UK; MRI Unit, Epilepsy Society, Chalfont St Peter, Buckinghamshire SL9 0RJ, UK; Department of Neurology, Ludwig-Maximilians-Universität, 81377 Munich, Germany; Department of Clinical and Experimental Epilepsy, UCL Queen Square Institute of Neurology, London WC1N 3BG, UK; MRI Unit, Epilepsy Society, Chalfont St Peter, Buckinghamshire SL9 0RJ, UK; Epilepsy Unit, Hospital Clínic de Barcelona, IDIBAPS, 08036 Barcelona, Spain; Department of Clinical and Experimental Epilepsy, UCL Queen Square Institute of Neurology, London WC1N 3BG, UK; MRI Unit, Epilepsy Society, Chalfont St Peter, Buckinghamshire SL9 0RJ, UK; Department of Bioengineering, University of Pennsylvania, Philadelphia, Pennsylvania 19104, USA; Department of Psychiatry and Psychotherapy, Central Institute of Mental Health, Medical Faculty Mannheim, University of Heidelberg, Mannheim, Germany; Department of Clinical and Experimental Epilepsy, UCL Queen Square Institute of Neurology, London WC1N 3BG, UK; MRI Unit, Epilepsy Society, Chalfont St Peter, Buckinghamshire SL9 0RJ, UK; Department of Neurology, Medical University of Vienna, Vienna, Austria; Department of Clinical and Experimental Epilepsy, UCL Queen Square Institute of Neurology, London WC1N 3BG, UK; MRI Unit, Epilepsy Society, Chalfont St Peter, Buckinghamshire SL9 0RJ, UK; Centre for Medical Image Computing, University College London, London, UK; Neuroradiological Academic Unit, UCL Queen Square Institute of Neurology, University College London, London, UK; Department of Clinical and Experimental Epilepsy, UCL Queen Square Institute of Neurology, London WC1N 3BG, UK; MRI Unit, Epilepsy Society, Chalfont St Peter, Buckinghamshire SL9 0RJ, UK; Department of Clinical and Experimental Epilepsy, UCL Queen Square Institute of Neurology, London WC1N 3BG, UK; MRI Unit, Epilepsy Society, Chalfont St Peter, Buckinghamshire SL9 0RJ, UK; Department of Clinical and Experimental Epilepsy, UCL Queen Square Institute of Neurology, London WC1N 3BG, UK; MRI Unit, Epilepsy Society, Chalfont St Peter, Buckinghamshire SL9 0RJ, UK; Department of Clinical and Experimental Epilepsy, UCL Queen Square Institute of Neurology, London WC1N 3BG, UK; MRI Unit, Epilepsy Society, Chalfont St Peter, Buckinghamshire SL9 0RJ, UK; Department of Medicine, Division of Neurology, Queen’s University, Kingston, Ontario, Canada; Department of Clinical and Experimental Epilepsy, UCL Queen Square Institute of Neurology, London WC1N 3BG, UK; MRI Unit, Epilepsy Society, Chalfont St Peter, Buckinghamshire SL9 0RJ, UK; Department of Bioengineering, University of Pennsylvania, Philadelphia, Pennsylvania 19104, USA; Department of Physics and Astronomy, University of Pennsylvania, Philadelphia, PA 19104, USA; Department of Electrical and Systems Engineering, University of Pennsylvania, Philadelphia, PA 19104, USA; Department of Neurology, University of Pennsylvania, Philadelphia, PA 19104, USA; Department of Psychiatry, University of Pennsylvania, Philadelphia, PA 19104, USA; Santa Fe Institute, Santa Fe, NM 87501, USA; Department of Clinical and Experimental Epilepsy, UCL Queen Square Institute of Neurology, London WC1N 3BG, UK; MRI Unit, Epilepsy Society, Chalfont St Peter, Buckinghamshire SL9 0RJ, UK; Multimodal Imaging and Connectome Analysis Laboratory, McConnell Brain Imaging Centre, Montreal Neurological Institute, Montreal, Quebec H3A 2B4, Canada

**Keywords:** frontal lobe epilepsy, temporal lobe epilepsy, cognition, fMRI, multiscale functional mapping

## Abstract

Cognitive impairment is a common comorbidity of epilepsy and adversely impacts people with both frontal lobe (FLE) and temporal lobe (TLE) epilepsy. While its neural substrates have been investigated extensively in TLE, functional imaging studies in FLE are scarce. In this study, we profiled the neural processes underlying cognitive impairment in FLE and directly compared FLE and TLE to establish commonalities and differences. We investigated 172 adult participants (56 with FLE, 64 with TLE and 52 controls) using neuropsychological tests and four functional MRI tasks probing expressive language (verbal fluency, verb generation) and working memory (verbal and visuo-spatial). Patient groups were comparable in disease duration and anti-seizure medication load. We devised a multiscale approach to map brain activation and deactivation during cognition and track reorganization in FLE and TLE. Voxel-based analyses were complemented with profiling of task effects across established motifs of functional brain organization: (i) canonical resting-state functional systems; and (ii) the principal functional connectivity gradient, which encodes a continuous transition of regional connectivity profiles, anchoring lower-level sensory and transmodal brain areas at the opposite ends of a spectrum. We show that cognitive impairment in FLE is associated with reduced activation across attentional and executive systems, as well as reduced deactivation of the default mode system, indicative of a large-scale disorganization of task-related recruitment. The imaging signatures of dysfunction in FLE are broadly similar to those in TLE, but some patterns are syndrome-specific: altered default-mode deactivation is more prominent in FLE, while impaired recruitment of posterior language areas during a task with semantic demands is more marked in TLE. Functional abnormalities in FLE and TLE appear overall modulated by disease load. On balance, our study elucidates neural processes underlying language and working memory impairment in FLE, identifies shared and syndrome-specific alterations in the two most common focal epilepsies and sheds light on system behaviour that may be amenable to future remediation strategies.

## Teaser

Studying frontal lobe epilepsy with fMRI and network science, Caciagli *et al.* identify impaired activation of attentional and executive areas and impaired default-mode deactivation as key neural processes mediating cognitive impairment and capture distinct functional signatures in frontal versus temporal lobe epilepsy.

## Introduction

Frontal lobe epilepsy (FLE), the second most common focal epilepsy syndrome after temporal lobe epilepsy (TLE), is frequently drug-resistant and MRI-negative.^1–3^ Cognitive impairment is common in both FLE and TLE and adversely impacts quality of life and psychosocial functioning.^4^ Impaired episodic memory and semantic knowledge are common in TLE, although dysexecutive traits frequently coexist.^5,6^ In contrast, FLE has a less established cognitive signature, with multiple cognitive domains being affected, including dexterity, attention, working memory, verbal fluency, executive functions and episodic memory.^7–11^ Whether cognitive profiles in FLE and TLE may be distinct remains controversial, and several investigations concluded that these syndromes cannot be discriminated based on cognitive measures.^8,12,13^ It is however suggested that episodic memory impairment is more profound in TLE, while executive functions may be more affected in FLE.^7,14–16^

Task-based functional MRI (fMRI) probes the neural correlates of cognitive impairment in epilepsy. In TLE, altered parietal and mesiotemporal activation and connectivity underlie working memory impairment,^17,18^ while language fMRI studies indicate altered fronto-temporal functional profiles, with complex intra- and inter-hemispheric reorganization.^19–25^ In contrast, few studies investigated FLE.^26^ Children with FLE had reduced fronto-temporo-parietal connectivity during working memory fMRI but no substantial alteration in regional activation.^27^ In adults with FLE, we previously reported enhanced fronto-temporal activation during episodic memory encoding and reduced mesiotemporal activation in those with poorer memory.^28^ Abnormal motor and parietal activity may underlie impaired dexterity.^29^ Overall, a comprehensive overview of the neural substrates of cognitive impairment in FLE is lacking.

Here, we aimed to characterize the functional neuroanatomy of expressive language and working memory, cognitive functions reliant on frontal lobe processing,^30,31^ in individuals with drug-resistant FLE who underwent neuropsychological tests and four fMRI tasks. We compared people with FLE to (i) healthy controls, and (ii) a ‘patient control group’ of individuals with TLE, comparable in epilepsy duration and anti-seizure medication (ASM) load, which allowed us to establish shared and syndrome-specific traits. We devised a multiscale functional mapping framework to investigate the landscape of brain activation and deactivation during cognition^32–34^ and capture disease-related reorganization. Pursuant to an ensemble view on the reconfiguration of task-related brain activity, we complemented traditional voxel-based fMRI maps, which elucidate task-related signatures at a regional level, by profiling task effects across two motifs of brain organization: (i) established resting-state functional systems^35^; and (ii) the principal functional connectivity gradient.^36,37^ The gradient, in particular, describes a continuous transition of neural function that anchors unimodal sensory areas and high-order transmodal regions at two opposite ends of a spectrum, providing an axis of subregional cortical organization and recapitulating established models of cortical hierarchy.^38^ Thus, the gradient offers a compact, yet formal, framework to characterize organizational aspects of cognitive activity, which allows us to (i) describe task-fMRI signatures in the context of a global balance of sensorimotor and high-order, perceptually-decoupled processing, as exemplified by work in healthy adults^39–41^ and people with TLE performing a pattern separation task^42^; and (ii) derive global metrics that quantify group differences in task-related systems-level reorganization. By conveying regional, systems-level and global viewpoints on the neural signatures of cognitive impairment in epilepsy, our approach collectively proves sensitive to both localized and higher-order abnormalities.

We anticipated expressive language and working memory impairment in FLE. We hypothesized that such impairment would be underpinned by (i) reduced activation of areas engaged during task execution, i.e. ‘task-positive’ regions; (ii) reduced deactivation of default-mode areas (DMN), i.e. ‘task-negative’ regions; and (iii) global disorganization of cognitive system recruitment, as quantified via gradient analyses. We also hypothesized that, based on the proximity to the epileptic focus, (i) frontal and systems-level working memory abnormalities may be more prominent in FLE than TLE; (ii) language-related activation of frontal areas would be lower in FLE; and (iii) engagement of temporal language areas would be lower in TLE. We also aimed to corroborate the neurobehavioural validity of our fMRI tasks by correlating imaging patterns with neuropsychological and task performance measures. Finally, we explored associations between cognitive network alterations and clinical characteristics, probed the potential effects of frontal lobe lesions, and replicated our main FLE findings in a more homogeneous patient subgroup with frontal cortical dysplasia.

## Materials and methods

### Participants

This study investigated 172 participants recruited from 2007 to 2013: 120 drug-resistant patients under surgical consideration, 56 with FLE (29 female, 30/26 left-/right-sided FLE), 64 with TLE (44 female, 34/30 left-/right-sided TLE) and 52 healthy controls (30 female) without neurological or psychiatric diagnoses and no family history of epilepsy. Demographic and clinical details are provided in Table 1.

**Table 1:** Demographic and clinical characteristics

	CTR (*n* = 52)	FLE (*n* = 56)	TLE (*n* = 64)	Test statistic (*F*, *H or* χ²)	*P*-value	*Post hoc* tests (Bonferroni-corrected)
Age at scan, years, mean (SD)	34.1 (10.4)	33.4 (10.2)	39.2 (10.7)	5.6	**0**.**005**	FLE/CTR: 1.00
						FLE/TLE: **0.008**
						TLE/CTR: 0.029
Sex, female/male	30/22	29/27	44/20	3.7	0.15	
Handedness, L/R/A	6/46/0	5/49/2	10/52/2	3.0	0.59	
Side of seizure focus, L/R	–	30/26	34/30	0.0	1.00	
Aetiological categories, non-lesional/FCD/DNET/glial tumour/other^a^/HS	–	29/13/6/3/5/0	0/0^b^/0^b^/0/0/64	–	–	
Frontal lesion site, (pre)motor-SMA/lateral/mesial/orbital/other	–	12/9/1/3/2	–	–	–	
Age of epilepsy onset, years, median (IQR)	–	10.0 (6.0)	13.0 (11.8)	2.3	0.13	
Duration of epilepsy, years, median (IQR)	–	21.0 (15.0)	22.5 (24.3)	0.6	0.44	
Seizure frequency, monthly, log, mean (SD)	–	1.15 (0.81)	0.87 (0.55)	4.8	**0**.**03**	
FBTCS, yes/no	–	31/23	20/44	8.2	**0**.**005**	
FBTCS frequency, monthly, log, mean (SD)	–	−0.07 (0.86)	−0.32 (0.62)	1.2	0.27	
Time since last seizure, days, median (IQR)	–	1.0 (6.8)	5.0 (8.0)	2.8	**0**.**005**	
AEDs, median (IQR)	–	3.0 (1.0)	2.5 (1.0)	1.3	0.25	
Topiramate/Zonisamide, yes/no	–	15/41	12/52	1.1	0.38	
Levetiracetam, yes/no	–	29/27	41/23	1.9	0.20	
Task-fMRI data availability, verbal fluency/verb generation/verbal WM/visual WM, no. of participants	52/51/51/52	56/56/53/50	63/63/63/62	–	–	
Frontal language LI, verbal fluency fMRI, median (IQR)	0.79 (0.32)	0.67 (0.56)	0.76 (0.29)	10.5	**0**.**005**	FLE/CTR: **0.004**
						FLE/TLE: 0.094
						TLE/CTR: 0.744
Frontal language LI, verb generation fMRI, median (IQR)	0.81 (0.23)	0.65 (1.15)	0.70 (0.41)	9.1	**0**.**01**	FLE/CTR: **0.019**
						FLE/TLE: 1.00
						TLE/CTR: **0.030**

A = ambidextrous; CTR = healthy controls; HS = hippocampal sclerosis; IQR = interquartile range; L = left; LI = laterality index; R = right; WM = working memory. In the *P-*value and *Post hoc* tests columns, statistically significant group comparisons (*P* < 0.05) are highlighted in bold font. Categorical variables were compared with Fisher’s exact test (χ² statistic is reported). Continuous normally/non-normally distributed demographic variables are reported as mean (SD)/median (IQR) and were compared via ANOVA (*F* statistic)/Kruskal–Wallis test (*H* statistic), respectively. Pairwise deletion was applied in case of missing data. Age at onset (and, consequently, epilepsy duration) could not be accurately documented for five people with FLE. For details about computation of language LIs, see Supplementary material.

One patient with possible periventricular nodular heterotopia, and five with miscellaneous MRI abnormalities (described in the ‘Materials and methods’ section).

Hippocampal sclerosis coexisted with DNET in three patients (one left/two right) and with a possible FCD in one patient (right).

**Figure 1:**
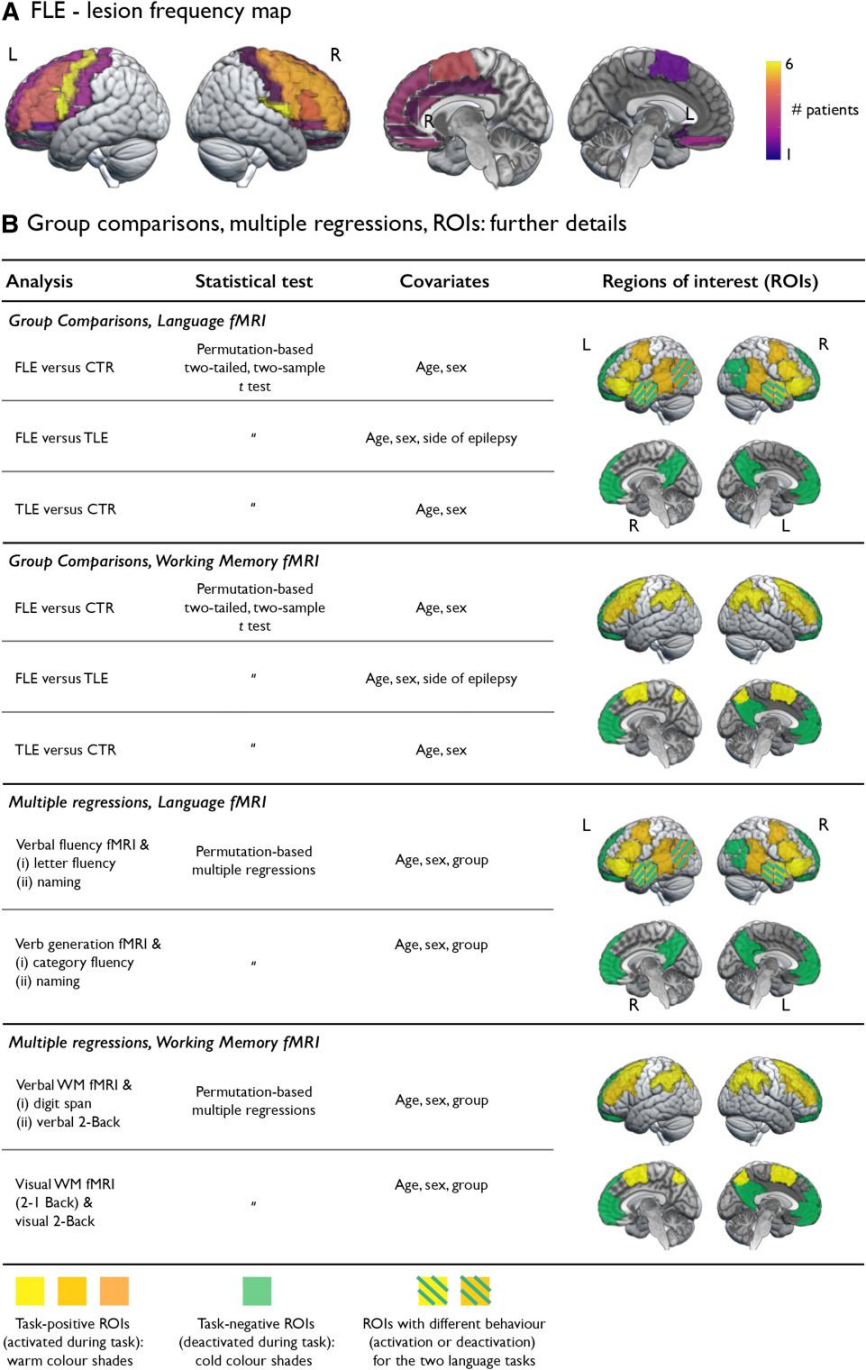
**Lesion frequency map and statistical details for voxel-based analyses.** (**A**) Lesion frequency map based on ROIs of the Automated Anatomical Labeling atlas, version 3,^43^ obtained via (i) computing how many individuals with FLE would present with a lesion that overlapped with a given ROI; and (ii) depicting such information with a colour scale that transitions from dark colours (low number) to light colours (higher number). In a patient subset, frontal lobe lesions also marginally encroached upon the post-central gyrus or the insula, leading to such areas that border frontal lobe boundaries being associated with a non-zero number. (**B**) Details about voxel-based statistical models probing group comparisons and associations between fMRI task effects and cognitive performance. Language and working memory ROIs showing task-related activation (‘task-positive’) and deactivation (‘task-negative’, corresponding to DMN areas) are shown in warm and cold colour shades, respectively. Task signal in some temporo-parietal ROIs exhibits different task-related patterns (activation or deactivation) during the two language tasks; such areas are depicted with warm colours and superimposed cold-coloured dashes. Group differences encompassing the latter regions are interpreted in terms of activation or deactivation differences (e.g. higher activation or lesser deactivation in FLE than controls) depending on the task-related behaviour of a given ROI (activated or deactivated) in each task, as identified via one-sample *t-*tests in controls.

Diagnosis of FLE was determined by expert epileptologists based on history, seizure semiology, video-EEG telemetry and 3 T structural MRI; PET, ictal single-photon emission computerized tomography (SPECT) and magneto-encephalography data were available for a patient subset. In 29 patients, MRI was non-lesional (left/right: 17/12). Findings in the remainder of patients included areas of suspected focal cortical dysplasia (FCD, *n* = 13; left/right: 6/7; pathologically confirmed in 8 of 8 patients who subsequently had surgery); dysembryoplastic neuroepithelial tumour (DNET, *n* = 6; left/right: 3/3); low-grade glial tumour (*n* = 3, all right); possible periventricular nodular heterotopia (*n* = 1, left); or unequivocal signal abnormalities, concordant with clinical and EEG findings [*n* = 4, left/right: 3/1; one post-traumatic, one of intrauterine (vascular) aetiology and two areas of cortical injury of unclear aetiology]. A lesion frequency map^43^ is shown in Fig. 1.

In people with TLE, interictal and ictal scalp video-EEG confirmed and lateralized seizure onset to the temporal lobe. All had ipsilateral hippocampal sclerosis on 3 T MRI, as determined by qualitative neuroradiological diagnosis and/or via quantitative assessments of hippocampal volumes^44^ and T_2_ relaxation times,^45^ with pathological confirmation in those who subsequently underwent surgery. Hippocampal sclerosis coexisted with ipsilateral DNET in three patients (left/right: 2/1) and a possible FCD in one patient (right).

Written informed consent was obtained from all participants according to the standards of the Declaration of Helsinki. Participant recruitment was approved by the University College London Queen Square Institute of Neurology and University College London Hospitals Research Ethics Committee. Exclusion criteria were non-proficiency in written and spoken English, MRI contraindications, pregnancy and inability to give informed consent. Individuals who experienced focal to bilateral tonic-clonic seizures (FBTCS) <24 h before the investigation were excluded or had their testing session rescheduled.

Groups were comparable for handedness and (binary) sex, but not for age, which was used as a covariate in all group analyses. Patient groups did not differ in age at seizure onset and epilepsy duration, number of ASMs and usage of levetiracetam or topiramate/zonisamide, which more favourably or unfavourably influence cognitive system activity than other common ASMs, respectively.^46,47^ Patients with FLE had more frequent seizures, shorter time since last seizure and more frequent history of FBTCS in the year before the investigation than those with TLE (Table 1). As FLE is heterogeneous in terms of aetiology and MRI findings, we separately analysed a subgroup with a more homogeneous aetiology (FCD; *n* = 13), directly compared FLE patients with and without lesions and probed the influence of clinical variables on imaging findings. Moreover, we separately investigated left and right FLE subgroups.

### Neuropsychological data

Participants underwent standardized neuropsychological tests,^48^ providing measures of general intellectual level (IQ, National Adult Reading Test^49^), working memory [digit span and Wechsler Adult Intelligence Scale (WAIS III)^50^ scores], letter and category fluency^51^ (sum of words generated for letter ‘S’, sum of items generated for the category ‘Animals’ in 1 min), naming (McKenna Graded Naming Test^52^), psychomotor speed and executive function (mental flexibility; Trail Making Test A and B-A^53^) and verbal and visuo-spatial learning and recall (List and Design Learning, A1–A5 and A6, Adult Memory and Information Processing Battery^54^). Verbal reasoning and comprehension measures (Vocabulary and Similarities, WAIS III^50^ scaled scores) were available for patient groups. Pairwise deletion was used for missing data.

### Imaging data acquisition and fMRI tasks

Imaging data were acquired on the same GE SignaHDx 3T MRI scanner at the Epilepsy Society, Chalfont St Peter, Buckinghamshire, UK. For all tasks, we used a 50-slice gradient echo-planar sequence with axial orientation, 64 × 64 matrix, in-plane voxel size 3.75 × 3.75 mm, 2.4 mm slice thickness, 0.1 mm inter-slice gap, echo time/repetition time: 25/2500 ms.^55^ One visuo-spatial and one verbal fMRI paradigm assessed working memory. During the visuo-spatial (Dot Back) task, dots appeared in four possible locations on a screen. Participants were instructed to move a joystick to the position of the currently presented dot (0 Back) or the position of the dot displayed one (1 Back) or two presentations earlier (2 Back).^55^ There were five 30 s blocks for each condition in pseudo-random order, intermixed with 15 s of cross-hair fixation. During the verbal working memory task, single concrete nouns were displayed every 3 s within 30 s blocks. Participants responded upon display of a given control word (active control condition) or upon recurrence of a word displayed two presentations earlier (2 Back working memory). There were five 30 s blocks per condition, intermixed with 15 s of cross-hair fixation. Two covert (silent)^56^ tasks probed expressive language, followed by out-of-scanner cognitive testing in the same session. During verbal fluency fMRI, participants generated words beginning with a visually-presented letter (A/D/E/S/W, one letter per block, five 30 s blocks), alternating with 30 s blocks of cross-hair fixation.^57^ During the verb generation task, subjects generated verbs associated with a visually-displayed noun (‘Generate’) or repeated a visually-displayed noun (‘Repeat’). There were four 30 s blocks per condition and four cross-hair fixation blocks.^58^

### Statistical analysis of clinical and neuropsychological data

Data were analysed using R 3.6.1 and SPSS 27. For demographics, we used Fisher’s exact test, one-way ANOVA and Kruskal–Wallis tests for categorical, continuous parametric and nonparametric variables, respectively. Neuropsychological data were compared via ANCOVA, covarying for age and sex. Comparisons against published norms were attained with one sample *t-*tests. Working memory task performance measures were not normally distributed and were compared via Kruskal–Wallis tests. Across cognitive domains, we corrected for multiple comparisons via the false discovery rate (FDR) procedure.^59^*Post hoc* tests were Bonferroni-corrected.

### Functional MRI data: pre-processing and voxel-based statistics

Functional imaging data were analysed with SPM12 (https://www.fil.ion.ucl.ac.uk/spm/). Images were realigned, normalized to a scanner- and acquisition-specific echo-planar imaging template in Montreal Neurological Institute (MNI) space, resampled to 3 × 3 × 3 mm isotropic voxels and smoothed with a Gaussian kernel of 8 × 8 × 8 mm full-width at half-maximum.^60^ Individual-level condition-specific effects were derived via general linear models. Task conditions were modelled as 30 s blocks and convolved with the canonical haemodynamic response function. For verbal fluency fMRI, we created activation contrasts associated with generating words. For verb generation fMRI, we subtracted word repetition from word generation. For verbal working memory fMRI, we subtracted verbal monitoring from the 2 Back working memory condition. For visuo-spatial working memory fMRI, we contrasted the condition with low working memory demand against the active control condition (1–0 Back) and directly compared activation for high and low working memory demand (2–1 Back). Voxel-wise contrast estimates (β weights) were computed with six motion parameters as confound regressors. Scans with a mean framewise displacement > 0.5 mm were discarded from further analysis.^61^ Further quality checks are detailed in the Supplementary material.

Group analyses were conducted with nonparametric permutation tests using SnPM13^62^ (http://www.nisox.org/Software/SnPM13/) to attain methodological homogeneity across analytical scales. One-sample permutation *t-*tests assessed effects of each task condition per group. Following exploratory permutation-based *F-*tests, group differences were assessed via two-sample permutation *t*-tests, all with 10 000 permutations and age and sex as covariates. Comparisons of FLE and TLE included side of epilepsy as an additional covariate (Fig. 1). Statistical significance was set at two-tailed *P* < 0.05, voxel-wise corrected for family-wise error rate (FWE)^63^ within pre-specified language, working memory and default-mode (‘task-negative’) regions of interest (ROIs; Fig. 1 and Supplementary material). For completeness, we report group differences for areas outside such ROIs at two-tailed *P*_FWE_ < 0.05, voxel-wise corrected brain-wide.

### Functional MRI data: canonical systems and principal gradient

**Figure 2:**
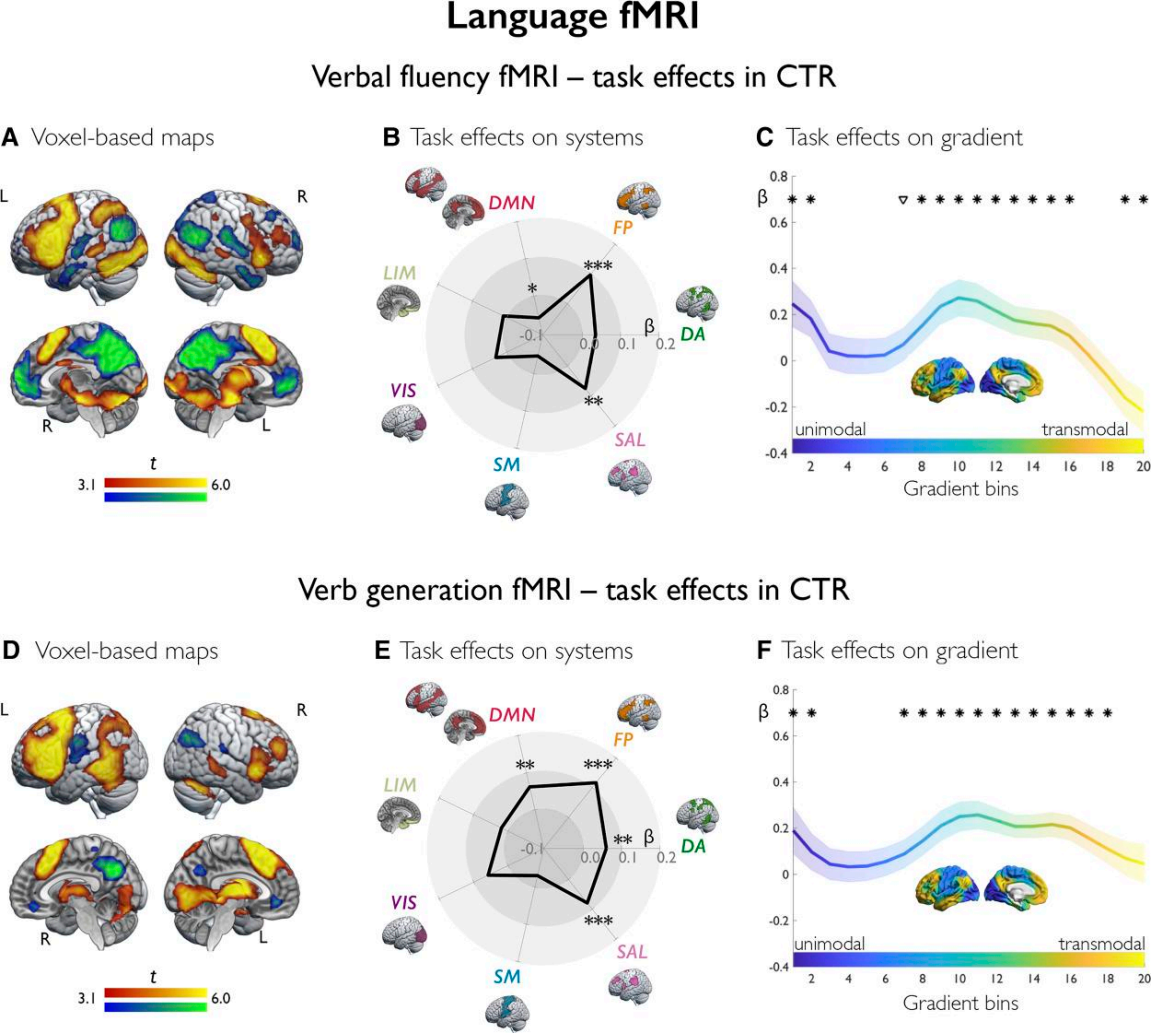
**Language fMRI: task effects in controls.** Task effects in controls (CTR) for verbal fluency (**A**–**C**) and verb generation fMRI (**D**–**F**), respectively. **A** and **D** show voxel-based activation (warm colours) and deactivation (cold colours) in CTR, as derived from one-sample *t*-tests. Brain renders show maps at *P* < 0.001 uncorrected, with an extent threshold of 10 voxels applied for display purposes; colour bars indicate *t*-score scales. The spider plots (**B** and **E**) show mean task-related effects, parameterized as contrast estimates (β weights) across seven canonical systems, where DA = dorsal attention; FP = frontoparietal control; LIM = (para)limbic; SAL = salience (ventral attention); SM = somatomotor; VIS = visual. ***P_FDR_ < 0.01, **P_FDR_ < 0.05, *uncorrected *P* < 0.05. **C** and **F** show task-related effects (β weights) stratified along the principal gradient, which depicts a continuous coordinate system running from unimodal areas (dark blue) at one end to transmodal (yellow) areas at the other end; its left lateral and midline views are shown in the middle of each plot with the same colour scale as in curves of gradient-stratified task effects. The gradient was discretized into 20 consecutive, equally-sized bins (*x*-axis), with task-related signal (β weights; *y*-axis) computed per bin; shaded areas refer to 95% confidence intervals of mean effects at each bin; *P_FDR_ < 0.05, ^∇^uncorrected *P* < 0.05.

We quantified task effects across seven resting-state functional systems^35^ (Fig. 2): visual, somatomotor, dorsal attention, salience (or ventral attention), (para)limbic, frontoparietal control and DMN. For each task contrast, we extracted β weights from all parcels of the Schaefer brain atlas^64^ (200 ROI scale, MNI space) using FSL-6.0.2, averaged β weights across ROIs belonging to a given system and adjusted them for age and sex via multiple regression. We profiled task effects along the principal functional connectivity gradient in surface space^36,65^ (Supplementary material). The gradient was computed from resting-state fMRI data of 100 Human Connectome Project (HCP) participants via non-linear dimensionality reduction of surface-registered functional connectivity metrics.^66,67^ HCP acquisition and preprocessing have been detailed elsewhere.^68^ The gradient was discretized into 20 equally-sized bins, as previously reported^39,65^; cortical locations were assigned to each bin, with sensory/motor regions assigned to the 1st bin and transmodal regions assigned to the 20th bin. For each participant and task contrast, we derived average β weights per bin via a sliding window approach^69^ and adjusted them for age and sex via multiple regression.

In controls, one-sample permutation *t*-tests assessed task effects per system or gradient bin. We computed deviation (*Z*) scores to determine the atypicality of effects in patients [Z*_pat_* = (Act*_pat_*−μ*_CTR_*)/σ_CTR_], where μ*_CTR_* and σ*_CTR_* correspond to the mean and standard deviation of a systems-level or bin-wise β weight in controls for a given task contrast.^70,71^ For each system or gradient bin, *Z*-score deviations from zero in patients were assessed with two-tailed, permutation-based one-sample *t-*tests. FLE and TLE were compared via permutation-based two-tailed two-sample *t-*tests. We also assessed global differences between curves of gradient-stratified task effects (area between curves, AbC), using a nonparametric permutation test based on functional data analysis (FDA) techniques^72^ (Supplementary material). We used 10 000 permutations for all tests and report Cohen’s *d* effect sizes. *P*-values were FDR-adjusted for number of systems or gradient bins; comparisons reaching uncorrected *P* < 0.05 (*P*_unc_) are reported for completeness. Sensitivity analyses probed effects across DMN and frontoparietal control system subdivisions derived from a more fine-grained 17-system parcellation.^35^

### Correlation of fMRI data with cognitive and clinical variables

Across scales, we assessed correlations of task effects with cognitive performance in all participants^21,42^ using permutation-based analyses entailing 10 000 permutations. Voxel-based regressions were conducted with SnPM13; age, sex and group were nuisance covariates. Associations between cognitive scores and fMRI metrics were explored within language, working memory and task-negative ROIs (Fig. 1). Effects are reported at two-tailed, voxel-wise *P*_FWE_ < 0.05. For correlations between cognitive scores and task effects across systems or on the gradient, parameterized as age- and sex-adjusted β weights, we employed permutation-based two-tailed product-moment correlations. For working memory task performance measures, which were skewed, we employed permuted rank correlations. Correlations between fMRI activity and clinical variables, such as age at seizure onset, disease duration, seizure frequency, FBTCS history and time since last seizure^21,73,74^ were separately computed in FLE and TLE to disentangle syndrome-specific effects using SnPM13-based regressions with sex and side of seizure focus as covariates; age was an additional covariate for models including seizure frequency, FBTCS and time since last seizure. Statistical significance was established using the same ROIs as above. For correlations between clinical variables and task effects across systems or gradient, we used two-tailed, permutation-based correlations.

### Data availability

Data to reproduce the main group findings are available on NeuroVault (https://identifiers.org/neurovault.collection:13042). Other data are not publicly available due to their containing information that could compromise the privacy of research participants. Example code is available at: https://github.com/lcaciagl/Language_WM_FLE_vs_TLE.

## Results

### Neuropsychological data and fMRI task performance

Patients with FLE differed from controls and/or published norms for most cognitive measures (all *P*_FDR_ < 0.001; see Supplementary Table 1 for test scores and associated statistics). Patients with FLE had better performance on naming, verbal learning and verbal recall tests and worse performance on a mental flexibility test than those with TLE (*post hoc P* < 0.05, Bonferroni-corrected). Working memory and verbal fluency were equally impaired in FLE and TLE. Verbal working memory task execution was less accurate in FLE than controls, but similar between FLE and TLE (>80% median accuracy in both patient groups). For visual working memory, performance in FLE was worse than controls, with more marked differences for higher task difficulty; there were no differences between FLE and TLE. Supplementary Table 1 provides details regarding fMRI task performance scores and associated statistics.

### Cognitive fMRI: synopsis

During language tasks, we found reduced frontal activation and reduced deactivation of DMN nodes in FLE compared to controls. During working memory, FLE showed reduced frontoparietal activation, reduced DMN deactivation and global disorganization of task-related recruitment. For visual working memory, we observed a combination of (i) increased frontoparietal activation and less DMN deactivation than controls for low-level task demands, followed by (ii) reduced frontoparietal activation for higher task demands. Patterns of dysfunction in FLE and TLE broadly overlapped; altered DMN deactivation, however, was more evident in FLE, while reduced activation of posterior language areas was more marked in TLE.

The following sections detail these findings. For voxel-based analyses, the figures show regionally unconstrained whole-brain maps and outline corrected as well as uncorrected findings for completeness, in accord with benchmark evidence.^75^ As detailed above, voxel-based statistical tests focused on effects in prespecified cortical regions, and we only discuss findings surviving voxel-wise FWE-correction for multiple comparisons. Statistical details are provided in Supplementary Tables 2–17.

### Verbal fluency fMRI

In controls, the verbal fluency task activated fronto-temporo-parietal cortices, hippocampus and subcortical regions (Fig. 2); deactivation encompassed DMN areas, including medial prefrontal, medial parietal and angular cortices. Analysis of systems provided an ensemble perspective on these findings, showing activation of frontoparietal control and salience systems (β = 0.10/0.08, *P*_FDR_ = 0.004/0.020), and tendencies for deactivation of the whole DMN (β = −0.06, *P*_unc_ = 0.038). Gradient-based profiling sorted cortical regions according to a sensory-to-transmodal hierarchy, showing: (i) positive effects at the unimodal gradient end, reflecting activation of visual/primary sensory areas; (ii) positive effects along intermediate and right-sided segments, indicating attentional (perceptually-coupled) and high-order executive processing; and (iii) a negative deflection at the transmodal gradient apex, capturing default-mode deactivation (all *P*_FDR_ < 0.05).

**Figure 3:**
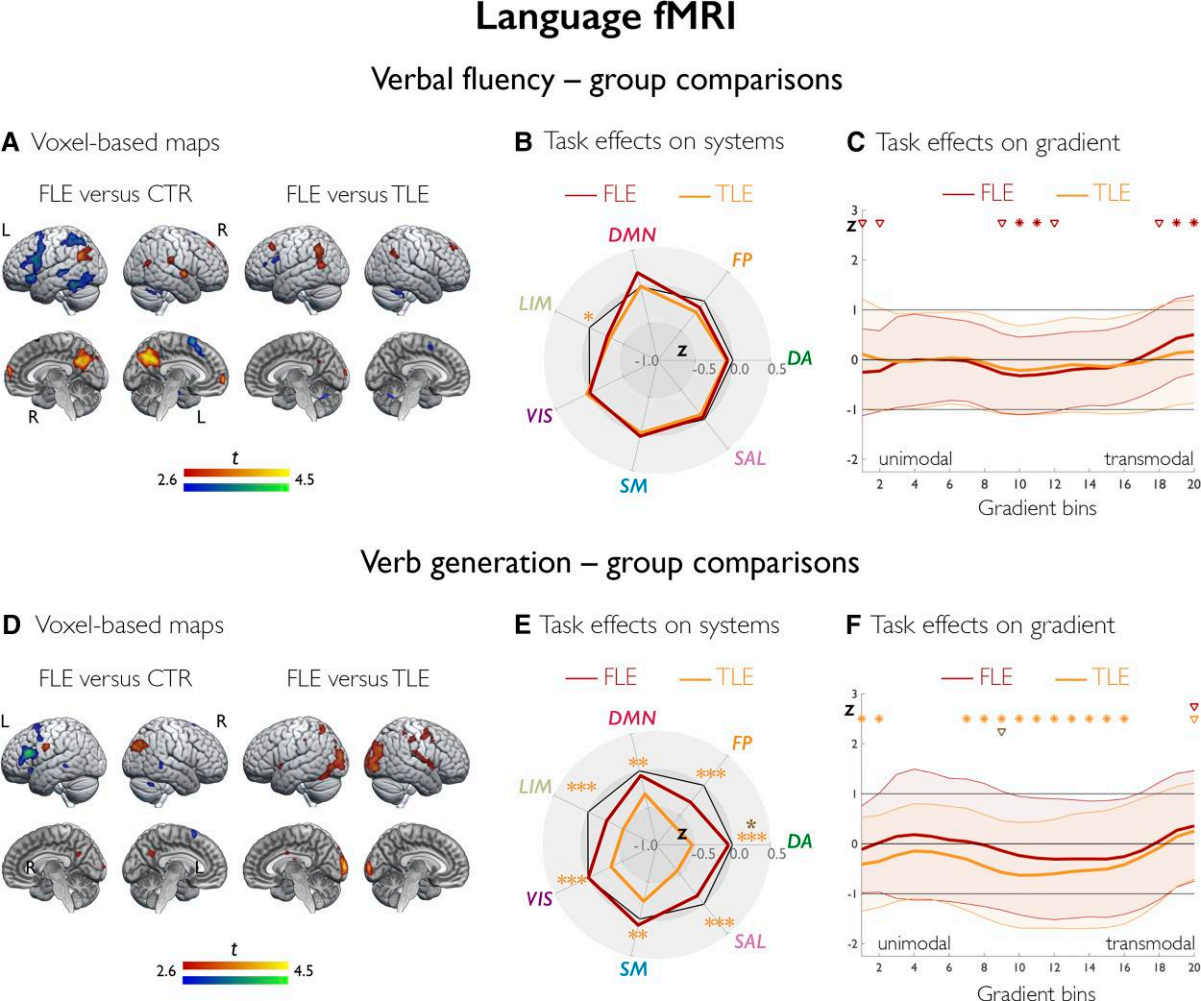
**Language fMRI: group comparisons.** Group comparisons for verbal fluency fMRI (**A**–**C**) and verb generation fMRI (**D**–**F**). In brain renders for comparison of individuals with FLE and controls (**A** and **D**), cold/warm colours refer to lower/higher task-related effects in patients; of note, increases in FLE exclusively mapped on areas undergoing task-related deactivation and are thus to be interpreted as areas of reduced deactivation in FLE versus controls; for comparison of FLE and TLE, cold/warm colour scales refer to lower/higher task-related effects in FLE relative to TLE. Group differences are shown at *P* < 0.005 uncorrected, with an extent threshold of 10 voxels applied for display purposes; colour bars indicate corresponding *t*-score scales, MNI coordinates and statistical details for differences within prespecified ROIs are provided in the Supplementary material. The spider plots (**B** and **E**) show *Z*-score analyses of task effects across seven systems (abbreviated as in Fig. 2). Across panels, black heptagons display effects in controls (*Z*-score = 0 for each system), and effects in FLE and TLE are shown in dark red and orange lines, respectively; red and orange asterisks denote FLE versus controls and TLE versus controls, while the brown asterisk in the verb generation spider plot highlights a difference between FLE and TLE; ***P_FDR_ < 0.01, **P_FDR_ < 0.05, *uncorrected *P* < 0.05. **C** and **F** show task-related effects, plotted as *Z*-scores (*y*-axis), stratified along the principal gradient, which was discretized into 20 consecutive, equally-sized bins (*x*-axis). Effects in FLE and TLE are shown in dark red and orange lines, respectively; for each group, shaded areas correspond to one standard deviation, red and orange asterisks/triangles denote FLE versus controls and TLE versus controls, respectively; brown asterisks/triangles refer to direct comparison of FLE and TLE; for each symbol, irrespective of colour; *P_FDR_ < 0.05, ^∇^uncorrected *P* < 0.05.

At the voxel-level (Fig. 3A), patients with FLE had reduced activation of left middle and inferior frontal gyrus, middle-anterior and middle-posterior temporal areas (*P*_FWE_ < 0.05), and reduced deactivation of bilateral anterior and posterior DMN regions, left posterior temporal and angular gyrus (*P*_FWE_ < 0.05) compared to controls. In TLE, there was reduced left inferior frontal activation and reduced deactivation of bilateral precuneus (*P*_FWE_ < 0.05) compared to controls (Supplementary Fig. 1). Patients with FLE had similar cortical activation to the TLE group, but lesser deactivation of posterior temporal and anterior DMN areas (*P*_FWE_ < 0.05). Across systems (Fig. 3B), there were no corrected differences between FLE and controls; sensitivity analyses across 17 systems highlighted impaired deactivation of DMN and frontoparietal control subdivisions in FLE than controls (DMN-A/DMN-C/control-C: *P*_FDR_ = 0.004/0.0025/<0.0001, *d* = 0.51/0.39/0.65; Supplementary Fig. 2). Curves of gradient-based task effects in FLE versus controls (Fig. 3C) showed (i) weaker task activity in intermediate gradient segments; and (ii) an increase at the transmodal apex, which implies lesser deactivation (all *P*_FDR_ < 0.05; *d* = *−*0.39 and *−*0.42 for the intermediate bins, *d* = 0.54 and 0.64 for the apex bins). Comparisons of TLE and controls and of FLE and TLE showed no corrected differences for analyses of systems and gradients.

### Verb generation fMRI

In controls, the verb generation task activated fronto-temporo-parietal cortices and subcortical areas (Fig. 2D); as distinct from verbal fluency, the left posterior temporal cortex and angular gyrus belonged to the task activation map. Activation involved frontoparietal control, DMN, salience and dorsal attention systems (β = 0.12/0.06/0.08/0.06, *P*_FDR_ < 0.0001/0.017/0.001/0.017, respectively; Fig. 2E). Gradient profiles (Fig. 2F) indicated extensive activation across the intermediate-to-transmodal segments (all *P*_FDR_ < 0.05).

At the voxel level (Fig. 3D), FLE exhibited reduced left inferior frontal activation and reduced right angular deactivation compared to controls (*P*_FWE_ < 0.05); in TLE, there were widespread fronto-temporo-parietal and occipital activation reductions compared to controls (*P*_FWE_ < 0.05; Supplementary Fig. 1). FLE had higher left posterior temporo-parietal and bilateral occipital activation, and lower deactivation of the right angular gyrus and bilateral precuneus than TLE (all *P*_FWE_ < 0.05). Analysis of systems (Fig. 3E) showed no corrected differences between FLE and controls or TLE; there was lower activity in TLE than controls, mostly encompassing dorsal attention, frontoparietal control and salience systems (all *P*_FDR_ < 0.0001; *d* = *−*0.60/*−*0.70/*−*0.62). Gradient curves (Fig. 3F) showed one positive deviation at the transmodal apex in FLE compared to controls (*P*_unc_ = 0.017, *d* = 0.32), while TLE differed from controls for global gradient-stratified profiles (FDA, permuted *P* = 0.046) and across most gradient bins (all *P*_FDR_ < 0.05; *d* range = *−*0.60 to *−*0.30). One intermediate bin showed higher task activity in FLE than TLE at an uncorrected threshold (*P*_unc_ = 0.048, *d* = 0.39).

### Verbal working memory fMRI

**Figure 4:**
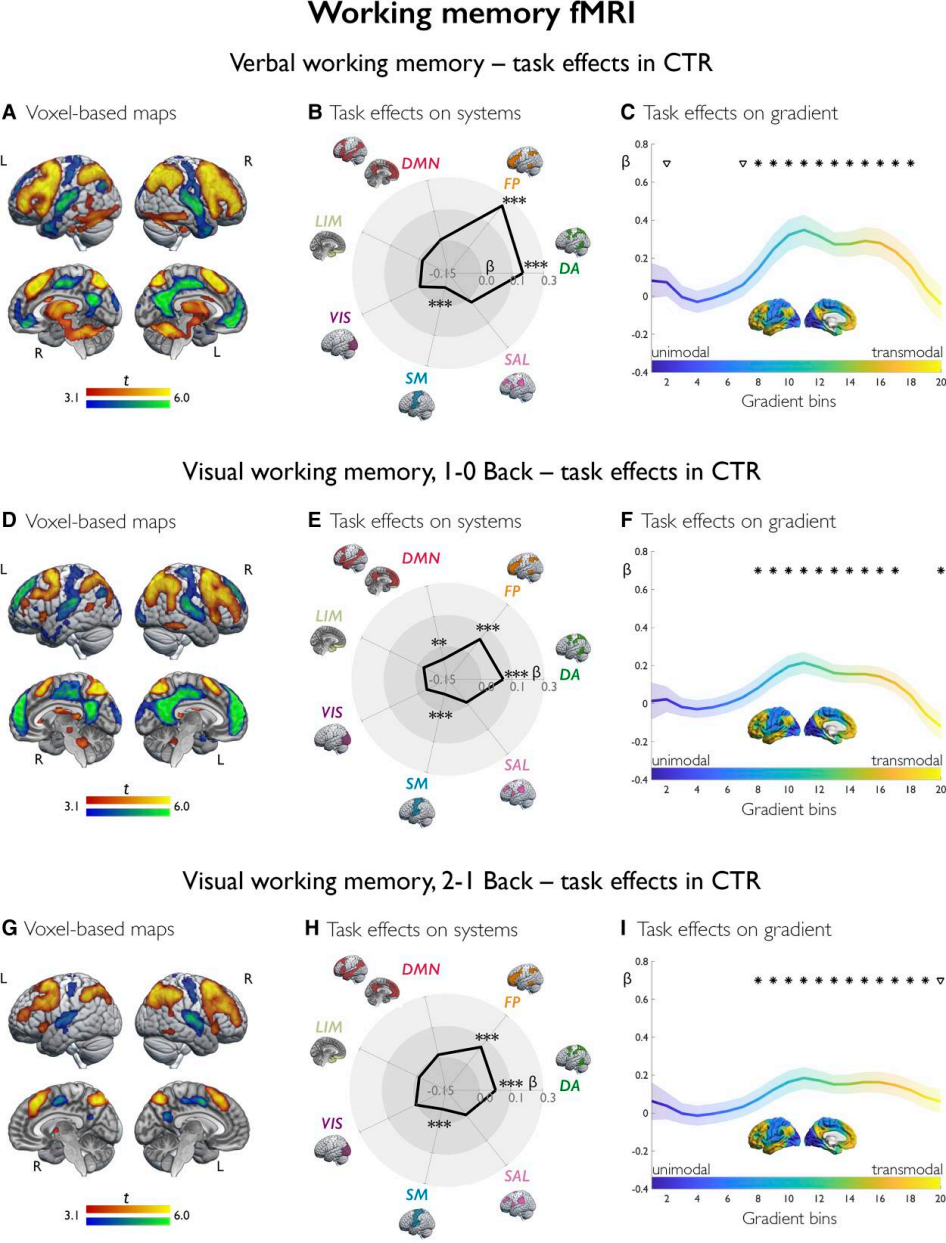
**Working memory fMRI: task effects in controls.** Across analytical scales, **A**–**C**, **D**–**F** and **G**–**I** show task effects in controls for verbal working memory, 1–0 Back visuo-spatial and 2–1 Back visuo-spatial working memory fMRI, respectively. **A**, **D** and **G** show task-related activation/deactivation (warm/cold colours) in controls, as derived from one-sample *t*-tests. Brain renders show maps at *P* < 0.001 uncorrected, with an extent threshold of 10 voxels applied for display purposes; colour bars indicate *t*-score scales. The spider plots (**B**, **E** and **H**) show mean task-related effects, parameterized as contrast estimates (β weights) across seven functional systems, where DA = dorsal attention; FP = frontoparietal control; LIM = limbic; SAL = salience (ventral attention); SM = somatomotor; VIS = visual. ***P_FDR_ < 0.01, **P_FDR_ < 0.05, *uncorrected *P* < 0.05. **C**, **F** and **I** show task-related effects stratified along the principal gradient; its left lateral and midline views are shown in the middle of each plot with the same colour scale as in curves of gradient-stratified task effects. The gradient was discretized into 20 consecutive, equally-sized bins (*x*-axis), with task-related signal (β weights; *y*-axis) computed per bin; shaded areas refer to 95% confidence intervals of mean effects at each bin. *P_FDR_ < 0.05, ^∇^uncorrected *P* < 0.05.

In controls, the verbal working memory task elicited bilateral frontoparietal activation (Fig. 4A), mapping on dorsal attention and control systems (β = 0.20/0.26, *P*_FDR_*<* 0.0001; Fig. 4B). Deactivation involved posterior cingulate cortex/precuneus, medial prefrontal and sensorimotor cortices (β = −0.08, *P*_FDR_ = 0.002 for somatomotor system effects). Gradient profiling (Fig. 4C) showed positive shifts along intermediate-to-transmodal segments, implicating attentional and executive processing, and decreases at the DMN apex (all *P*_FDR_ < 0.05).

**Figure 5:**
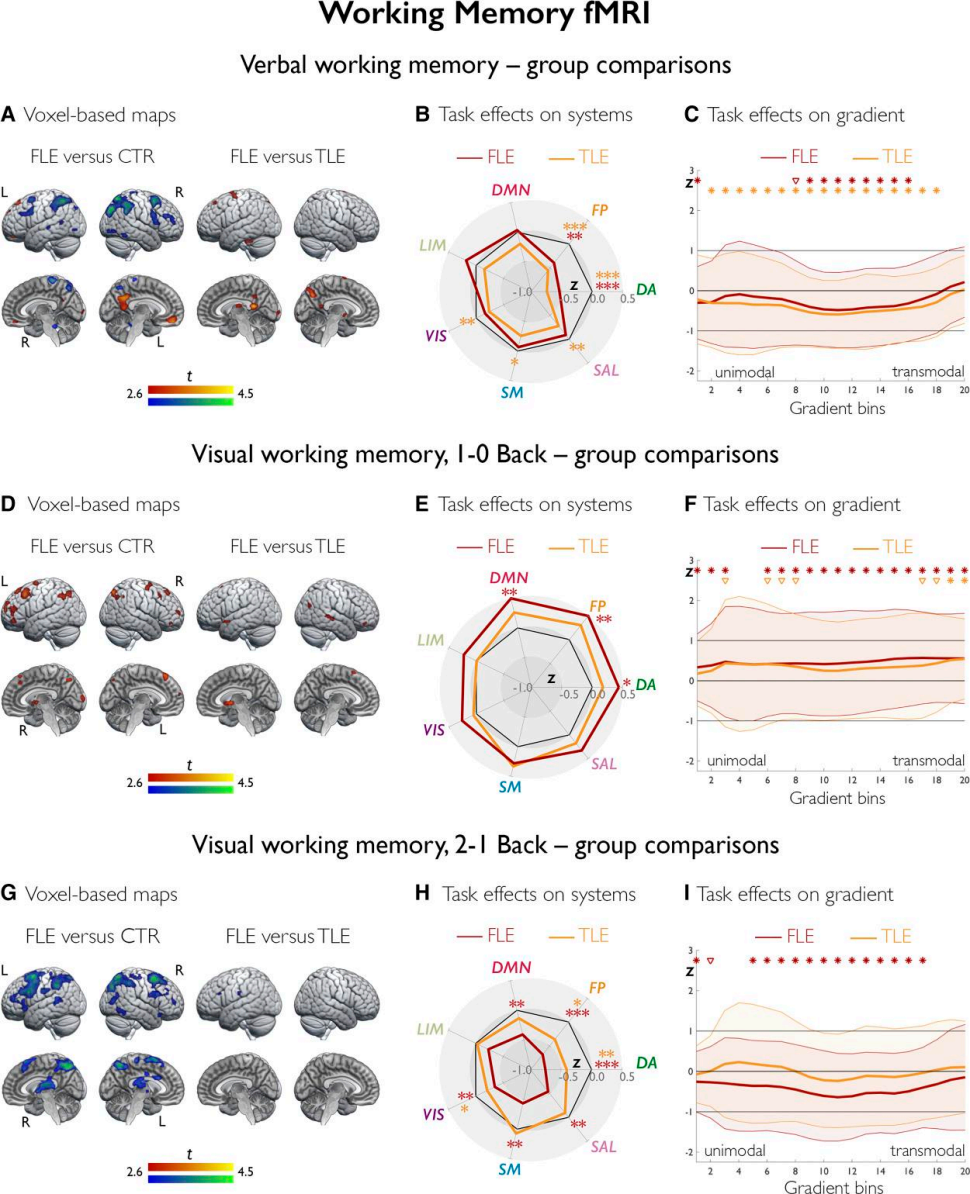
**Working memory fMRI: group comparisons. A**–**C**, **D**–**F** and **G**–**I** show group comparisons for verbal, 1–0 Back visuo-spatial and 2–1 Back visuo-spatial working memory fMRI, respectively. In brain renders (**A**, **D** and **G**) for comparison of patients with FLE and controls, cold/warm colours refer to lower/higher task-related effects in patients; for comparison of FLE and TLE, cold/warm colour scales refer to lower/higher task-related effects in FLE than TLE. Group differences are shown at *P* < 0.005 uncorrected, with an extent threshold of 10 voxels applied for display purposes; colour bars indicate corresponding *t*-score scales, MNI coordinates and statistical details for differences within prespecified ROIs are provided in the Supplementary material. The spider plots (**B**, **E** and **H**) show *Z*-score analyses of task-related signal across seven systems (abbreviations as in Fig. 4). Across panels, black heptagons display effects in controls (*Z*-score = 0 at each system), and effects in FLE and TLE are shown in dark red and orange lines, respectively; red and orange asterisks denote FLE versus controls and TLE versus controls. ***P_FDR_ < 0.01, **P_FDR_ < 0.05, *uncorrected *P* < 0.05. The *Z*-score for cognitive control task activity in FLE, 1–0 Back visual working memory, is 0.62; for display purposes, however, the spider plot axes reach a maximum of *Z* = 0.5. **C**, **F** and **I** show task-related effects, plotted as *Z*-scores (*y*-axis), stratified along the principal gradient, which was discretized into 20 consecutive, equally-sized bins (*x*-axis). Effects in FLE and TLE are shown in dark red and orange lines, respectively; for each group, shaded areas correspond to one standard deviation, red and orange asterisks/triangles denote FLE versus controls and TLE versus controls, respectively; brown asterisks/triangles refer to direct comparison of FLE and TLE; for each symbol, irrespective of colour, *P_FDR_ < 0.05, ^∇^uncorrected *P* < 0.05. Statistical details are provided in the main text.

At the voxel level (Fig. 5A), there was reduced frontoparietal activation and reduced deactivation of DMN areas in FLE versus controls (*P*_FWE_ < 0.05), and only reduced frontoparietal activation in TLE versus controls (*P*_FWE_ < 0.05; Supplementary Fig. 1). FLE showed less deactivation of posterior DMN areas than TLE (*P*_FWE_*<* 0.05). Analysis of systems (Fig. 5B) showed lower dorsal attention and frontoparietal control system activity in both FLE (*P*_FDR_ < 0.0001/0.019, *d* = *−*0.62/*−*0.40) and TLE (*P*_FDR_ < 0.0001/<0.0001, *d* = *−*0.82/*−*0.73) compared to controls. Gradient-stratified profiles (Fig. 5C) showed lower activity along intermediate gradient segments (*P*_FDR_ < 0.05; *d* range = *−*0.52 to *−*0.33) in FLE versus controls, and reduced activity across most gradient bins along with global differences in gradient profiles in TLE versus controls (*P*_FDR_ < 0.05; *d* range = *−*0.70 to *−*0.24; FDA, *P* = 0.030). There were no differences between FLE and TLE for analysis of systems and gradients.

### Visual working memory fMRI

In controls, the 1–0 Back contrast (Fig. 4D) elicited bilateral frontoparietal activation and deactivation of midline DMN areas. Contrasting high versus low working memory demands (2–1 Back) showed increasing frontoparietal recruitment (Fig. 4G). Analysis of systems (Figs. 4E and H) identified dorsal attention and frontoparietal control system activation (β = 0.11/0.09, *P*_FDR_*<* 0.0001/0.0002, 1–0 Back; β = 0.08/0.11, *P*_FDR_ = 0.005/<0.0001, 2–1 Back), DMN deactivation for the 1–0 Back contrast (β = −0.05, *P*_FDR_ = 0.023), and somatomotor deactivation for both contrasts (β = −0.08 and −0.06, *P*_FDR_*<* 0.0001 and 0.005, 1–0 Back and 2–1 Back). Gradient analyses (Fig. 4F and I) indicated positive activity shifts along its intermediate to transmodal segments and significant decreases at the default-mode apex (all *P*_FDR_ < 0.05).

For voxel-wise 1–0 Back contrast comparisons (Fig. 5D), there was increased parietal and dorsolateral frontal activation as well as reduced deactivation of anterior DMN areas in FLE compared to controls (*P*_FWE_ < 0.05), and reduced deactivation of anterior DMN areas in TLE than controls (*P*_FWE_*<* 0.05; Supplementary Fig. 1). Analysis of systems (Fig. 5E) showed higher frontoparietal control and DMN effects in FLE than controls (*P*_FDR_ = 0.015/0.026, *d* = 0.47/0.41) and no significant differences between TLE and controls. Gradient profiles (Fig. 5F) globally differed between FLE and controls (FDA, *P* = 0.022); bin-wise analyses showed higher task-related effects in FLE across most gradient sections (*P*_FDR_ < 0.05, *d* range = 0.31–0.51). In TLE, there was less deactivation than controls at the transmodal apex (*P*_FDR_ < 0.05, *d* = 0.48 and 0.55). There were no significant differences between FLE and TLE for voxel-based, system or gradient analyses.

For voxel-wise 2–1 Back contrast analyses (Fig. 5G), both FLE and TLE (Supplementary Fig. 1) showed less frontoparietal activation than controls (*P*_FWE_ < 0.05). Analysis of systems (Fig. 5H) showed pronounced negative systems-level deviations in FLE versus controls, particularly for dorsal attention and frontoparietal control systems (*P*_FDR_ < 0.0001/0.007, *d* = *−*0.68/*−*0.56); similar changes were observed for TLE versus controls (*P*_FDR_ = 0.028/0.055, *d* = *−*0.39/*−*0.33 for dorsal attention and frontoparietal control activity). Gradient-based profiles (Fig. 5I) showed global disorganization of task-related recruitment in FLE compared to controls (FDA, *P* = 0.034), with widespread involvement of intermediate and transmodal gradient segments (all *P*_FDR_ < 0.05; *d* range = *−*0.60 to *−*0.32). There were no suprathreshold differences between TLE and controls for gradient analyses, nor between FLE and TLE for voxel-based, system or gradient analyses.

### Correlation of fMRI measures with cognitive performance

**Figure 6:**
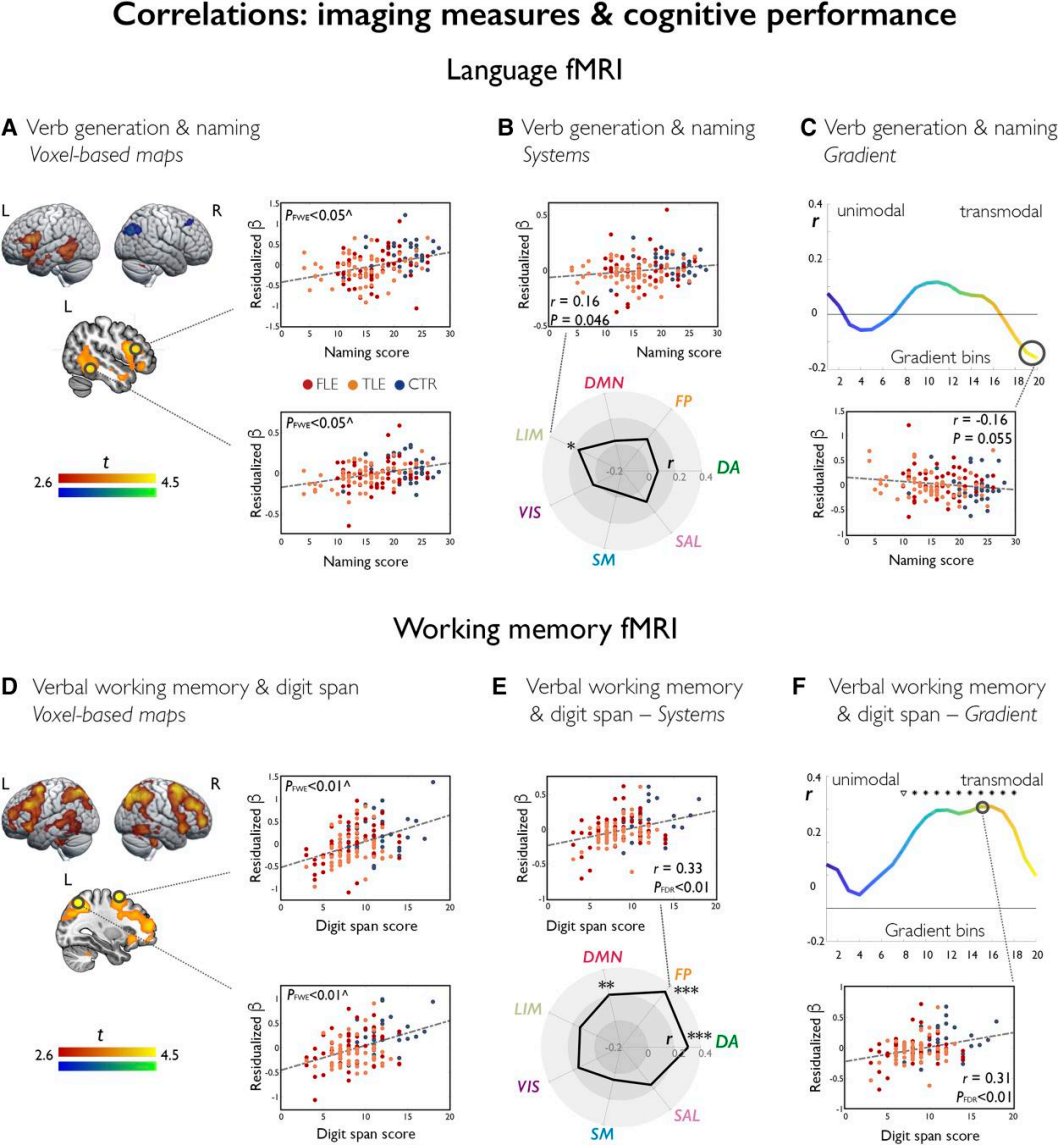
**Correlations of functional imaging measures with cognitive performance.** Brain renders and sections on the left display statistical maps of nonparametric multiple regressions probing associations between language fMRI (verb generation) and naming scores (**A**), and between working memory fMRI (verbal task) and digit span scores (**D**). Cold/warm colour scales refer to negative/positive associations, respectively. Maps are shown at *P* < 0.005 uncorrected, with an extent threshold of 10 voxels applied for display purposes; colour bars indicate corresponding *t*-score scales. ^Scatterplots highlight data distribution for the peak voxel within areas highlighted with a black circle; for illustration purposes, we used age- and sex-adjusted (residualized) contrast estimates (β) as measures of task effect. MNI coordinates and *P*-values are provided in the Supplementary material. The spider plots (**B** and **E**) and gradient plots (**C** and **F**) show correlation coefficients for associations between cognitive measures (naming/digit span) and task effects (verb generation/verbal working memory) across each system or gradient bin. Example scatterplots highlight data distribution for correlations at the level of one given system or bin; for analyses of systems: ***P_FDR_ < 0.01; **P_FDR_ < 0.05; *uncorrected *P* < 0.05; for analyses along the gradient: *P_FDR_ < 0.05; ^∇^uncorrected *P* < 0.05.

For language fMRI tasks, higher inferior frontal activation was associated with higher out-of-scanner verbal fluency and naming scores; naming also positively correlated with lateral temporal activation, particularly during verb generation fMRI (Fig. 6A). Conversely, lesser deactivation of bilateral precuneus during verbal fluency fMRI and right posterior temporal areas during verb generation fMRI related to lesser out-of-scanner fluency and naming performance, respectively (*P*_FWE_ < 0.05). Correlations across systems were limited (*r*_perm_ = 0.16, *P*_unc_ = 0.046, correlation of limbic system activity during verb generation fMRI and naming scores; Fig. 6B). For verbal fluency fMRI, gradient-based effects at the transmodal apex negatively correlated with out-of-scanner letter fluency scores (*r*_perm_ = *−*0.17/*−*0.19, *P*_unc_ = 0.036/0.026).

For verbal working memory fMRI, out-of-scanner digit span scores positively correlated with (i) bilateral frontoparietal activation during verbal working memory (*P*_FWE_ < 0.05; Fig. 6D); (ii) activity across dorsal attention and frontoparietal control systems (*r_perm_* = 0.31 and 0.33, respectively; both *P*_FDR_ = 0.0007; Fig. 6E); and (iii) task signal across intermediate and transmodal gradient sections (all *P*_FDR_ < 0.01, *r_perm_* range: 0.24–0.31; Fig. 6F). Similar patterns were evidenced for correlations between verbal 2 Back task performance scores (in the scanner) and verbal working memory fMRI activity across (i) dorsal attention/frontoparietal control systems (*ρ_perm_* = 0.23/0.21, *P*_FDR_ = 0.020/0.027); and (ii) intermediate-to-transmodal gradient segments (*P*_FDR_ < 0.05, *ρ*_perm_ range: 0.20–0.23).

For 2-1 Back visual working memory fMRI, visual 2 Back task performance scores (in the scanner) positively correlated with: (i) bilateral frontoparietal activation at the voxel level (*P*_FWE_ < 0.05); (ii) activity across dorsal attention and frontoparietal control systems (*ρ_perm_* = 0.37 and 0.39; both *P*_FDR_ < 0.0001); and (iii) task signal across intermediate and transmodal gradient sections (all *P*_FDR_ < 0.05, *ρ*_perm_ range: 0.20–0.36).

### Correlation of fMRI measures with clinical variables

**Figure 7:**
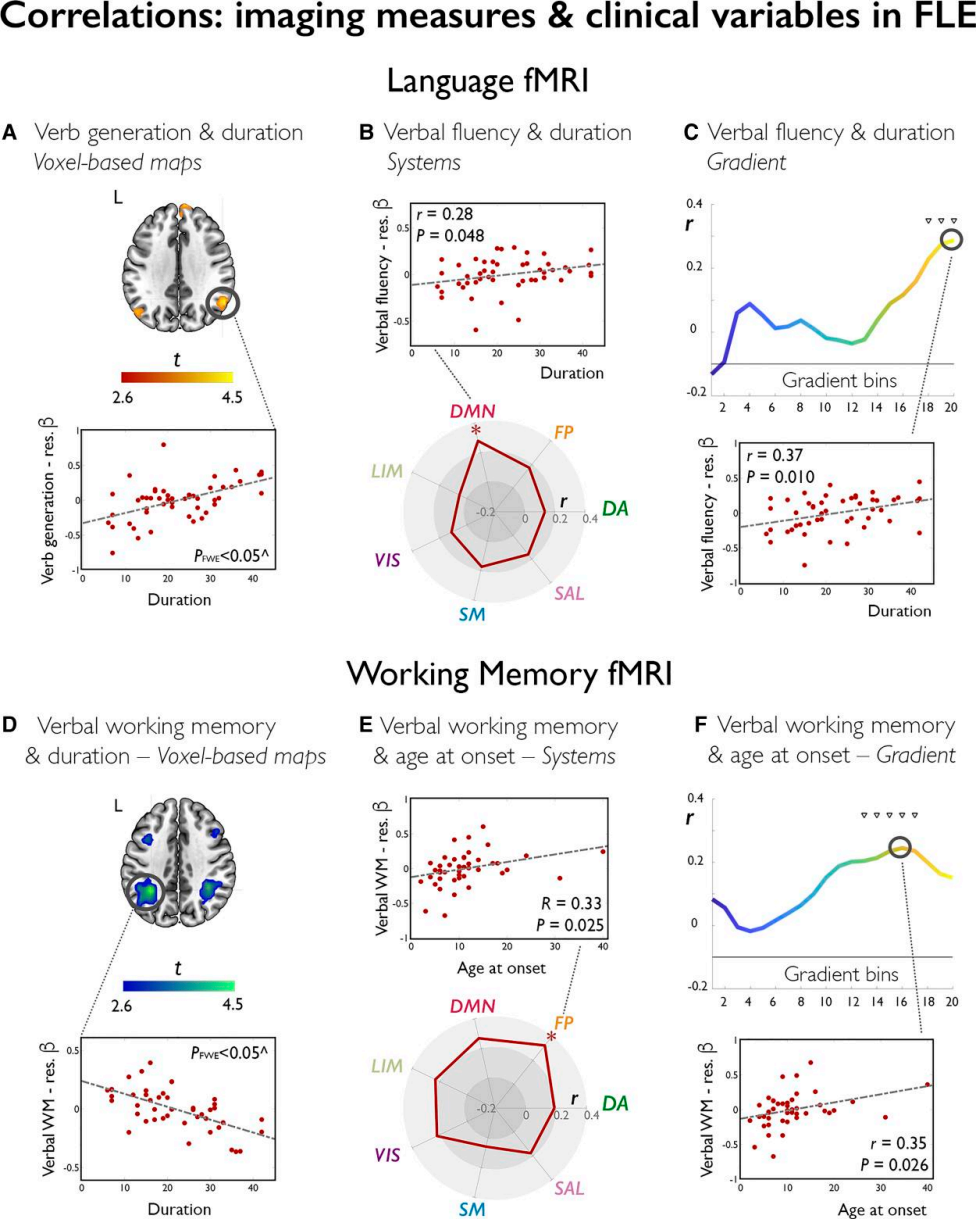
**Correlations of functional imaging measures with clinical variables in FLE.** Brain sections on the left show statistical maps of nonparametric multiple regressions probing associations between language or working memory fMRI and duration of epilepsy in FLE. For language (**A**), we show positive associations between duration of epilepsy and task effects during verb generation, where significant voxels (right posterior temporal/angular ROI, peak *P*_FWE_ < 0.05; highlighted in black circle) map on areas undergoing task related deactivation; a positive association (warm colour) thus indicates reduced deactivation with longer duration of disease. For working memory (**D**), we show areas of negative associations between frontoparietal activation and disease duration with cold colours. Maps are shown at *P* < 0.005, with an extent threshold of 10 voxels applied for display purposes; colour bars indicate *t*-score scales. ^Scatterplots highlight data distribution for the peak voxel within areas highlighted with a black circle; for illustration purposes, we used age- and sex-adjusted (residualized) contrast estimates (β) as measures of task effect. MNI coordinates and *P*-values are provided in the Supplementary material. The spider plots (**B** and **E**) and gradient plots (**C** and **F**) show correlation coefficients for associations between epilepsy duration and task effects for verbal fluency fMRI (**B** and **C**), and between age at onset and verbal working memory fMRI (**E** and **F**), across each canonical system or gradient bin. Example scatterplots highlight data distribution for correlations at the level of one given system or one given bin; for analyses of systems: ***P_FDR_ < 0.01; **P_FDR_ < 0.05; *uncorrected *P* < 0.05; for analyses along the gradient: *P_FDR_ < 0.05; ^∇^uncorrected *P* < 0.05.

For language tasks in FLE, longer epilepsy duration related to: (i) higher verbal fluency fMRI activity in a rostral left middle frontal area bordering the classical activation map (*P*_FWE_ < 0.05), possibly reflecting compensatory recruitment; (ii) lesser deactivation of the whole DMN (*r*_perm_ = 0.28, *P*_unc_ = 0.049; Fig. 7B); and (iii) lesser deactivation at the gradient apex (bins 18–20, *r*_perm_ range = 0.31–0.37, *P*_unc_ = 0.011–0.029; Fig. 7C). For verb generation, longer duration related to lesser posteriortemporal and angular deactivation (*P*_FWE_ < 0.05; Fig. 7A); longer time since last seizure correlated with lower anterior temporal activation (*P*_FWE_ < 0.05), while higher seizure frequency was associated with lower deactivation at the gradient apex (bins 19–20, *r*_perm_range = 0.28–0.29, *P*_unc_ = 0.046–0.040). For working memory in FLE, we found a significant association between lower bilateral parietal activation during the verbal task and longer disease duration (*P*_FWE_ < 0.05; Fig. 7D). This was paralleled by positive correlations between age at onset and (i) frontoparietal control activity (*r*_perm_ = 0.33, *P*_unc_ = 0.025; Fig. 7E); as well as (ii) gradient-based profiles (bins 12–18, *r*_perm_ range = 0.30–0.35, *P*_unc_ = 0.026–0.048; Fig. 7F) during verbal working memory. History of FBTCS related to lower right dorsolateral frontal activation (*P*_FWE_ < 0.05).

For language tasks in TLE, earlier age at onset was associated with lower inferior frontal activation during verbal fluency. Complementing our prior work,^73^ history of FBTCS was associated with lower activation of temporal, angular and DMN areas during verbal fluency (*P*_FWE_ < 0.05), mapping onto (i) dorsal attention, frontoparietal control, DMN, limbic, visual and somatomotor systems (*r*_perm_ = *−*0.33/*−*0.29/*−*0.36/*−*0.55/*−*0.34/*−*0.45, *P*_FDR_ = 0.010/0.026/0.010/<0.001/0.010/0.010); and (ii) most gradient bins (bins 2–20, *r*_perm_range = *−*0.28 to *−*0.39, *P*_FDR_ = 0.016–0.033). For verb generation, history of FBTCS positively related to right posterior temporal activation (*P*_FWE_ < 0.05), possibly reflecting compensatory recruitment. For working memory in TLE, FBTCS were associated with effects across premotor/precentral areas (*P*_FWE_ < 0.05) during the verbal task. For visual working memory (1–0 Back), longer duration related to lesser deactivation of posterior DMN areas (*P*_FWE_ < 0.05); for the 2–1 Back contrast, longer time since last seizure related to higher activity of (i) dorsal attention, salience and somatomotor systems (*ρ_perm_* = 0.33/0.36/0.38, *P*_FDR_ = 0.034/0.020/0.020); and (ii) unimodal to intermediate gradient sections (bins 3–9, *ρ*_perm_ range = 0.35–0.39, *P*_FDR_ = 0.019–0.023).

### Sensitivity analyses

Extensive sensitivity analyses (detailed in the Supplementary material) corroborated the robustness of the above results to (i) different systems-level parcellation; (ii) pathology-informed subgroup allocation (dysplasia-related FLE); (iii) lesional status; (iv) frontal language laterality; and clinical characteristics such as (v) laterality of seizure focus; (vi) seizure frequency; (vii) history of FBTCS; and (viii) time since last seizure.

## Discussion

Knowledge of the neural substrates of cognitive impairment in FLE is scarce. Here, we profiled the neural correlates of language and working memory impairment in a large FLE sample. Additional analysis of a TLE group allowed decoding shared and syndrome-specific effects. Our findings indicate impaired fronto-temporo-parietal activation and impaired DMN deactivation in FLE, with global disorganization of task-related recruitment during working memory, and implicate areas across a broad spectrum of functional specialization. While patterns of impairment largely overlapped across syndromes, we found more prominent alterations of DMN deactivation in FLE, and more marked alterations of temporal activity in TLE during verb generation, which entails semantic processing. Task-related functional signatures were detrimentally modulated by clinical characteristics both in FLE and TLE. This study conveys a comprehensive characterization of the neural correlates of cognitive impairment in FLE, paving the way for future analyses of disease subgroups. We identify neural targets that may aid future investigations into cognitive prognostics and inform the development of novel rehabilitation and therapeutic approaches.

Neuropsychological profiling indicated generalized cognitive impairment in FLE, with poorer performance for functions typically ascribed to the frontal lobes, including working memory, verbal fluency and mental flexibility, as well as weaker verbal memory and naming, processes that rely more markedly on temporal lobe function. Comparison of FLE and TLE showed more prominent executive dysfunction in the former, and more marked impairment of verbal learning and semantic knowledge in the latter. Our findings corroborate evidence of dysexecutive traits and memory difficulties in FLE^8,9,11^ and indicate that cognitive profiles in FLE and TLE differ. Echoing prior work,^4,7,14^ we suggest that functions that more strongly recruit areas overlapping with the epileptic network are affected more pervasively.

Traditional voxel-based fMRI maps provide fine-grained accounts of regional group differences but cannot capture large-scale effects. Using a multiscale approach, we attained systems-level inference^35,71,76^ and profiled the landscape of brain activation and deactivation patterns on the backbone of the principal gradient of intrinsic connectivity.^36,77^ The gradient describes a continuous transition from unimodal sensory to transmodal areas, offering a compact framework to probe task-related effects in the context of cognitive system hierarchies and detect global differences in cognition-related brain activity.^39,40,66^ Our analyses thus enabled decoding of the neural signatures of cognitive processes and related functional reorganization in epilepsy, capitalizing on a ‘local-to-global’ perspective.

In controls, language tasks elicited left-lateralized activation of middle frontal, inferior frontal, and temporo-parietal cortices, which positively correlated with fluency and naming scores. Systems-level effects captured frontoparietal control and opercular involvement, and activity shifts along intermediate-to-transmodal gradient segments implicated a combination of attentional and high-level executive processing. Attenuation of DMN activity was extensive for verbal fluency, which requires executive control,^15,78^ was tracked by negative effects at the gradient apex, and was neurobehaviourally relevant, as demonstrated by correlations analyses with out-of-scanner performance. Temporo-parietal activation was marked during verb generation, owing to its semantic demands,^79,80^ and correlated with naming scores.

Across language tasks, we found reduced left inferior frontal and middle frontal activation in FLE. Frontal language lateralization was also weaker in FLE than controls, corroborating findings of prior case series.^81,82^ Repeat comparisons controlling for language laterality, however, excluded a substantial influence of interhemispheric frontal language organization on the observed group differences. Thus, dysfunction of the left frontal language core may be a key feature underlying impaired expressive language in FLE. From a neurobiological perspective, such dysfunction may stem from epileptic activity of frontal origin, which may have adverse effects on synaptic connections and local computation. Exploratory analyses identified similar disruptions of left-hemispheric activation in left and right FLE. Involvement of left-sided language areas and verbal deficits were previously described in people with right TLE, which led to reconsider earlier views on the sparing of verbal functions in right-hemisphere pathology.^15,83–85^ Similarly, it is possible that left frontal language alterations in right FLE may be a consequence of transcallosal propagation of epileptic activity,^86^ given the rapid bilateral spread of frontal lobe seizures.^1,87^

FLE and TLE groups had similar frontal abnormalities during verbal fluency. Frontal language dysfunction thus represents a shared trait, possibly a downstream consequence of both proximal (frontal) and more distal (temporal) pathology. Notably, we and others previously showed abnormal frontal activation and frontotemporal connectivity during expressive language in TLE,^21,22,88,89^ which may emerge as a propagated abnormality, mediated by microstructural alterations in perisylvian white matter tracts.^58,84,85^ Differences between FLE and TLE in the semantic processing stream during verb generation, involving posterior temporo-parietal and occipital areas,^80,90,91^ indicate that impairment during tasks with semantic demands is TLE-specific, providing a correlate to neuropsychological findings. While frontal language areas represent a common substrate of expressive language difficulties in TLE and FLE, temporal pathology may primarily affect areas in its vicinity, such as posterior language centres, leading to more pervasive dysfunction during tasks that specifically rely on these.

In both working memory tasks, we detected bilateral frontoparietal activation^30,92^ that mapped on dorsal attention and frontoparietal control systems, resulted in activity shifts in the intermediate-to-transmodal gradient sections, and strongly correlated with cognitive scores. For both conditions entailing a 2 Back working memory span, individuals with FLE exhibited bilateral attenuation of frontoparietal activation and altered gradient profiles. Activation reductions were particularly marked for the 2–1 Back contrast and affected cognitive systems beyond those directly implicated in working memory. While indicating global dysfunction, such widespread effects may also reflect loss of motivation and participant disengagement. The verbal working memory task, however, was less challenging and, though performed slightly less accurately by people with FLE than controls, resulted in satisfactory performance (>80%) in all groups. Patients with FLE still presented with frontoparietal hypoactivation, more selective for dorsal attention and cognitive control systems. Thus, we suggest that attenuated frontoparietal activation may more parsimoniously reflect inefficient recruitment of areas required for successful task performance. The low-demand visual working memory contrast highlighted enhanced frontoparietal activation and reduced DMN deactivation in FLE. This sequence of higher and lower activation for easy and difficult task conditions thus points to cognitive system saturation already occurring for low-level task demands, followed by defective additional recruitment for higher task difficulty. Notably, by encompassing altered deactivation of DMN regions, neural processes underlying working memory in FLE already proved inefficient for easier task conditions.

Comparison of FLE and TLE for working memory activation did not indicate marked group differences. Working memory processing engages distributed, bilateral fronto-temporo-parietal networks.^34,93,94^ Propagation of ictal and interictal epileptic activity may lead to long-lasting neural derangements within multiple sites relevant for performance, which all result in less efficient working memory networks, independent of the location of the epileptic focus. Future investigation of recent-onset focal epilepsy may clarify whether the involvement of working memory hubs may be sequential, with earlier effects close to the seizure onset zone.

Across tasks, we showed impaired deactivation of DMN areas in FLE, and to a lesser extent in TLE. The DMN subserves processes including self-awareness, mind-wandering and cognition supported by internal representations.^95–97^ DMN deactivation and anti-correlation between DMN and frontoparietal activity were described for executive tasks.^32,98,99^ Alterations in such processes were previously identified in psychiatric disorders, including autism^100,101^ and schizophrenia,^102,103^ and may reflect suboptimal distribution of neural resources during goal-directed cognition.^104^ We and others previously showed altered DMN deactivation during working memory in juvenile myoclonic epilepsy and paediatric TLE.^55,105^ Our current findings in FLE underscore the vulnerability of the DMN across the epilepsy spectrum, and indicate altered DMN deactivation profiles as a possible trans-syndromic marker. Notably, while intergroup differences encompassed DMN areas, global alterations, affecting the whole DMN, only emerged for visual working memory. The spatially widespread, distributed nature of the DMN in the seven-system partition, along with recent evidence that DMN subsections have distinct functional roles,^106^ are possible reasons for the lack of group differences cohesively involving the DMN in language tasks.^35^ In addition, we and others previously documented an influence of ASMs on task-related deactivation during language and working memory.^46,47,107–111^ Such effects, however, inconstantly involved those midline anterior and posterior DMN areas that were sites of prominent differences between FLE and controls and between FLE and TLE in this study.

Interestingly, alterations of task-related deactivation, mostly encompassing DMN nodes, were more marked in FLE than TLE. A possible explanation may lie in the differential connectivity profiles of specific DMN hubs, such as the medial prefrontal cortex, that is strongly embedded within midline DMN, and also likely to be involved in the propagation network of frontal seizures.^87^ Covarying for clinical characteristics did not alter working memory findings but modulated angular and posterior DMN deactivation differences between FLE and TLE during verb generation. Thus, our findings suggest that epilepsy severity may represent an additional contributing factor to differences in language system architecture in FLE and TLE.

Correlation analyses showed that disease load and factors associated with severity, as tracked by age at onset, epilepsy duration, history of FBTCS and time since last seizure, may modulate the degree of both activation and deactivation profiles in FLE, extending prior work in TLE.^73,74^ In FLE, effects across areas of activation were particularly evident for working memory, while clinical variables more strongly influenced deactivation patterns during language. Prior work identified detrimental associations between cognitive function and early age at epilepsy onset,^112–115^ linking them to disrupted white matter maturation and connectivity.^116,117^ In our study, associations in FLE were marked for functions with a prolonged maturational trajectory, such as working memory, which depends on the myelination of long-range fibre tracts.^118,119^ Future longitudinal work may shed further light on associations between clinical, cognitive and neural profiles and their joint modulation by ASMs. Finally, we note that the co-occurrence of impaired frontoparietal activation and DMN deactivation in FLE mirrors prior comparisons of healthy older adults (∼70 years) to younger individuals.^120,121^ As the mean age in our FLE sample was ∼30 years, it is tempting to speculate that the identified traits may reflect a functional marker of accelerated brain aging. If proven by future work, such phenomenon would dovetail with recent evidence of accelerated grey matter loss in people with focal epilepsy, which revived the ‘disease progression’ hypothesis.^122–125^

Our study has several strengths, including the use of large samples, robust methodology, comprehensive mapping of function from local to global perspectives, direct comparison of patient groups and several sensitivity analyses. Our study also has limitations. The FLE group was heterogeneous in terms of aetiology and MRI findings but representative of the spectrum of FLE patients assessed in tertiary centres.^3,8,126^ Sensitivity analyses in the focal cortical dysplasia-related FLE subgroup largely corroborated our main findings. Further subgroup analyses showed similar differences in task-related effects in lesional and non-lesional FLE compared to controls. However, direct comparison of patient subgroups provided preliminary evidence for a less efficient functional architecture in lesional FLE, with relative enhancement of lateral temporal activity during language and higher dysfunction of frontal circuitry subserving working memory, possibly in light of its more prolonged maturational trajectories.^118,119^ Future work is encouraged to disentangle cognitive alterations specific to distinct aetiologies, grouping larger patient samples based on sub-lobar lesion location or seizure semiology.^87^ The language tasks were covert, which prevented online performance monitoring. However, these tasks were previously validated,^58,88^ are extensively used clinically^56,127^ and captured interindividual differences in out-of-scanner fluency and naming performance in our study. Future studies may benefit from using overt language paradigms that allow investigators to monitor compliance and task performance,^20,21,128^ which would provide assessments of functional reconfigurations directly subserving task execution. Notably, systems-level analyses did not capture differences between FLE and controls during language tasks. While localized frontal language dysfunction in FLE may simply not translate to large-scale, bilateral abnormalities, thus lacking a systems-level correlate, we also note that the landmark functional atlas we utilized^35^ lacks a dedicated language partition, which may have decreased the sensitivity of our analyses. Future work may benefit from employing recently developed functional parcellations that also incorporate a language system.^129^ Individuals with FLE and TLE were drug-resistant, taking ASMs in various combinations. We and others previously described compound- and syndrome-specific effects of ASMs on cognitive networks.^107,109–111,130^ Patient groups were however balanced for medications with known detrimental (topiramate/zonisamide) or more favourable (levetiracetam) cognitive network profiles.^46,47^ As for comparisons with controls, it is possible that impaired task-related activation in FLE (and TLE) may be partially influenced by ASMs. As discussed earlier, however, ASM-related effects on task deactivation patterns inconsistently involve the midline areas that showed marked intergroup differences in this study. Finally, we identified associations of age at seizure onset, disease duration, FBTCS and time since last seizure with task-related imaging phenotypes, which suggests disease-related mediating factors other than ASMs. Future longitudinal work, including the assessment of drug-naïve patients, may better characterize the contribution of ASMs to cognitive system reorganization in epilepsy.

In conclusion, our study decodes neural processes underlying language and working memory impairment in FLE, showing local, systems-level and global abnormalities that indicate an altered interplay between task-related cognitive system activation and deactivation. While patterns of dysfunction in FLE and TLE largely overlap, activity of posterior language centres is more affected in TLE relative to FLE, and profiles of default-mode deactivation are more impaired in FLE. This work bridges a substantial knowledge gap in the epilepsy literature and delivers neural markers that can be validated in the context of cognitive prognostics, serving as targets for future development of targeted treatments.

## Supplementary Material

awac150_Supplementary_Data
