# Supplementary material for: Disorganization of language and working memory systems in frontal versus temporal lobe epilepsy
Source: Brain. 2022 May 2;146(3):935–53. doi: 10.1093/brain/awac150 (PMC9976988; doi:10.1093/brain/awac150)
Supplement: awac150_Supplementary_Data [file awac150_supplementary_data.zip › brain-2021-00401-File010.pdf]

Online Supplementary Material for:

Disorganization of language and working memory systems in frontal versus temporal lobe epilepsy

Lorenzo Caciagli,[1,2,3] Casey Paquola,[4] Xiaosong He,[1] Christian Vollmar,[2,3,5] Maria Centeno,[2,3,6] Britta Wandschneider,[2,3] Urs Braun,[1,7] Karin Trimmel,[2,3,8] Sjoerd Vos,[2,3,9,10] Meneka Sidhu, [2,3] Pamela J. Thompson,[2,3] Sallie Baxendale,[2,3] Gavin P. Winston,[2,3,11] John S. Duncan,[2,3] Dani S. Bassett,*[1,12,13,14,15,16] Matthias J. Koepp,*[2,3] and Boris C. Bernhardt *[4]

* denotes equal contribution as senior author

[1] *Department of Bioengineering, University of Pennsylvania, Philadelphia, Pennsylvania 19104 USA*
[2] *Department of Clinical and Experimental Epilepsy, UCL Queen Square Institute of Neurology, London WC1N 3BG, United Kingdom*
[3] *MRI Unit, Epilepsy Society, Chalfont St Peter, Buckinghamshire, SL9 0RJ United Kingdom*
[4] *Multimodal Imaging and Connectome Analysis Laboratory, McConnell Brain Imaging Centre, Montreal Neurological Institute, Quebec H3A 2B4 Canada*
[5] *Department of Neurology, Ludwig-Maximilians-Universität, Marchioninistrasse 15, 81377 Munich, Germany*
[6] *Epilepsy Unit, Hospital Clínic de Barcelona, Carrer de Villaroel 170, 08036 Barcelona, Spain*
[7] *Department of Psychiatry and Psychotherapy, Central Institute of Mental Health, Medical Faculty Mannheim, University of Heidelberg, Mannheim*
[8] *Department of Neurology, Medical University of Vienna, Vienna, Austria*
[9] *Centre for Medical Image Computing, University College London, London, United Kingdom*
[10] *Neuroradiological Academic Unit, UCL Queen Square Institute of Neurology, University College London, London, UK*
[11] *Department of Medicine, Division of Neurology, Queen's University, Kingston, Ontario, Canada*
[12] *Department of Physics and Astronomy, University of Pennsylvania, Philadelphia, Pennsylvania 19104 USA*
[13] *Department of Electrical and Systems Engineering, University of Pennsylvania, Philadelphia, Pennsylvania 19104 USA*
[14] *Department of Neurology, University of Pennsylvania, Philadelphia, Pennsylvania 19104 USA*
[15] *Department of Psychiatry, University of Pennsylvania, Philadelphia, Pennsylvania 19104 USA*
[16] *Santa Fe Institute, Santa Fe, New Mexico 87501 USA*

Running title: Mapping cognitive dysfunction in FLE and TLE

SUPPLEMENTARY METHODS

Functional MRI data: quality checks

Excessive motion (mean framewise displacement >0.5mm)[61] was observed in 3/1/3/1 FLE, 5/4/4/3 TLE, and 2/2/1/2 control participants for visual WM/verbal WM/verbal fluency/verb generation fMRI, respectively. Individual task maps were reviewed to rule out gross artefact, which led us to exclude visual WM fMRI data for 1/1 FLE/TLE patient, and verbal WM fMRI data of 1 FLE patient. In one FLE patient, voxels within a frontal DNET were assigned β values of "not a number" during SPM-based model specification; these were subsequently operationalized as zeros. Language paradigms were covert, and performance measures for in-scanner task execution were not available. As previously,[107,e1] individual-level activation of task relevant areas, such as frontotemporal regions, cerebellum, supplementary motor area and anterior insula, was verified up to an uncorrected threshold ($p<0.01$); lack thereof led to participant exclusion. We further excluded verbal working-memory fMRI data of one TLE patient, and verb generation fMRI data of one control owing to heavily corrupted field of view. Final analyses for visual WM/verbal WM/verbal fluency/verb generation fMRI included 46/51/53/55 FLE patients, 56/58/59/60 TLE patients, and 50/49/49/47 controls.

Structural data: acquisition protocol and surface-based pre-processing

Structural images were only used to align individual functional space to structural space before gradient-based fMRI analyses in surface space. T1-weighted images were acquired on the same scanner as the functional images, using a coronal 3D fast-spoiled-gradient-echo (FSPGR) sequence, matrix 256×256, slice thickness 1.1mm, in-plane resolution 1.1×1.1mm. In patients with TLE and in a healthy control subset (n=27), T1-weighted images had a slightly different in-plane resolution of 0.9375×0.9375mm; this was not associated with differences in MRI contrast between grey and white matter, and did not influence the accuracy of cortical surface extraction. In each participant, we derived cortical surfaces using previously validated FreeSurfer pipelines (v6.0; http://surfer.nmr.mgh.harvard.edu/),[e2,e3] which include reorientation, skull-stripping, tissue segmentation, and generation of pial and white matter surfaces. T1-weighted data for 1 FLE patient and 1 control were affected by motion artefacts; these subjects were thus discarded.

For gradient-based stratification of functional data, task-related general linear models were computed in native space after SPM-based realignment for each subject, with task contrast specifics as detailed in the main text, and 6 motion parameters included as nuisance regressors.

We co-registered cortical surfaces with mean functional and task contrast images via boundary-based registration,[e4] followed by spherical mapping to the *fsaverage5* surface template (20484 vertices), and surface smoothing of the functional contrasts with a Gaussian filter of 5mm FWHM.

Voxel-based fMRI, group comparisons: regions of interest and further statistical details

For language tasks, we assessed voxel-based group differences within frontal and temporo-parietal regions of interest (ROIs),[22,e5,e6] previously defined functionally via a language-localizer fMRI paradigm in healthy participants:[e5] (i) orbital inferior frontal gyrus, (ii) inferior frontal gyrus, (iii) middle frontal gyrus, (iv) anterior and (v) middle-anterior temporal lobe, (vi) middle-posterior and (vii) posterior temporal lobe, as well as (viii) angular gyrus. These ROIs were mirrored onto the right hemisphere to create homologous masks. For group comparisons, we thus used 8 bilateral ROIs. Frontal language ROIs were part of the activation map in both language tasks, while task-related effects in anterior, middle-anterior, middle-posterior temporal and angular ROIs occurred either in the form of activation or deactivation, depending on the specific task. For each language task, intergroup differences within such ROIs are thus discussed in the form of activation/deactivation differences based on their behavior during the task in healthy controls.

For WM tasks, we employed following bilateral ROIs, based on prior meta-analyses:[30,92] (i) one frontal-eye field/premotor [Brodmann areas (BA) 8 and 6 (dorsal)]; (ii) dorsolateral/ ventrolateral prefrontal (BA 9/46 and 44); and (iii) one dorsal parietal, including dorsal precuneus, superior parietal lobule, inferior parietal lobule (part), and rostral lateral superior occipital gyrus (BA 7, 39 and 40). ROIs were obtained by merging the relevant Brodmann labels, provided via the *Brainnetome* Atlas.[e7]

For both tasks, we included midline default-mode (DMN) areas, belonging to the deactivation map, henceforth labeled as "task-negative" ROIs:[47,55,120,e8] (i) midline prefrontal (anterior task-negative), combining BA 32 (anterior cingulate), 9/10 (rostral medial superior frontal), 11 and 14 (medial frontal/orbital); and (ii) midline parietal (posterior task-negative), including precuneus and posterior cingulate cortex (BA 31 and 23). For language tasks, the midline parietal ROI additionally included the dorsal part of precuneus (BA 7), which has instead been described as part of the activation network for WM tasks,[30] and was therefore not included in the posterior DMN ROI for WM.

Voxel-based fMRI, language laterality indices

We computed laterality indices (LIs) of hemispheric dominance for language for both verbal fluency and verb generation fMRI, using the word generation contrast for the former, and the "Generation minus Repetition" contrast for the latter. We used the bootstrap method of the SPM LI toolbox[e9] for all computations, as previously.[60] For demographic purposes (Table 1), we report a composite measure of frontal lobe laterality, based on a bilateral mask derived from merging the three bilateral frontal language ROIs described above. Repeat comparisons of language fMRI activation in patients with FLE and controls used frontal LIs as nuisance regressor in addition to participant age and sex (Supplementary Table 7). For the latter analyses, we computed three distinct frontal LI metrics, each specific to one given bilateral frontal ROI, for both verbal fluency and verb generation fMRI, using the above methods. The latter LI measures are referred to as "ROI-specific" in Supplementary Table 7.

Curves of gradient-based task-effects: group comparisons via functional data analysis

Global differences between curves of gradient-stratified task effects were determined via functional data analysis (FDA).[72] Areas between curves (AbC) were computed by summing the absolute values of group differences between β weights (*y* values) at each gradient bin (*x* values): $AbC= \Sigma_i |y_{group1}(x_i) - y_{group2}(x_i)|$. Statistical significance of intergroup differences was tested using a non-parametric permutation test with 10000 permutations, as previously.[72] The group identity of each participant (e.g., FLE and CTR, for comparison of these two groups) was randomly reassigned without replacement, creating pseudo-groups; average curves for the two pseudo-groups were determined, and the area between these two curves, AbC', was estimated as above. Repeating this process for a number of iterations *I* (in this study, *I*=10000) led to a set of *I* AbC' values; *p*-values for the true group difference were established as the number of AbC' values greater than AbC, divided by the number of iterations *I*.

SUPPLEMENTARY RESULTS

Correlations of cognitive performance and task effects across 17 systems: language fMRI

For verbal fluency, follow-up analyses addressing associations between cognitive performance and task-related effects across 17 systems identified: (i) negative correlations between activity of the frontoparietal control-C subsystem and both letter fluency and naming scores (r_{perm}=-0.22/-0.20, p_{unc}=0.007/0.019); and (ii) a negative correlation between activity across the peripheral visual system and letter fluency scores (r_{perm}=-0.18, p_{unc}=0.029). For verb generation, there were: (i) positive correlations between activity of the DMN-B system and both category fluency and naming scores (r_{perm}=0.16/0.17, p_{unc}=0.048/0.036, respectively); and (ii) a positive correlation between naming scores and task activity encompassing the medial-temporal (limbic A) subsystem (r_{perm}=0.16, p_{unc}=0.049).

Sensitivity analyses

(1) *Analysis across 17 systems* (Supplementary Figures 2 and 3): We probed group differences across DMN and frontoparietal control system subdivisions, using a more fine-grained 17-system parcellation.[35] These analyses confirmed an altered balance of task-related activation and deactivation in FLE versus controls across subsystems, extending our main findings.

(2) *Influence of clinical characteristics on intergroup differences* (Supplementary Table 8): We repeated group comparisons using seizure frequency, history of FBTCS and time since last seizures as nuisance covariates, owing to differences in the latter variables between FLE and TLE. Repeat comparisons recapitulated our main analyses, with near-identical results for verbal working memory and verbal fluency; for verb generation, differences were replicated after co-varying for seizure frequency, while covarying for history of FBTCS and time since last seizure altered peak *p*-values for locations mostly exhibiting task-related deactivation (angular gyrus, posterior DMN).

(3) *Subgroup analysis in FLE with FCD* (Supplementary Figure 4, Supplementary Table 9): We separately investigated an FLE subgroup with suspected focal cortical dysplasia (FLE-FCD; n=13, pathologically confirmed in 8). Across domains and analysis scales, comparisons of the FLE-FCD subgroup to controls produced similar results to those of the main group comparisons.

(4) *Influence of lesional status* (Supplementary Figures 5 and 6, Supplementary Tables 10 and 11): There were no significant differences in cognitive abilities between lesional and non-

lesional FLE, though we note slightly worse performance of lesional FLE on verbal memory tests, and during the 2-Back verbal working memory task. As for imaging data, subgroup analyses of lesional and non-lesional FLE compared to controls overall highlighted patterns of task-related reorganization similar to those of the whole FLE group. Direct comparisons showed: (i) higher activation of lateral temporal cortices, with left-sided emphasis (p_{FWE}<0.05), in lesional compared to non-lesional FLE for language tasks, and (ii) more marked frontal activity reductions in lesional than non-lesional FLE during verbal working memory (p_{FWE}<0.05).

(5) *Mitigation of the effects of frontal language laterality*: Analysis of language laterality indices (LIs) showed weaker left lateralization of frontal hemispheric dominance for language in FLE compared controls (Table 1). Repeat voxel-based analyses covarying for language LI in frontal regions of interest, however, still yielded significant left frontal activation differences between FLE and controls for both verbal fluency and verb generation tasks (p_{FWE}<0.05; Supplementary Table 7).

(6) *Influence of clinical variables on language laterality*: Clinical characteristics may affect language laterality in people with epilepsy.[20] Nonparametric correlation analyses of language LI with age at onset, epilepsy duration, seizure frequency, FBTCS history and time since last seizure ruled out a significant association of clinical characteristics with language LI both in FLE and TLE (all p>0.05, ρ range= |0.01-0.21| and |0.00-0.15| in FLE and TLE).

(7) *Separate analysis of left and right FLE* (Supplementary Figure 7): Lateralization of the seizure focus may differentially affect cognitive system activity. We thus conducted repeat voxel-level analyses separately comparing left and right FLE subgroups to controls. For language tasks, left and right FLE had similar differences compared to controls. Patterns in left and right FLE were also similar for the verbal working memory task. For visual working memory, there were more marked frontoparietal activation and impaired frontal DMN deactivation in left FLE, for the 1-0 Back contrast, and more marked activation decreases in right FLE for the 2-1Back contrast.

Supplementary Figure 1: Comparison of TLE and CTR, voxel-based analyses

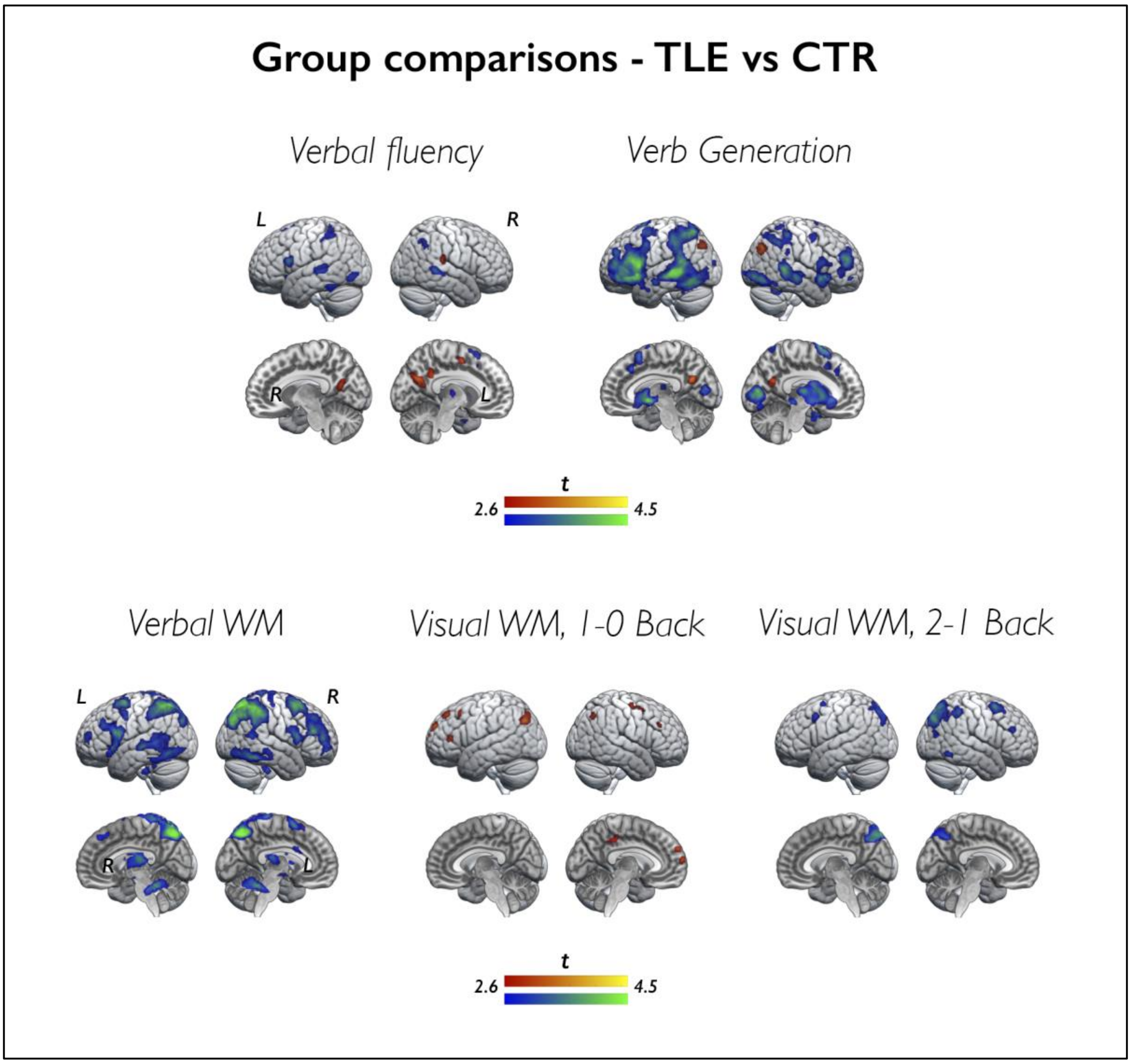

Cold/warm colors indicate lower/higher task-related effects in TLE patients than healthy controls (CTR), respectively. For language tasks, increases in TLE exclusively map onto areas undergoing task-related deactivation (see Figure 2, main text), and signify impaired deactivation in TLE versus controls. Group differences are shown at $p<0.005$, with an extent threshold of 10 voxels applied for display purposes; color bars indicate corresponding t-score scales. MNI152 coordinates and p-values for group comparisons are provided in Supplementary Tables 1-5. Comparisons of TLE and healthy control participants for system and gradient analyses are shown in Figures 3 and 5 (main manuscript).

Supplementary Figure 2: Analyses across 17 systems, Language fMRI

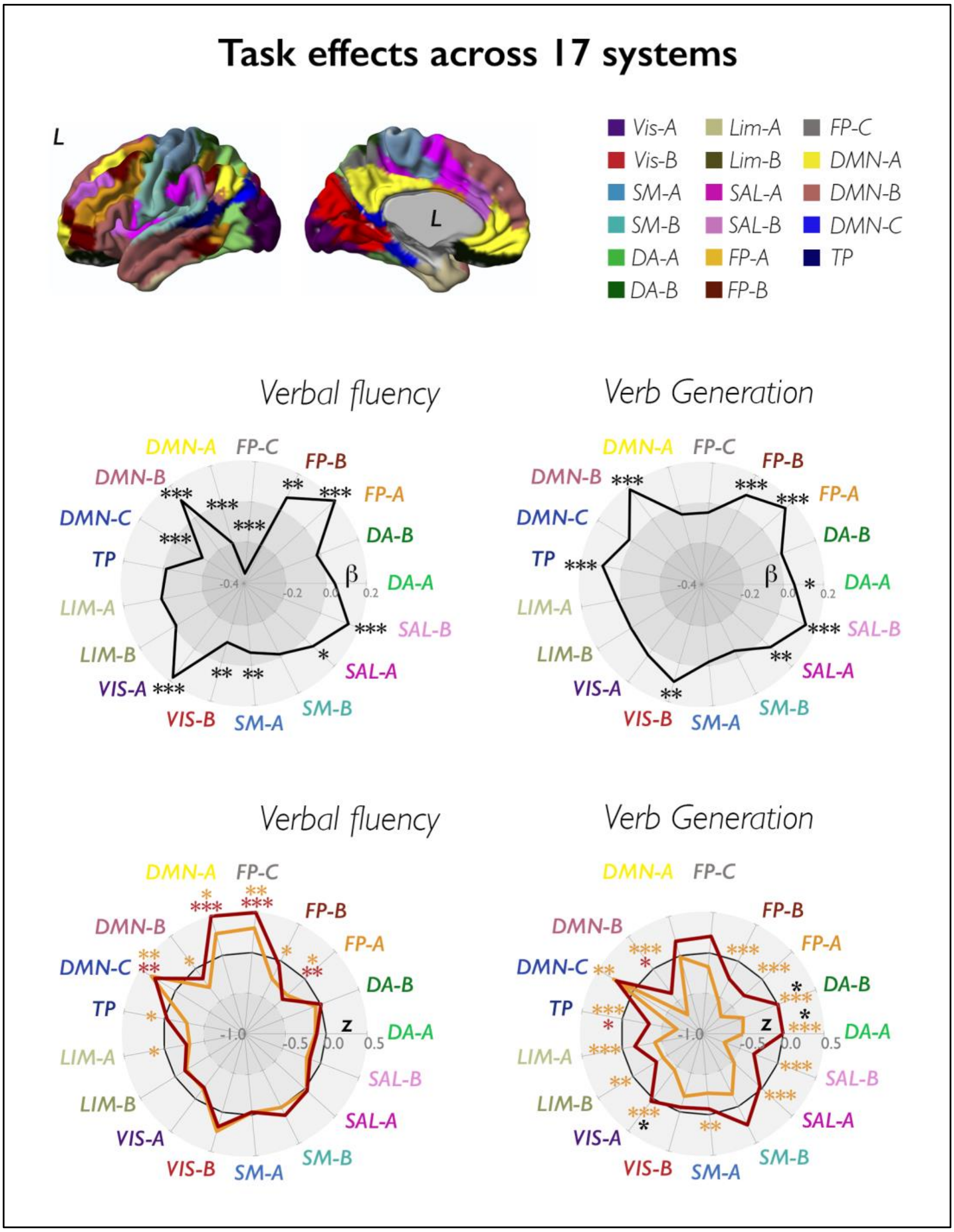

The 17-system Yeo-Krienen[35] parcellation (upper panel) provides refined insights into task effects across executive control and default-mode systems, which are functionally heterogeneous[106] and encompass large patches of cortex in the 7-system partition. Spider plots in the middle panels show mean task-related effects in controls, parameterized as β weights. Spider plots in the lower panels show Z-score analyses of task-related effects across the same systems; across panels, black heptagons display effects in controls (Z-score= 0, at each system), while effects in FLE and TLE are shown in dark red and orange lines and in correspondingly colored asterisks, respectively. Black asterisks highlight differences between FLE and TLE; *** = $p_{FDR}<0.01$; ** = $p_{FDR}<0.05$; * = uncorrected $p<0.05$. For verbal fluency, findings point to an altered balance between system

activation and deactivation both in FLE and TLE, with more prominent effects in the former. For verb generation, there are mild activation reductions in FLE, and, conversely, extensively reduced activation across most cognitive systems in TLE. Differences between patient groups mostly map onto attentional systems. DA: dorsal attention; FP: cognitive (frontoparietal) control; DMN: default-mode network; TP: temporo-parietal; LIM: limbic; VIS: visual; SM: somatomotor; SAL: salience (ventral attention).

Supplementary Figure 3: Analyses across 17 systems, WM fMRI

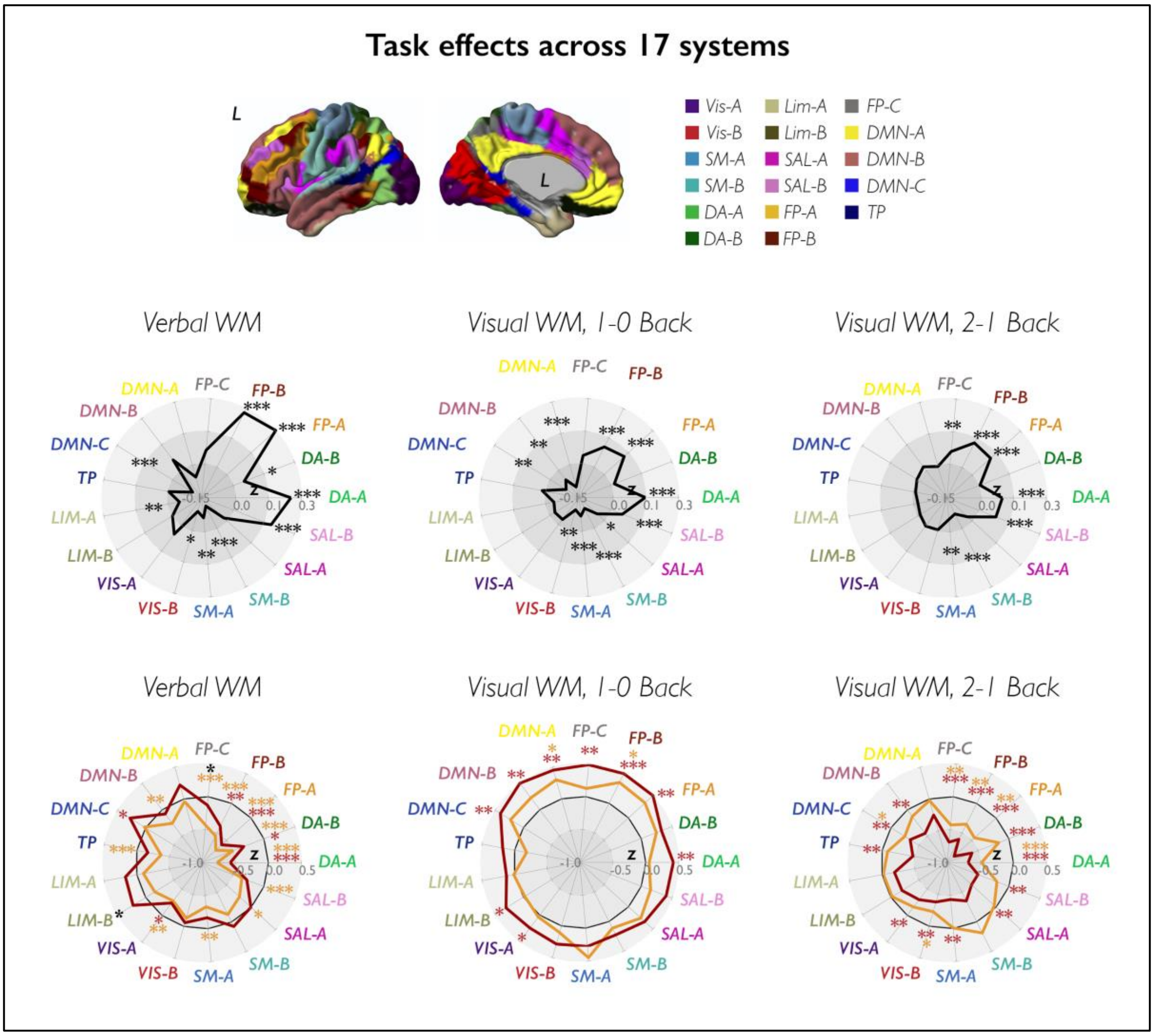

The 17-system Yeo-Krienen[35] parcellation is displayed in the upper panel. The spider plots in the middle panels show mean task-related effects in controls, parameterized as β weights (contrast estimates) across 17 functional systems. The spider plots in the lower panels show Z-score analyses of task-related effects across the same systems; across panels, black heptagons display effects in controls (Z-score= 0, at each system), while effects in FLE and TLE are shown in dark red and orange lines and in correspondingly colored asterisks, respectively. Black asterisks highlight differences between FLE and TLE; *** = $p_{FDR}<0.01$; ** = $p_{FDR}<0.05$; * = uncorrected $p<0.05$. Z-score for control-A/control-B/DMN-B task effects in FLE, 1-0Back visual working memory= 0.52/0.66/0.53, respectively; for display purposes, the spider plot axes reach a maximum of Z=0.5. For verbal working memory, findings point to marked activation reductions across attentional and executive systems both in FLE and TLE, with more marked alterations in TLE. For visual working memory, our results point to enhanced activation of attentional and executive control systems and lesser default-mode deactivation for lower WM task demands, that is followed by defective additional recruitment of attentional and executive control systems for higher task difficulty levels in both patient groups, with more marked deviations in FLE.

Supplementary Figure 4: Comparison of FLE-FCD and CTR, voxel-based analyses

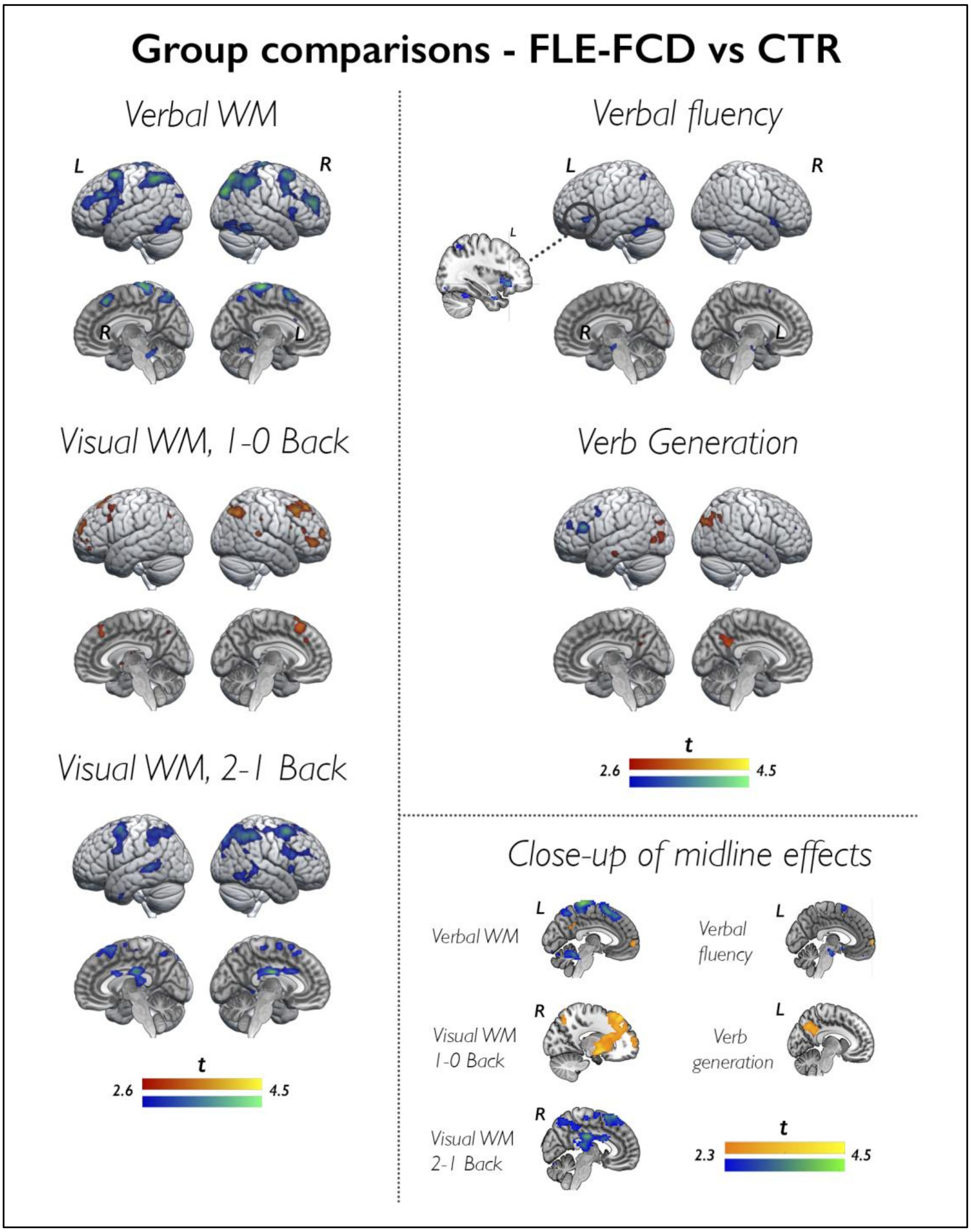

The figure shows voxel-based maps of group differences between patients with FLE-FCD and healthy controls (CTR) in all tasks. Final comparisons included 11/12/11/10 FLE-FCD patients and 49/47/49/50 controls for verbal fluency/verb generation/verbal WM/visual WM, respectively (see above for quality control and participant exclusion criteria). Voxel-based differences are shown for the whole brain at $p<0.005$ with an extent threshold of 10 voxels applied for display purposes. Cold/warm colors indicate lower/higher task-related effects in FLE-FCD patients than controls, respectively; color bars indicate *t*-score scales. MNI152 coordinates and *p*-values for group comparisons within prespecified regions of interest are provided in Supplementary Table 9. Sagittal brain slices in the lower right-hand panel ("Close up of midline effects") further illustrate group differences for effects in midline and para-midline brain areas at a more liberal statistical threshold. WM: working memory.

Supplementary Figure 5. Analysis of lesional and non-lesional FLE patient subgroups

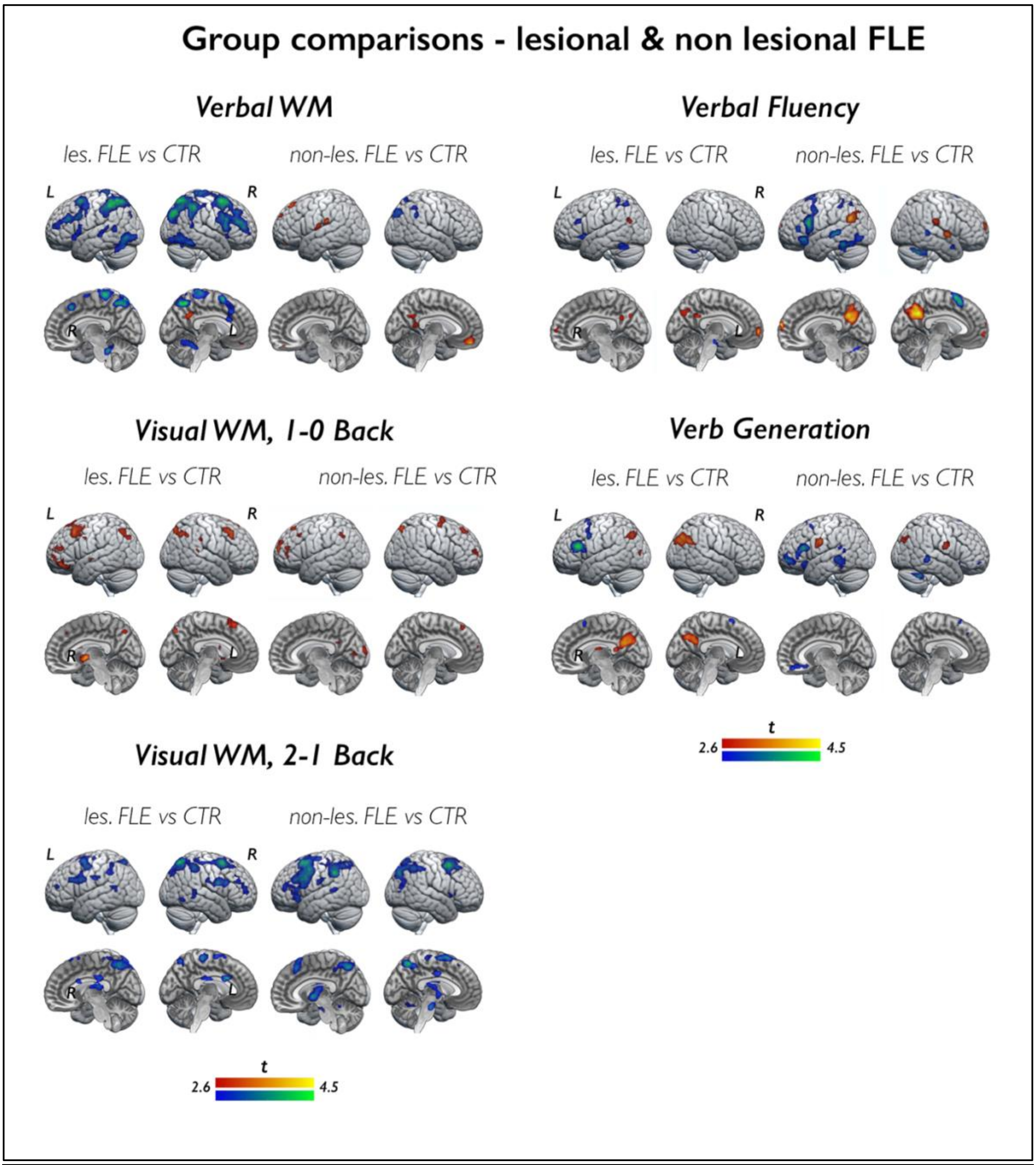

The figure shows voxel-based maps of group differences between patients with lesional FLE and controls (CTR), and between patients with non-lesional FLE and controls in all tasks. Voxel-based differences are shown for the whole brain at $p<0.005$ with an extent threshold of 10 voxels applied for display purposes. Cold/warm colors indicate lower/higher task-related effects in FLE patient subgroups than controls, respectively; color bars indicate *t*-score scales. MNI152 coordinates and *p*-values for group comparisons within prespecified regions of interest are provided in Supplementary Table 10. WM: working memory.

Supplementary Figure 6: Direct comparison of lesional and non-lesional FLE

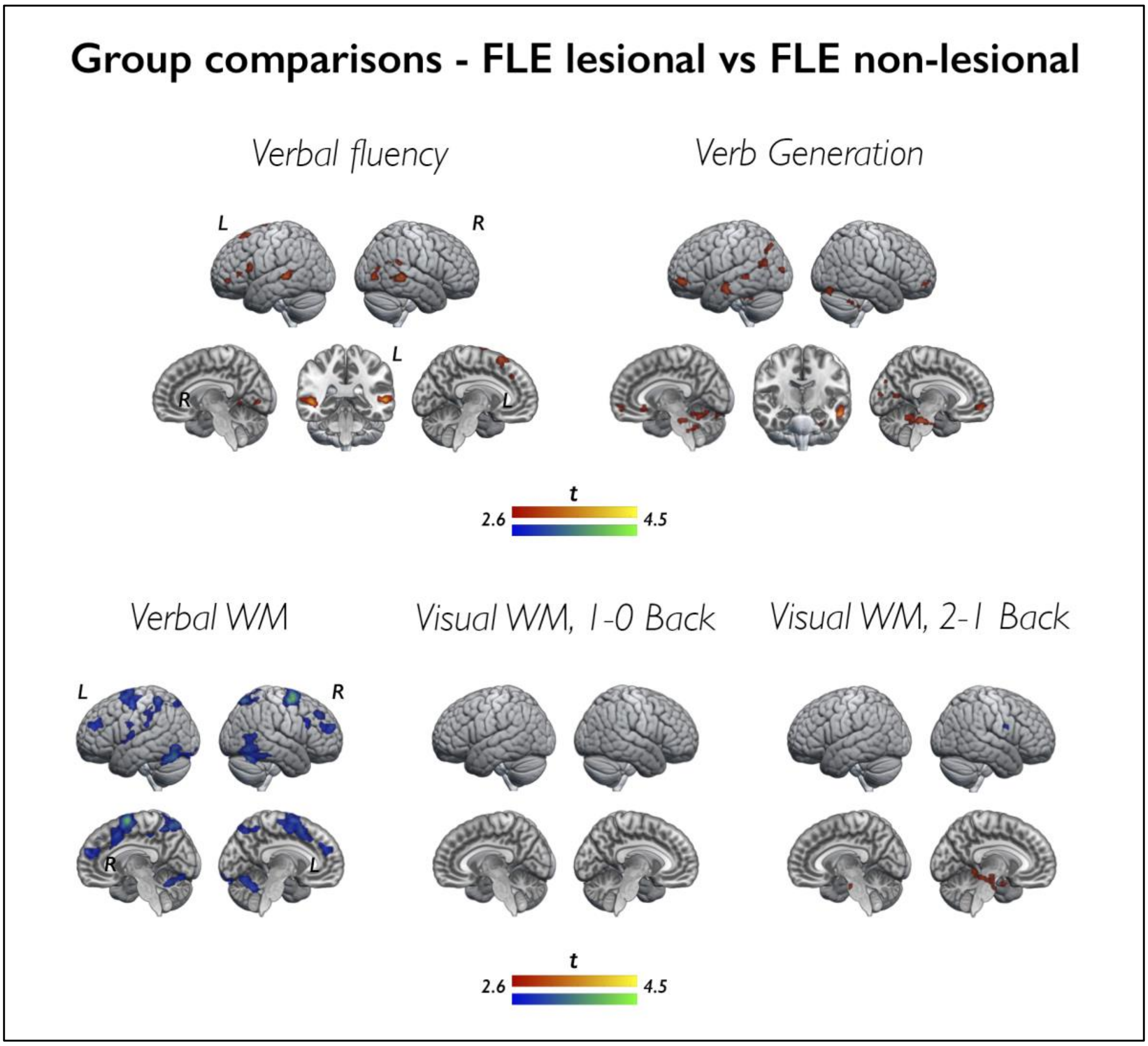

The figure shows voxel-based maps of group differences between patients with FLE and a frontal lobe lesion (FLE lesional) compared to those with no identifiable lesion on MRI (FLE non-lesional) across all the analyzed task contrasts. Cold/warm colors indicate lower/higher task-related effects in FLE-lesional versus non-lesional subgroups. Group differences are shown at $p<0.005$, with an extent threshold of 10 voxels applied for display purposes; color bars indicate corresponding *t*-score scales. MNI152 coordinates and *p*-values for group comparisons within prespecified regions of interest are provided in Supplementary Table 11. WM: working memory.

Supplementary Figure 7: Analysis of left and right FLE subgroups

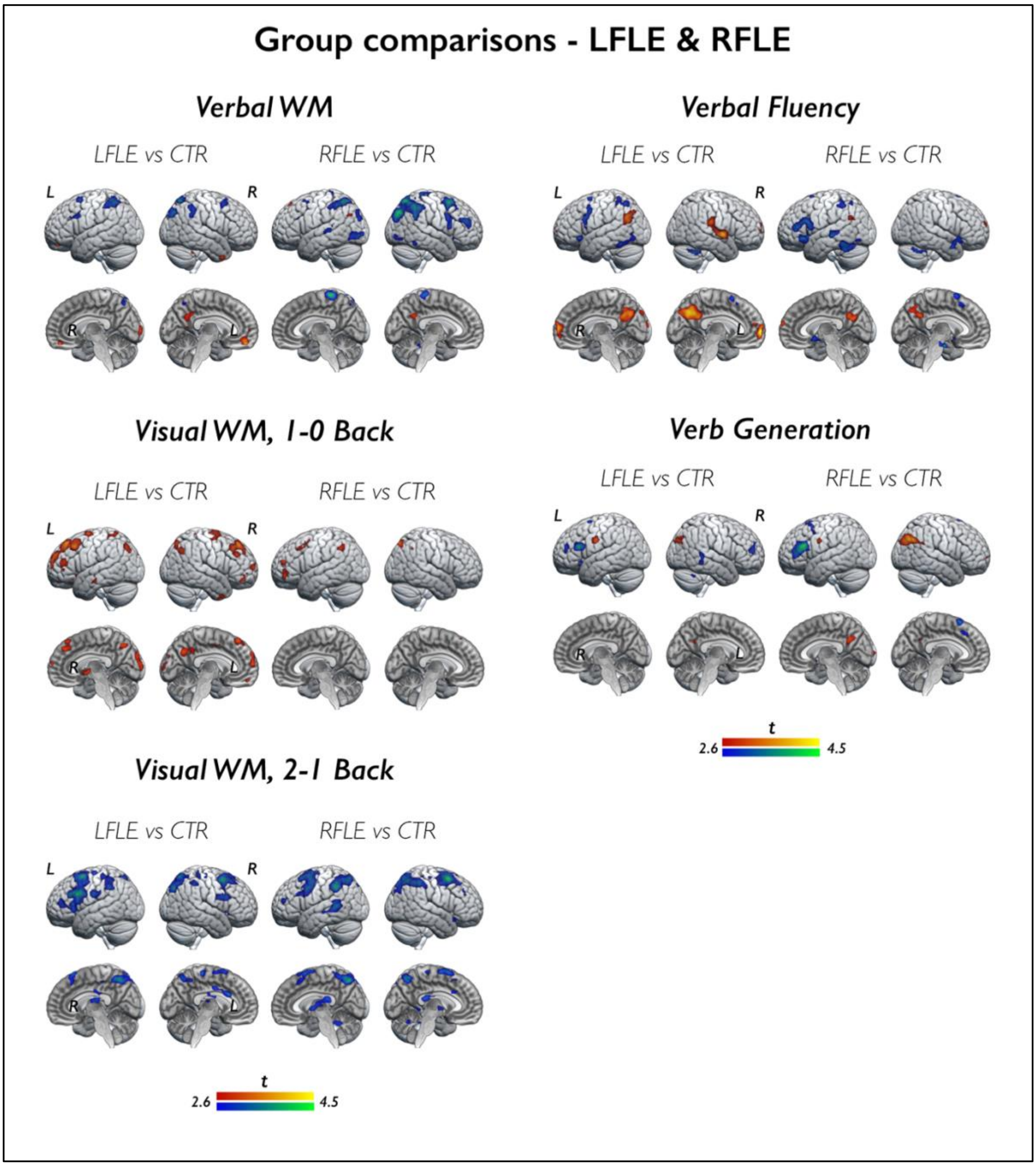

The figure shows voxel-based maps of group differences between patients with left and right FLE and healthy controls across all the analyzed task contrasts. Voxel-based differences are shown for the whole brain at $p<0.005$ with an extent threshold of 10 voxels applied for display purposes. Cold/warm colors indicate lower/higher task-related effects in left FLE or right FLE compared to controls, respectively; color bars indicate *t*-score scales. WM: working memory.

SUPPLEMENTARY TABLES

Supplementary Table 1. Neuropsychological test data: group comparisons

	**CTR (n=52)**	**FLE (n=56)**	**TLE (n=64)**	**Test statistic (F, H or χ^2)**	***P* value**	***Post-hoc tests* (Bonferroni-corrected)**
IQ (NART)<br>[mean (SD)]	112.1 (8.8)	99.7 (11.0)	97.3 (12.3)	24.2	**3.3×10^{-9}**[a]	FLE/CTR: **1.0×10^{-6}**<br>FLE/TLE: 0.58<br>TLE/CTR: **1.5×10^{-9}**
Letter Fluency<br>[mean (SD)]	18.8 (6.1)	10.9 (4.9)	13.2 (6.0)	23.8	**3.5×10^{-9}**[a]	FLE/CTR: **1.3×10^{-9}**<br>FLE/TLE: 0.25<br>TLE/CTR: **3.0×10^{-6}**
Category Fluency<br>[mean (SD)]	24.0 (4.9)	18.2 (5.9)	17.9 (5.1)	19.4	**8.8×10^{-8}**[a]	FLE/CTR: **1.0×10^{-6}**<br>FLE/TLE: 1.00<br>TLE/CTR: **2.3×10^{-7}**
Graded Naming Test<br>[mean (SD)]	22.0 (3.6)	16.7 (4.3)	14.3 (5.7)	42.7	**3.8×10^{-14}**[a]	FLE/CTR: **2.0×10^{-6}**<br>FLE/TLE: **4.4×10^{-4}**<br>TLE/CTR: **7.8×10^{-16}**
Vocabulary<br>[mean (SD)]	–	9.1 (3.7)	8.0 (2.8)	−1.8[b]/−5.7[b]/3.0	–	FLE/norm: 0.23<br>FLE/TLE: 0.26<br>TLE/norm: **1.2×10^{-6}**
Similarities<br>[mean (SD)]	–	8.3 (3.2)	8.5 (2.8)	−3.9[b]/−4.3[b]/0.3	–	FLE/norm: **9.5×10^{-4}**<br>FLE/TLE: 1.00<br>TLE/norm: **1.7×10^{-4}**
Digit Span<br>[mean (SD)]	11.4 (2.6)	8.0 (2.9)	8.0 (2.2)	25.1	**2.0×10^{-9}**[a]	FLE/CTR: **8.2×10^{-9}**<br>FLE/TLE: 1.00<br>TLE/CTR: **8.2×10^{-9}**
Trail Making A[c]<br>[mean (SD)]	28.1 (8.8)	41.4 (17.8)	35.9 (14.7)	10.7	**7.3×10^{-5}**[a]	FLE/CTR: **2.7×10^{-5}**<br>FLE/TLE: 0.13<br>TLE/CTR: **0.022**
Trail Making B-A[c]<br>[mean (SD)]	25.8 (11.2)	69.0 (55.5)	47.4 (36.5)	18.6	**1.7×10^{-7}**[a]	FLE/CTR: **3.2×10^{-8}**<br>FLE/TLE: **0.015**<br>TLE/CTR: **0.002**
List Learning (A1-5)<br>[mean (SD)]	58.1 (8.2)	49.9 (9.6)	43.9 (10.8)	26.0	**1.7×10^{-9}**[a]	FLE/CTR: **1.0×10^{-4}**<br>FLE/TLE: **0.010**<br>TLE/CTR: **8.04×10^{-11}**
List Learning (A6)<br>[mean (SD)]	12.1 (3.0)	10.4 (3.4)	8.4 (3.5)	15.5	**1.5×10^{-6}**[a]	FLE/CTR: 0.10<br>FLE/TLE: **0.002**<br>TLE/CTR: **6.4×10^{-7}**
Design Learning (A1-5)<br>[mean (SD)]	38.8 (5.1)	32.3 (9.6)	31.4 (8.0)	11.7	**3.0×10^{-5}**[a]	FLE/CTR: **1.2×10^{-4}**<br>FLE/TLE: 1.00<br>TLE/CTR: **1.2×10^{-4}**
Design Learning (A6)<br>[mean (SD)]	8.2 (1.4)	6.8 (2.4)	6.4 (2.6)	6.8	**0.002**[a]	FLE/CTR: **0.005**<br>FLE/TLE: 1.00<br>TLE/CTR: **0.004**
Verbal WM task,<br>Verbal Monitoring<br>[median (IQR)]	100.0 (0.0)	100.0 (7.1)	100.0 (7.1)	1.3	0.53[a]	
Verbal WM task,<br>2 Back<br>[median (IQR)]	100.0 (9.6)	92.3 (19.2)	84.6 (28.9)	16.4	**3.6×10^{-4}**[a]	FLE/CTR: **0.014**<br>FLE/TLE: 1.00<br>TLE/CTR: **2.4×10^{-4}**
Visual WM task,<br>0 Back<br>[median (IQR)]	94.7 (7.3)	94.7 (8.7)	90.7 (15.0)	12.1	**0.002**[a]	FLE/CTR: 1.00<br>FLE/TLE: **0.027**<br>TLE/CTR: **0.004**
Visual WM task,<br>1 Back<br>[median (IQR)]	90.0 (20.0)	78.6 (38.6)	72.9 (42.1)	14.8	**7.5×10^{-4}**[a]	FLE/CTR: **0.013**<br>FLE/TLE: 1.00<br>TLE/CTR: **0.001**
Visual WM task,<br>2 Back<br>[median (IQR)]	73.8 (37.7)	43.1 (28.5)	40.0 (44.6)	22.7	**2.1×10^{-5}**[a]	FLE/CTR: **1.8×10^{-4}**<br>FLE/TLE: 1.00<br>TLE/CTR: **5.7×10^{-5}**

Abbreviations: CTR= healthy controls; IQR= interquartile range; NART= National Adult Reading Test; norm= normative ranges (Wechsler scale); WM= working memory. Neuropsychological test data were compared with ANCOVA, using age and sex as covariates, except for digit span, vocabulary, and similarities (see below); working memory task performance data were compared via Kruskal-Wallis tests. Pairwise deletion was applied in case of missing data.

[a] *P* values for comparisons across neuropsychological and task performance measures among groups were FDR-adjusted for multiple comparisons. All *post-hoc* tests were Bonferroni-corrected. As digit span scores were already adjusted for age (as per Wechsler score scales), ANCOVA with sex as the sole covariate was used for these.

[b] For vocabulary and similarities: one-sample *t*-tests assessed deviation of FLE and TLE data from published norms (mean=10), provided by the WAIS (*Supplementary Methods*), while ANCOVA with sex as covariate was used to compare FLE to TLE.

[c] Statistics for Trail Making Test A and B-A were carried out on log-transformed data, but raw data are provided here to ensure comparability with published literature.

Supplementary Table 2. Verbal fluency fMRI

Group comparisons: MNI152 coordinates and test statistics

Region	**MNI coordinates (x y z)**			***T* score**	***P* value (FWE)**	**MNI coordinates (x y z)**			***T* score**	***P* value (FWE)**
	Left hemisphere					***Right hemisphere***				
**FLE < CTR**										
Inferior frontal gyrus, orbitalis	-33	26	-8	4.22	0.0104					
Inferior frontal gyrus	-39	11	25	4.53	0.0048					
	-51	14	13	3.9	0.0344					
Middle frontal gyrus	-51	5	46	4.08	0.0146					
	-39	5	64	3.54	0.0740 (0.0001*)					
Middle-anterior temporal	-63	-28	-5	4.1	0.0104					
Middle-posterior temporal	-63	-28	1	3.93	0.0354					
*Inferior temporal/fusiform gyrus^*	-45	-52	-20	4.74	0.0220^					
*Putamen^*	-18	8	-2	4.77	0.0204^					
*Cerebellum^*						30	-61	-32	5.45	0.0018^
**FLE > CTR**										
Anterior temporal						57	-1	-2	3.97	0.0174
Posterior temporal	-51	-61	28	3.99	0.0172					
Angular	-54	-64	31	3.92	0.0214					
	-54	-70	25	3.75	0.0338					
	-45	-76	40	3.69	0.0392					
Anterior task-negative ROI	-3	65	1	4.12	0.0134	6	68	16	4.00	0.0194
Posterior task-negative ROI	-9	-55	34	4.59	0.0034	0	-55	31	4.63	0.0032
	-3	-67	37	4.58	0.0034					
**TLE < CTR**										
Inferior frontal gyrus	-54	14	16	4.40	0.0076					
Middle-posterior temporal						57	-37	4	3.76	0.0582 (0.0002*)
**TLE > CTR**										
Posterior DMN	-21	-67	22	4.51	0.0028	18	-61	19	3.55	0.0492
**FLE < TLE**										
*Cerebellum^*						30	-58	-32	4.94	0.0116^
**FLE > TLE**										
Middle-posterior temporal (within deactivation map)	-54	-55	40	3.86	0.0418					
Posterior temporal (within deactivation map)	-54	-58	-37	3.59	0.0628 (0.0010*)					
	-45	-58	10	3.54	0.0710 (0.0006*)					
Anterior task-negative ROI						18	47	46	3.59	0.0436

Abbreviations: CTR= controls; FLE= patients with frontal lobe epilepsy; FWE= family-wise error; MNI= Montreal Neurological Institute; ROI= region of interest; TLE= patients with temporal lobe epilepsy. Coordinates of fMRI group differences are provided in MNI space. The table reports statistics of permutation-based 2-tailed *t*-tests with 10000 permutations and age and sex as covariates of no interest; *p*-values are voxel-wise FWE-corrected for

multiple comparisons within prespecified regions of interest (Fig. 1), unless otherwise stated. All the reported *p*-values refer to 2-tailed statistical significance. ^Area outside of prespecified ROIs, with voxel-wise statistic surviving 2-tailed $p_{FWE}<0.05$ corrected across the whole brain. *$P<0.001$, uncorrected for multiple comparisons.

Supplementary Table 3. Verb generation fMRI

Group comparisons: MNI152 coordinates and test statistics

Region	**MNI coordinates (x y z)**			***T* score**	***P* value (FWE)**	**MNI coordinates (x y z)**			***T* score**	***P* value (FWE)**
	Left hemisphere					***Right hemisphere***				
**FLE < CTR**										
Inferior frontal gyrus	-45	26	19	5.41	0.0002					
Middle frontal gyrus	-45	5	43	3.55	0.0486					
**FLE > CTR**										
Angular						36	-85	34	4.59	0.0024
**TLE < CTR**										
Inferior frontal gyrus	-51	17	13	5.55	0.0004	54	11	10	3.50	0.0966 (0.0006*)
	-42	2	37	3.64	0.0698 (0.0002*)					
Middle frontal gyrus	-42	5	58	3.62	0.0730 (0.0006*)	30	-1	49	3.58	0.0766 (0.0004*)
Inferior frontal gyrus, orbitalis	-36	23	7	4.68	0.0006					
	-45	17	-8	4.64	0.0008					
	-33	32	-2	4.60	0.0010					
Anterior temporal	-57	5	14	3.47	0.0696 (0.0010*)	48	11	-14	3.88	0.0206
	-45	8	-5	3.40	0.0834 (0.0010*)					
Middle-anterior temporal	-66	-25	-8	4.57	0.0028	51	-25	-11	4.57	0.0028
	-54	-10	-17	3.77	0.0304					
Middle-posterior temporal	-54	-40	-5	6.17	0.0002	54	-40	4	4.65	0.0024
	-60	-49	13	4.45	0.0066	51	-28	-8	4.50	0.0056
	-60	-46	25	4.00	0.0232					
	-51	-40	40	4.15	0.0160					
Posterior temporal	-63	-58	13	4.19	0.0122	48	-79	-5	3.68	0.0456
	-51	-76	-5	3.70	0.0438					
	-54	-70	-8	3.66	0.0484					
Angular	-27	-64	37	4.08	0.0126					
*Superior parietal lobule^*	-33	-64	58	4.99	0.0112^					
*Hippocampus^*	-27	23	-11	4.82	0.0192^					
	-33	32	2	4.60	0.0424^					
*Globus pallidus^*						9	2	-8	4.74	0.0250^
**TLE > CTR**										
Angular	-42	-79	40	3.96	0.0184	45	-76	37	4.59	0.0022
Posterior task-negative	-15	-64	22	3.50	0.0714 (0.0008*)	12	-61	22	3.65	0.0458
**FLE < TLE**										
No significant differences										
**FLE > TLE**										
Posterior temporal	-48	-79	-5	3.84	0.0240					
Angular	-21	-79	34	3.85	0.0238	30	-76	28	4.00	0.0136
	-27	-94	19	3.73	0.0346	24	-73	37	3.52	0.0590 (0.0004*)
Posterior task negative ROI	-18	-79	37	3.88	0.0148	21	-76	37	3.65	0.0338
*Occipital pole^*						0	-100	4	4.99	0.0092^
*Lateral occipital cortex^*						39	-91	-2	4.73	0.0212^

Abbreviations: CTR= controls; FLE= patients with frontal lobe epilepsy; FWE= family-wise error; MNI= Montreal Neurological Institute; ROI= region of interest; TLE= patients with temporal lobe epilepsy. Coordinates of fMRI group

differences are provided in MNI space. The table reports statistics of permutation-based 2-tailed *t*-tests with 10000 permutations and age and sex as covariates of no interest; *p*-values are voxel-wise FWE-corrected for multiple comparisons within prespecified regions of interest (Fig. 1), unless otherwise stated. All the reported *p*-values refer to 2-tailed statistical significance. ^Area outside of prespecified ROIs, surviving 2-tailed $p_{FWE} < 0.05$, voxel-wise corrected across the whole brain. *$P<0.001$, uncorrected for multiple comparisons.

Supplementary Table 4. Verbal Working Memory (WM) fMRI

Group comparisons: MNI152 coordinates and test statistics

Region	**MNI coordinates (x y z)**			***T* score**	***P* value (FWE)**	**MNI coordinates (x y z)**			***T*-score**	***P* value (FWE)**
	Left hemisphere					***Right hemisphere***				
**FLE < CTR**										
FEF/premotor ROI (BA 6-8)	-27	8	58	3.80	0.0392	27	11	55	5.00	0.0008
Lateral prefrontal ROI (BA 9/46)	-39	8	37	3.65	0.0524 (0.0004*)	36	11	49	4.00	0.0176
						48	38	25	3.59	0.0612 (0.001*)
Dorsal Parietal ROI	-33	-58	55	5.26	0.0006	18	-67	61	5.56	0.0004
	-15	-70	55	4.07	0.0248	30	-79	37	5.34	0.0004
	-45	-49	58	4.03	0.0274	42	-46	46	4.98	0.0012
						51	-46	34	4.38	0.0088
Posterior task-negative ROI[#] (within activation map)						24	-76	34	4.55	0.0014
**FLE > CTR**										
Anterior task-negative ROI	0	47	-20	4.25	0.0046					
Posterior task-negative ROI	-9	-55	31	3.96	0.014					
**TLE < CTR**										
FEF/premotor ROI (BA 6-8)	-33	2	67	4.53	0.0052	27	17	58	4.89	0.0016
	-27	11	58	3.95	0.0276	27	5	70	4.15	0.0154
	-6	8	64	3.63	0.0664 (0.0006*)	48	35	28	3.69	0.0584 (0.0006*)
Lateral prefrontal ROI (BA 9/46)	-54	11	19	4.15	0.0128	45	41	22	4.69	0.0018
	-48	32	38	3.57	0.0718 (0.0004)	36	11	49	3.93	0.0230
	-48	8	16	3.46	0.0954 (0.0010*)	33	53	10	3.91	0.0244
						27	23	52	3.85	0.0312
						30	41	43	3.74	0.0430
Dorsal Parietal ROI	-27	-61	49	6.10	0.0002	21	-67	61	6.28	0.0002
	-6	-67	52	5.36	0.0004	42	-46	40	5.98	0.0002
	-45	-55	58	4.82	0.0014	30	-79	37	5.91	0.0002
	-24	-82	37	4.15	0.0186					
Posterior task-negative ROI[#] (within activation map)	-6	-76	46	3.63	0.0288	9	-76	46	4.28	0.0032
	-12	-67	43	3.50	0.0446	24	-76	34	3.94	0.0114
						3	-55	49	3.60	0.0318
						18	-73	43	3.43	0.0514 (0.0010*)
*Inferior temporal gyrus^*						57	-49	-20	4.75	0.0136^
*Superior occipital gyrus^*	-27	-82	34	4.44	0.0434^					
**TLE > CTR**										
No significant differences										
**FLE < TLE**										
No significant differences										
**FLE > TLE**										
Posterior task-negative ROI (within deactivation map)	-3	-49	13	3.51	0.0486	6	-46	13	4.27	0.0062
	-6	-67	37	3.43	0.0584 (0.0010*)					

Abbreviations: BA= Brodmann Area; CTR= controls; FEF= frontal eye field; FLE= patients with frontal lobe epilepsy; FWE= family-wise error; MNI= Montreal Neurological Institute; ROI= region of interest; TLE= patients with temporal lobe epilepsy. Coordinates of fMRI group differences are provided in MNI space. The table reports statistics of permutation-based 2-tailed *t*-tests with 10000 permutations and age and sex as covariates of no interest; *p*-values are voxel-wise FWE-corrected for multiple comparisons within prespecified regions of interest (Fig. 1), unless otherwise stated. All *p*-values refer to 2-tailed statistical significance. ^ Area outside of prespecified ROIs, with voxel-based statistic surviving 2-tailed p_{FWE} <0.05, corrected across the whole brain. *P<0.001, uncorrected for multiple comparisons. [#] A small portion of the dorsal precuneus, which formally belongs to the posterior task-negative ROI, displays task-related activation during WM.

Supplementary Table 5. Visual Working Memory (WM) fMRI, 1-0 Back

Group comparisons: MNI152 coordinates and test statistics

Region	**MNI coordinates (x y z)**			***T* score**	***P* value (FWE)**	**MNI coordinates (x y z)**			***T* score**	***P* value (FWE)**
	Left hemisphere					***Right hemisphere***				
**FLE < CTR**										
No significant differences										
**FLE > CTR**										
FEF/premotor ROI (BA 6-8)	-45	23	43	3.76	0.0444					
Lateral prefrontal ROI (BA 9/46)	-48	20	43	3.85	0.0388					
Dorsal Parietal ROI						24	-76	55	4.11	0.0242
						33	-76	52	3.66	0.0858 (0.0006*)
Anterior task-negative ROI	-18	62	13	3.62	0.0454					
**TLE < CTR**										
No significant differences										
**TLE > CTR**										
Anterior task-negative ROI	-12	59	28	3.63	0.0364					
	-6	68	13	3.60	0.0400					
	-18	41	46	3.34	0.0836 (0.0010*)					
**FLE < TLE**										
No significant differences										
**FLE > TLE**										
No significant differences										

Abbreviations: BA= Brodmann Area; CTR= controls; FEF= frontal eye field; FLE= patients with frontal lobe epilepsy; FWE= family-wise error; MNI= Montreal Neurological Institute; ROI= region of interest; TLE= patients with temporal lobe epilepsy. Coordinates of fMRI group differences are provided in MNI space. The table reports statistics of permutation-based 2-tailed *t*-tests with 10000 permutations and age and sex as covariates of no interest; the associated *p*-values are voxel-wise FWE-corrected for multiple comparisons within pre-defined regions of interest (Fig. 1), unless otherwise stated. All *p*-values refer to 2-tailed statistical significance. *P<0.001, uncorrected for multiple comparisons.

Supplementary Table 6. Visual Working Memory (WM) fMRI, 2-1 Back

Group comparisons: MNI152 coordinates and test statistics

Region	**MNI coordinates (x y z)**			***T* score**	***P* value (FWE)**	**MNI coordinates (x y z)**			***T* score**	***P* value (FWE)**
	Left hemisphere					***Right hemisphere***				
**FLE < CTR**										
FEF/premotor ROI (BA 6-8)	-24	8	58	4.37	0.0052	30	11	58	5.23	0.0010

	-39	5	61	4.28	0.0068	6	26	64	3.62	0.0454
	-12	-1	67	4.02	0.0136	0	26	49	3.44	0.0702 (0.001*)
Lateral prefrontal ROI (BA 9/46)	-39	14	25	4.64	0.0018	27	23	52	3.99	0.0132
	-42	2	46	3.74	0.0290	39	8	52	3.83	0.0232
	-45	50	10	3.30	0.0884 (0.0008*)	36	5	31	3.63	0.0384
						27	17	46	3.52	0.0536 (0.0008*)
Dorsal Parietal ROI	-15	-70	58	4.65	0.0034	12	-67	55	4.79	0.0022
	-51	-46	40	4.40	0.0066	18	-67	64	4.73	0.0024
	-36	-49	37	3.95	0.0260	51	-43	49	4.00	0.0218
	-24	-61	43	3.91	0.0288	36	-49	37	3.77	0.0450
Posterior task-negative ROI# (within activation map)						3	-61	46	3.61	0.0464
**FLE > CTR**										
No significant differences										
**TLE < CTR**										
FEF/premotor ROI (BA 6-8)						30	11	58	3.99	0.0142
Lateral prefrontal ROI (BA 9/46)	-45	14	43	3.30	0.0878 (0.0008*)					
Dorsal Parietal ROI	-21	-79	-52	3.56	0.0800 (0.0002*)	30	-82	43	4.37	0.0076
						12	-76	58	4.28	0.0096
						18	-64	58	3.97	0.0232
						27	-70	49	3.74	0.0494
						45	-43	49	3.64	0.0638 (0.0006*)
Posterior task-negative ROI# (within activation map)						3	-64	43	3.67	0.0338
						9	-79	46	3.58	0.0424
**TLE > CTR**										
No significant differences										
**FLE < TLE**										
No significant differences										
**FLE > TLE**										
No significant differences										

Abbreviations: BA= Brodmann Area; CTR= controls; FEF= frontal eye field; FLE= patients with frontal lobe epilepsy; FWE= family-wise error; MNI= Montreal Neurological Institute; ROI= region of interest; TLE= patients with temporal lobe epilepsy. Coordinates of fMRI group differences are provided in MNI space. The table reports statistics of permutation-based 2-tailed *t*-tests with 10000 permutations and age and sex as covariates of no interest; the associated *p*-values are voxel-wise FWE-corrected for multiple comparisons within pre-defined regions of interest (Fig. 1), unless otherwise stated. All the reported *p*-values refer to 2-tailed statistics. *A small portion of the dorsal precuneus, formally belonging to the posterior task-negative ROI, displays task-related activation during WM. *P<0.001, uncorrected for multiple comparisons.

Supplementary Table 7. FLE versus controls: Language fMRI, frontal group differences covaried for language LI

Group comparisons, frontal lobe ROIs: MNI152 coordinates and test statistics

FRONTAL ROI	**MNI coordinates (x y z)**			***T* score**	***P* value (FWE)**	**MNI coordinates (x y z)**			***T* score**	***P* value (FWE)**
	Left hemisphere					***Right hemisphere***				
**FLE < CTR, *Verbal fluency fMRI***										
Inferior frontal gyrus (covaried for ROI-specific LI)	-39	11	25	3.86	0.0404					

Inferior frontal gyrus, orbitalis (covaried for ROI-specific LI)	-30	26	-8	4.29	0.0078	36	17	-5	3.93	0.0240
Middle frontal gyrus (covaried for ROI-specific LI)	-48	5	43	3.82	0.0348					
**FLE < CTR, *Verb Generation fMRI***										
Inferior frontal gyrus (covaried for ROI-specific LI)	-45	23	19	4.46	0.0050					

Abbreviations: CTR= controls; FLE= patients with frontal lobe epilepsy; FWE= family-wise error; LI= laterality index; MNI= Montreal Neurological Institute; ROI= region of interest. Coordinates of fMRI group differences are provided in MNI space. Repeat group comparisons focused on differences between FLE and controls in frontal lobe areas. We used LI measures specific to each of the three frontal lobe ROIs, *i.e.*, comparisons of orbital inferior frontal activation used task-specific LI measures computed using the orbital inferior frontal ROI, and so forth. All group comparisons were conducted via 2-tailed *t*-tests with 10000 permutations, using ROI-specific LI, age, and sex as covariates of no interest; the associated *p*-values are voxel-wise FWE-corrected for multiple comparisons within each region of interest. All the reported *p*-values refer to 2-tailed statistical significance.

Supplementary Table 8. Language and Working Memory fMRI: group differences covarying for seizure frequency, FBTCS history, and time since last seizure

Group comparisons, MNI152 coordinates and test statistics

Region	**MNI coordinates (x y z)**			***T* score**	***P* value (FWE)**	**MNI coordinates (x y z)**			***T* score**	***P* value (FWE)**
	Left hemisphere					***Right hemisphere***				
**FLE < TLE, *Verbal fluency fMRI***										
Cerebellum^ (covaried ***for seizure frequency****)*						30	-58	-32	4.69	0.0288^
Cerebellum^ (covaried for ***FBTCS history****)*						30	-58	-32	4.84	0.0210^
Cerebellum^ (covaried for ***time since last seizure****)*						30	-58	-32	5.13	0.0106^
**FLE > TLE, *Verbal fluency fMRI***										
Middle-posterior temporal (covaried for ***seizure frequency****)*	-54	-55	40	3.54	0.108 (0.0004*)					
Middle-posterior temporal (covaried for ***FBTCS history****)*	-54	-55	40	3.86	0.0494					
Middle-posterior temporal (covaried for ***time since last seizure****)*	-57	-52	40	3.93	0.0370	60	-55	34	3.92	0.0390
Anterior task-negative ROI (covaried for ***seizure frequency****)*	-54	-55	40	3.54	0.1080 (0.0004*)	18	47	46	3.19	0.1326 (0.0018*)
Anterior task-negative ROI (covaried for ***FBTCS history****)*						18	47	46	3.82	0.0256
Anterior task-negative ROI (covaried for ***time since last seizure****)*						18	47	46	4.02	0.0144
**FLE >TLE, *Verb Generation fMRI***										
Posterior temporal (covaried for ***seizure frequency****)*	-48	-79	-5	4.26	0.0050					
Posterior temporal (covaried for ***FBTCS history****)*	-51	-79	-2	3.28	0.1146 (0.0020*)					
Posterior temporal (covaried for ***time since last seizure****)*	-48	-79	-5	3.61	0.0484					
Angular (covaried for ***seizure frequency****)*	-21	-79	34	4.02	0.0136	33	-79	28	3.93	0.0178
	-24	-91	19	3.41	0.0712 (0.0014*)	27	-73	31	3.74	0.0304
Angular (covaried for ***FBTCS history****)*	-21	-79	34	3.39	0.0814 (0.0012*)	30	-76	28	3.71	0.0362
	-27	-94	19	3.12	0.1576 (0.0018*)	24	-73	37	3.17	0.1400 (0.0018*)

Angular (*covaried for* ***time since last seizure***)	-24	-79	31	3.08	0.1680 (0.0026*)	33	-79	28	3.29	0.1034 (0.0022*)
	-27	-94	19	2.81	0.3038 (0.0064*)	24	-73	37	2.93	0.2398 (0.0044*)
Posterior task negative ROI (*covaried for* ***seizure frequency***)	-18	-79	37	4.00	0.0130	21	-76	37	3.46	0.0692 (0.0014*)
Posterior task negative ROI (*covaried for* ***FBTCS history***)	-18	-79	37	3.40	0.0712 (0.0060*)	21	-73	40	3.22	0.1098 (0.0014*)
Posterior task negative ROI (*covaried for* ***time since last seizure***)	-18	-79	37	3.04	0.2082 (0.0030*)	21	-73	40	2.82	0.3148 (0.0074*)
*Occipital pole^* (*covaried for* ***seizure frequency***)						6	-103	7	4.86	0.0166^
*Occipital pole^* (*covaried for* ***FBTCS history***)						6	-103	4	4.61	0.0342^
*Occipital pole^* (*covaried for* ***time since last seizure***)						0	-100	1	4.18	0.1284^ (0.0002*)
*Lateral occipital cortex^* (*covaried for* ***seizure frequency***)						39	-91	-2	4.91	0.0132^
*Lateral occipital cortex^* (*covaried for* ***FBTCS history***)						39	-91	-2	4.64	0.0330^
*Lateral occipital cortex^* (*covaried for* ***time since last seizure***)						42	-88	-5	4.48	0.0582^ (0.0001*)
**FLE >TLE, *Verbal WM fMRI***										
Posterior task-negative ROI (*covaried for* ***seizure frequency***)	-3	-49	13	3.59	0.0332	6	-46	13	4.42	0.0036
	-9	-67	37	3.63	0.0304					
Posterior task-negative ROI (*covaried for* ***FBTCS history***)	-3	-49	13	3.53	0.0422	6	-46	13	4.50	0.002
Posterior task-negative ROI (*covaried for* ***time since last seizure***)	-3	-49	13	3.54	0.0426	6	-46	13	3.99	0.0116

Abbreviations: FBTCS=focal-to-bilateral tonic-clonic seizure; FLE= patients with frontal lobe epilepsy; FWE= family-wise error; MNI= Montreal Neurological Institute; ROI= region of interest; TLE= patients with temporal lobe epilepsy. WM= working memory. Coordinates of fMRI group differences are provided in MNI space. The table reports statistics of permutation-based 2-tailed *t*-tests, using 10000 permutations and age and sex as covariates of no interest, along with (i) seizure frequency (log), or (ii) history of FBTCS, or (iii) time since last seizure; *p*-values are voxel-wise FWE-corrected for multiple comparisons within prespecified ROIs (Fig. 1), unless otherwise stated. All *p*-values refer to 2-tailed statistical significance. ^Area outside of prespecified ROIs, with voxel-based statistic surviving two-tailed $p_{FWE}<0.05$ corrected across the whole brain. *$P<0.001$, uncorrected for multiple comparisons.

Supplementary Table 9. Language and Working Memory fMRI: comparison of FLE-FCD patients and healthy controls

Group comparisons, MNI152 coordinates and test statistics

Region	**MNI coordinates (x y z)**			***T* score**	***P* value (FWE)**	**MNI coordinates (x y z)**			***T* score**	***P* value (FWE)**
	Left hemisphere					***Right hemisphere***				
FLE-FCD < CTR, Verbal fluency fMRI										
Inferior frontal, orbital	-36	26	-8	4.37	0.0498					
FLE-FCD > CTR, Verbal fluency fMRI										
*No significant differences^*										
FLE-FCD < CTR, Verb Generation fMRI										
Inferior frontal gyrus	-48	29	22	4.61	0.0042					
Middle frontal gyrus	-45	5	43	3.31	0.0930 (0.0008*)					

FLE-FCD > CTR, Verb Generation fMRI										
Angular						33	-88	31	4.22	0.0172
FLE-FCD < CTR, Verbal WM fMRI										
FEF/premotor ROI (BA 6-8)	-30	8	61	4.99	0.0080	27	14	55	4.33	0.0078
	-36	29	31	4.31	0.0082	30	8	64	3.99	0.0252
	-6	11	61	3.86	0.0346	3	26	52	4.04	0.0208
Lateral prefrontal ROI (BA 9/46)	-39	29	28	4.81	0.0010	39	50	19	4.58	0.0028
	-42	8	28	4.40	0.0038	45	11	37	3.65	0.0402
						36	8	46	3.62	0.0440
Dorsal Parietal ROI	-33	-58	55	5.08	0.0010	15	-76	58	4.94	0.0014
	-45	-49	55	4.71	0.0032	27	-85	34	4.66	0.0038
	-15	-73	52	4.07	0.0254	51	-40	55	4.30	0.0134
Posterior task-negative ROI (within activation map#)						24	-76	34	3.59	0.0594 (0.0002*)
FLE-FCD > CTR, Verbal WM fMRI										
*No significant differences^*										
FLE-FCD < CTR, Visual WM, 1-0 Back										
No significant differences										
FLE-FCD > CTR, Visual WM, 1-0 Back										
FEF/premotor ROI (BA 6-8)	-12	29	55	3.74	0.1252 (0.0010*)	39	26	49	4.37	0.0256
						21	38	52	3.91	0.0860 (0.0004*)
Lateral prefrontal ROI (BA 9/46)	-21	65	16	3.57	0.1670 (0.0006*)	27	65	13	4.04	0.0678 (0.0006*)
Dorsal Parietal ROI						48	-67	43	4.20	0.0488
						36	-76	46	4.19	0.0500
						36	-52	37	3.97	0.0840 (0.0006*)
Anterior task-negative ROI	-15	65	22	3.71	0.0620 (0.0004*)	18	38	52	4.28	0.0154
	-12	35	52	3.39	0.1166 (0.0010*)					
FLE-FCD < CTR, Visual WM, 2-1 Back										
FEF/premotor ROI (BA 6-8)	-36	2	61	3.9	0.0466	30	11	58	4.23	0.0248
						24	5	58	4.08	0.0330
Dorsal Parietal ROI						30	-61	46	4.68	0.0108
						54	-40	55	4.19	0.0280
						45	-52	52	4.06	0.0390
FLE-FCD > CTR, Visual WM, 2-1 Back										
No significant differences										

Abbreviations: BA= Brodmann Area; CTR= controls; FCD= focal cortical dysplasia; FEF= frontal eye field; FLE= patients with frontal lobe epilepsy; FWE= family-wise error; MNI= Montreal Neurological Institute; ROI= region of interest. Final group comparisons included: 11/12/11/10 FLE-FCD patients for verbal fluency/verb generation/verbal working memory/visual working memory. Coordinates of fMRI group differences are provided in MNI space. The table reports statistics of permutation-based 2-tailed *t*-tests with 10000 permutations and age and sex as covariates of no interest; the associated *p*-values are voxel-wise FWE-corrected for multiple comparisons within pre-defined regions of interest (Fig. 1), unless otherwise stated. All the reported *p*-values refer to 2-tailed statistical significance. ^Lesser deactivation of the anterior task-negative ROI in FLE-FCD than controls evident at $p<0.005$ uncorrected during verbal fluency (x=-3, y=65, z=1, $t=3.39$, $p_{FWE}=0.137$, $p_{unc}=0.0014$), and at $p<0.01$ during verbal working memory (x=-6, y=59, z=1, $t=2.89$, $p_{FWE}=0.289$, $p_{unc}=0.0062$). #A small portion of the dorsal precuneus, formally belonging to the posterior task-negative ROI, displays task-related activation during WM. *$P<0.001$, uncorrected for multiple comparisons.

Supplementary Table 10. Language and Working Memory fMRI: separate comparisons of lesional and non-lesional FLE patients versus controls

Group comparisons, MNI152 coordinates and test statistics

Region	**MNI coordinates (x y z)**			***T* score**	***P* value (FWE)**	**MNI coordinates (x y z)**			***T* score**	***P* value (FWE)**
	Left hemisphere					***Right hemisphere***				
FLE lesional < CTR, Verbal fluency fMRI										
Inferior frontal gyrus	-42	26	22	3.23	0.2084 (0.0008*)					
Inferior frontal gyrus, orbitalis	-36	17	-5	3.24	0.1614 (0.0010*)					
	-33	26	-5	3.21	0.1718 (0.0008*)					
FLE lesional > CTR, Verbal fluency fMRI										
Anterior task-negative ROI	-3	65	1	4.43	0.0052					
Posterior task-negative ROI						15	-58	13	3.46	0.0576 (0.0006*)
FLE non-lesional < CTR, Verbal fluency fMRI										
Inferior frontal gyrus	-51	17	19	4.81	0.0024					
	-42	14	25	4.63	0.0036					
Inferior frontal gyrus, orbitalis	-33	26	-8	4.05	0.0180					
	-48	35	7	3.80	0.0330					
Middle frontal gyrus	-45	5	43	3.69	0.0458					
	-39	8	64	3.49	0.0732 (0.0010*)					
Middle-anterior temporal	-63	-28	-5	3.82	0.0256					
Middle-posterior temporal	-57	-37	1	4.21	0.0174	51	-37	1	3.90	0.0452
	-57	-49	-17	3.57	0.0544 (0.0008*)					
FLE non-lesional > CTR, Verbal fluency fMRI										
Anterior temporal						60	-1	-2	3.88	0.0284
Posterior temporal	-51	-61	28	4.51	0.0034					
Angular	-51	-64	31	4.32	0.0072					
Anterior task-negative ROI						6	68	16	4.88	0.0008
Posterior task-negative ROI	-3	-58	34	4.74	0.0022	18	-61	25	3.56	0.0536 (0.0010*)
FLE lesional < CTR, Verb Generation fMRI										
Inferior frontal gyrus	-48	26	22	4.96	0.0010					
Middle frontal gyrus	-45	5	43	3.50	0.0640 (0.0004*)					
FLE lesional > CTR, Verb Generation fMRI										
Angular	-42	-73	40	3.36	0.0952 (0.0010*)	42	-73	37	4.08	0.0138
Posterior temporal						45	-61	31	3.67	0.0618 (0.0006*)
Anterior task-negative ROI						36	-85	31	3.98	0.0186
						48	-64	31	3.85	0.0260
						0	-55	28	3.97	0.0228
						3	-61	37	3.65	0.0484
FLE non-lesional < CTR, Verb Generation fMRI										
Inferior frontal gyrus	-45	26	16	4.19	0.0074					

Inferior frontal gyrus, orbitalis	-36	56	-5	3.85	0.0176					
	-30	26	-8	3.50	0.0552 (0.0008*)					
Middle-posterior temporal	-54	-37	-2	3.75	0.0448					
Anterior task-negative ROI						12	41	-17	3.89	0.0208
FLE non-lesional > CTR, Verb Generation fMRI										
Angular						36	-85	34	3.56	0.0538 (0.0004*)
FLE lesional < CTR, Verbal WM fMRI										
FEF/premotor ROI (BA 6-8)	-24	2	49	4.63	0.0050	27	11	52	5.74	0.0004
	-27	8	58	4.56	0.0060	51	23	31	3.57	0.073 (0.0006*)
	-12	8	61	4.21	0.0142	42	35	28	3.36	0.1188 (0.0006*)
Lateral prefrontal ROI (BA 9/46)	-39	8	31	4.88	0.0010	36	8	49	4.38	0.0052
	-39	29	28	3.95	0.0184	36	47	16	4.15	0.0106
	-27	32	28	3.90	0.0206	39	38	19	4.01	0.0154
Dorsal Parietal	-42	-49	52	5.78	0.0002	15	-70	61	6.24	0.0002
	-30	-58	55	5.68	0.0002	30	-79	37	5.36	0.0002
	-15	-67	52	5.15	0.0002	45	-46	46	4.69	0.0024
Posterior task-negative ROI (contiguous with dorsal parietal cluster^)						24	-76	34	4.52	0.0032
FLE lesional > CTR, Verbal WM fMRI										
Anterior task-negative ROI						0	44	-20	3.38	0.0954 (0.0006*)
Posterior task-negative ROI	-9	-52	34	3.22	0.1098 (0.0008*)					
FLE non-lesional < CTR, Verbal WM fMRI										
Dorsal Parietal						27	-76	37	3.56	0.1038 (0.0002*)
						39	-46	43	3.50	0.1202 (0.0004*)
FLE non-lesional > CTR, Verbal WM fMRI										
Anterior task-negative ROI	-6	50	-20	4.21	0.0078					
	-12	59	34	3.30	0.0904 (0.0010*)					
FLE lesional < CTR, Visual WM fMRI, 1- 0Back										
No significant differences										
FLE lesional > CTR, Visual WM fMRI, 1- 0Back										
FEF/premotor ROI (BA 6-8)						39	26	43	3.77	0.0804 (0.0006*)
						45	17	52	3.44	0.1780 (0.0008*)
Dorsal Parietal						27	-79	52	3.79	0.0848 (0.0008*)
FLE non-lesional < CTR, Visual WM fMRI, 1- 0 Back										
No significant differences										
FLE non-lesional > CTR, Visual WM fMRI, 1- 0 Back										
Dorsal Parietal						24	-76	55	3.6	0.1192 (0.0006*)
Anterior task-negative ROI	-15	62	13	3.54	0.0646 (0.0006*)					

FLE lesional < CTR, Visual WM fMRI, 2-1 Back										
FEF/premotor ROI (BA 6-8)	-39	-1	58	3.87	0.0310	33	11	58	4.45	0.0060
	-39	-1	49	3.61	0.0602 (0.0002*)					
	-15	-1	67	3.43	0.0936 (0.0006*)					
Lateral prefrontal ROI (BA 9/46)	-42	8	25	3.81	0.0262	39	5	28	3.77	0.0286
	-42	23	28	3.68	0.0364					
Dorsal Parietal	-15	-70	58	3.93	0.0414	18	-70	61	4.37	0.0116
	-21	-64	43	3.59	0.0938 (0.0010*)	54	-40	55	4.08	0.0262
						30	-61	61	4.04	0.0286
						30	-61	46	4.03	0.0296
FLE lesional > CTR, Visual WM fMRI, 2-1 Back										
No significant differences										
FLE non-lesional < CTR, Visual WM fMRI, 2-1 Back										
FEF/premotor ROI (BA 6-8)	-27	14	61	4.67	0.0028	36	8	55	4.86	0.0010
	-42	8	55	4.06	0.0198					
	-39	-1	46	3.81	0.0372					
Lateral prefrontal ROI (BA 9/46)	-36	17	25	4.3	0.0080	39	8	52	4.00	0.0174
	-45	11	31	4.16	0.0114	27	23	52	3.70=	0.0438
	-42	2	46	3.91	0.0220					
	-48	47	10	3.44	0.0844 (0.0010*)					
Dorsal Parietal	-51	-46	40	4.75	0.0036	12	-67	55	4.20	0.0178
	-12	-64	55	4.55	0.0054	30	-79	40	4	0.0348
						36	-52	37	3.9	0.0428
						42	-52	49	3.69	0.0768 (0.0010*)
						33	-64	52	3.67	0.0804 (0.0010*)
Posterior task-negative ROI (within task activation map^)						24	-58	16	3.96	0.0204
FLE non-lesional > CTR, Visual WM fMRI, 2-1 Back										
No significant differences										

Abbreviations: BA= Brodmann Area; FEF= frontal eye field; FLE= patients with frontal lobe epilepsy; FWE= family-wise error; MNI= Montreal Neurological Institute; ROI= region of interest. WM= working memory. Coordinates of fMRI group differences are provided in MNI space. The table reports statistics of permutation-based 2-tailed *t*-tests, using 10000 permutations and age and sex as covariates of no interest; *p*-values are voxel-wise FWE-corrected for multiple comparisons within prespecified ROIs (Fig. 1), unless otherwise stated. All *p*-values refer to 2-tailed statistical significance. ^ A small portion of the dorsal precuneus, formally belonging to the posterior task-negative ROI, displays task-related activation during WM. *P<0.001, uncorrected for multiple comparisons.

Supplementary Table 11. Language and Working Memory fMRI: comparison of lesional and non-lesional FLE patients

Group comparisons, MNI152 coordinates and test statistics

Region	**MNI coordinates (x y z)**			***T* score**	***P* value (FWE)**	**MNI coordinates (x y z)**			***T* score**	***P* value (FWE)**
	Left hemisphere					***Right hemisphere***				
FLE lesional < non-lesional, Verbal fluency fMRI										
No significant differences										
FLE lesional > non-lesional, Verbal fluency fMRI										
Inferior frontal gyrus, orbitalis	-39	50	-5	3.40	0.1184 (0.0008*)					
Middle-posterior temporal	-57	-34	1	4.36	0.0178	48	-40	1	3.93	0.0624 (0.0008*)
Posterior temporal						45	-43	22	3.45	0.1142 (0.0008*)
FLE lesional < non-lesional, Verb Generation fMRI										
No significant differences										
FLE lesional > non-lesional, Verb Generation fMRI										
Middle-anterior temporal	-54	-13	-20	3.75	0.0274					
FLE lesional < non-lesional, Verbal WM fMRI										
FEF/premotor ROI (BA 6-8)	-9	11	52	3.55	0.0868 (0.0010*)	24	-1	64	4.52	0.0076
	-15	2	67	3.55	0.0890 (0.0008*)	36	-1	61	4.29	0.0140
						6	-1	67	3.92	0.0350
Lateral prefrontal ROI (BA 9/46)						36	41	40	3.76	0.0464
						24	59	22	3.63	0.0618 (0.0002*)
Dorsal Parietal ROI						12	-73	61	3.95	0.0622 (0.0004*)
FLE lesional vs non-lesional, Verbal WM fMRI										
No significant differences										
FLE lesional vs non-lesional, Visual WM, 1-0 Back										
No significant differences										
FLE lesional vs non-lesional, Visual WM, 2-1 Back										
No significant differences										

Abbreviations: BA= Brodmann Area; CTR= controls; FCD= focal cortical dysplasia; FEF= frontal eye field; FLE= patients with frontal lobe epilepsy; FWE= family-wise error; MNI= Montreal Neurological Institute; ROI= region of interest. Coordinates of fMRI activation differences are provided in MNI space. The table reports statistics associated with permutation-based two-tailed *t*-tests, conducted with age and sex as covariates of no interest, and 10000 permutations; the associated *p*-values are voxel-wise FWE-corrected for multiple comparisons within pre-defined regions of interest (Fig. 1), unless otherwise stated. All the reported *p*-values refer to 2-tailed statistical significance. *$P<0.001$, uncorrected for multiple comparisons.

Supplementary Table 12. Language fMRI and cognitive test scores

MNI152 coordinates and test statistics for multiple regressions

Region	MNI coordinates (x y z)			*T* score	*P* value (FWE)	MNI coordinates (x y z)			*T* score	*P* value (FWE)
	Left hemisphere					*Right hemisphere*				
VF fMRI & letter fluency - positive										
Inferior frontal gyrus	-51	26	28	3.56	0.1080 (0.0008*)					
VF fMRI & letter fluency - negative										
Posterior task-negative ROI	-6	-61	52	3.99	0.0184	12	-61	52	3.64	0.0488
VF fMRI & naming - positive										
Middle-posterior temporal	-54	-37	1	3.79	0.0556 (0.0002*)					
VF fMRI & naming - negative										
Inferior frontal gyrus						48	20	31	3.63	0.0762 (0.0008*)
Angular	-51	-76	28	3.39	0.0812 (0.0010*)					
Posterior task-negative ROI	-9	-79	40	4.03	0.0140					
	-9	-61	49	3.63	0.0408					
VG fMRI & category fluency - positive										
Inferior frontal gyrus	-48	29	22	4.19	0.0108					
Inferior frontal gyrus, orbitalis	-30	32	-2	3.95	0.0174					
Middle-posterior temporal	-57	-55	1	3.68	0.0548 (0.0006*)					
VG fMRI & category fluency - negative										
No significant associations										
VG fMRI & naming - positive										
Inferior frontal gyrus	-51	20	16	4.44	0.0028					
	-42	8	25	4.03	0.0134					
Inferior frontal gyrus, orbitalis	-27	29	-5	3.81	0.0112					
	-42	29	-11	3.46	0.0318					
Anterior temporal	-48	2	-26	3.46	0.0688 (0.0006*)					
Middle-posterior temporal	-48	-43	-11	3.95	0.0230					
	-54	-40	1	3.67	0.0484					
Posterior temporal	-45	-52	19	3.78	0.0278					
	-45	-58	-2	3.57	0.0498					
VG fMRI & naming - negative										
Angular						42	-79	34	4.36	0.0040
						33	-88	34	3.96	0.0124
						54	-67	34	3.66	0.0342

Abbreviations: CTR= controls; FWE= family-wise error; MNI= Montreal Neurological Institute; ROI= region of interest; VF=verbal fluency; VG= verb generation. Permutation-based multiple regression analyses were conducted with 10000 permutations, using age, sex, and group allocation as covariates of no interest; the associated *p*-values are voxel-wise FWE-corrected for multiple comparisons within predefined regions of interest (Fig. 1), unless otherwise stated. All the reported *p*-values refer to 2-tailed statistical significance. *$P<0.001$, uncorrected for multiple comparisons. Associations between left inferior frontal activation during verbal fluency fMRI and naming scores were evident at p<0.005 uncorrected only (x=-48, y=8, z=22, t=3.36, p_{FWE}=0.135, p_{unc}=0.0016).

Supplementary Table 13. Language fMRI and clinical characteristics, FLE

MNI152 coordinates and test statistics for multiple regressions

Region	**MNI coordinates (x y z)**			***T* score**	***P* value (FWE)**	**MNI coordinates (x y z)**			***T* score**	***P* value (FWE)**
	Left hemisphere					***Right hemisphere***				
Verbal Fluency fMRI & age at onset – FLE, positive/negative										
No significant associations										
Verbal Fluency fMRI & disease duration – FLE, positive										
*Middle frontal gyrus^*	-39	35	46	4.93	0.0416					
Posterior task-negative ROI	-3	73	46	3.74	0.0556 (0.0006*)					
Verbal Fluency fMRI & seizure frequency – FLE, positive/negative										
No significant associations										
Verbal Fluency fMRI & FBTCS – FLE, positive/negative										
No significant associations										
Verbal Fluency fMRI & time since last seizure – FLE, negative										
Anterior temporal						48	5	-26	5.12	0.0750 (0.0008*)
Verb Generation fMRI & age at onset – FLE, positive/negative										
No significant associations										
Verb Generation fMRI & disease duration – FLE, positive										
Posterior temporal gyrus						57	-58	34	4.29	0.0098
Angular gyrus	-51	-70	37	3.72	0.0462					
Verb Generation fMRI & seizure frequency – FLE, positive/negative										
No significant associations										
Verb Generation fMRI & FBTCS – FLE, positive/negative										
No significant associations										
Verb Generation fMRI & time since last seizure – FLE, negative										
Anterior temporal	-54	-7	-23	4.31	0.0092					
Middle-anterior temporal	-54	-10	-23	4.65	0.0044					

Abbreviations: FBTCS= focal-to-bilateral tonic-clonic seizures; FLE= patients with frontal lobe epilepsy; FWE= family-wise error; MNI= Montreal Neurological Institute; ROI= region of interest. Permutation-based multiple regression analyses were conducted with 10000 permutations, using the following covariates of no interest: sex and side of epilepsy (for age at onset/disease duration models); age, sex, and side of epilepsy (for seizure frequency, FBTCS and time since last seizure models). The associated *p*-values are voxel-wise FWE-corrected for multiple comparisons within predefined regions of interest (Fig. 1), unless otherwise stated. All the reported *p*-values refer to 2-tailed statistical significance. ^Area outside of prespecified ROIs, with voxel-based statistic surviving 2-tailed $p_{FWE}<0.05$, corrected across the whole brain. *$P<0.001$, uncorrected for multiple comparisons.

Supplementary Table 14. Language fMRI and clinical characteristics, TLE

MNI152 coordinates and test statistics for multiple regressions

Region	**MNI coordinates (x y z)**			***T* score**	***P* value (FWE)**	**MNI coordina tes (x y z)**			***T* score**	***P* value (FWE)**
	Left hemisphere					***Right hemisphere***				
Verbal Fluency fMRI & age at onset – TLE, positive										
Inferior frontal gyrus, orbitalis	-33	29	17	3.82	0.0488					
Verbal Fluency fMRI & duration – TLE, positive/negative										
No significant associations										
Verbal Fluency fMRI & seizure frequency – TLE, positive/negative										
No significant associations										
Verbal Fluency fMRI & FBTCS – TLE, negative										
Anterior temporal	-51	2	-20	4.44	0.0072	36	-1	-20	4.34	0.0088
	-39	-7	-20	4.21	0.0146	54	8	-32	3.85	0.0368
	-48	-7	-29	3.82	0.0406	42	-1	-38	3.44	0.1040 (0.0006*)
Middle-anterior temporal	-39	-13	-14	5.11	0.0010	54	-16	-17	4.57	0.0054
	-42	-19	-2	4.21	0.0154	39	-10	-11	3.64	0.0572 (0.0008*)
	-42	-13	-26	3.86	0.0370					
Middle-posterior temporal						42	-31	-17	4.34	0.0174
						39	-28	-5	3.53	0.1314 (0.0008*)
Posterior temporal						51	-67	22	4.37	0.0106
						54	-61	-8	4.25	0.0154
						39	-67	19	4.20	0.0174
Angular gyrus						48	-73	25	5.07	0.0022
						39	-70	22	4.60	0.0066
						36	-82	28	3.98	0.0308
Anterior task-negative ROI	-3	62	-5	4.13	0.0214	9	65	-5	3.76	0.0602 (0.0006*)
Posterior task-negative ROI	-9	-43	43	3.73	0.0576 (0.0008*)	12	-40	4	4.19	0.0164
						9	-46	43	4.12	0.0192
						21	-73	40	3.81	0.0462
Insula/parietal operculum						36	-34	-20	6.45	0.0002
Entorhinal cortex	-24	-7	-32	5.62	0.0046					
Inferior temporal gyrus						33	-1	-41	5.21	0.0174
Verbal Fluency fMRI & time since last seizure – TLE, positive/ negative										
No significant associations										
Verb Generation fMRI & age at onset – TLE, positive/negative										
No significant associations										
Verb Generation fMRI & duration – TLE, positive/negative										
No significant associations										
Verb Generation fMRI & seizure frequency – TLE, positive/negative										
No significant associations										
Verb Generation fMRI & FBTCS – TLE, positive										
Middle-anterior temporal	-63	-25	-8	3.62	0.0592 (0.0008*)					

Middle-posterior temporal	-66	-40	1	3.78	0.0794 (0.0004*)	42	31	-2	4.26	0.0242
Verb Generation fMRI & time since last seizure – TLE, positive/ negative										
No significant associations										

Abbreviations: FBTCS= focal-to-bilateral tonic-clonic seizures; FWE= family-wise error; MNI= Montreal Neurological Institute; ROI= region of interest; TLE= patients with frontal lobe epilepsy. Permutation-based multiple regression analyses were conducted with 10000 permutations, using the following covariates of no interest: sex and side of epilepsy (for age at onset/disease duration models); age, sex, and side of epilepsy (for seizure frequency, FBTCS and time since last seizure models). The associated *p*-values are voxel-wise FWE-corrected for multiple comparisons within predefined regions of interest (Fig. 1), unless otherwise stated. All the reported *p*-values refer to 2-tailed statistical significance. *P<0.001, uncorrected for multiple comparisons.

Supplementary Table 15. Working Memory fMRI and cognitive test scores

MNI152 coordinates and test statistics for multiple regressions

Region	**MNI coordinates (x y z)**			***T* score**	***P* value (FWE)**	**MNI coordinates (x y z)**			***T*- score**	***P* value (FWE)**
	Left hemisphere					***Right hemisphere***				
Verbal WM fMRI &digit span - positive										
FEF/premotor ROI (BA 6-8)	-33	5	64	5.25	0.0002	27	14	64	5.92	0.0002
	-24	11	55	4.58	0.0040	42	38	34	4.83	0.0014
	-42	26	34	4.30	0.0104	48	29	37	4.51	0.0046
	-3	26	55	3.820	0.0426	24	32	40	3.84	0.0396
Lateral prefrontal ROI (BA 9/46)	-42	11	34	4.70	0.0026	36	11	46	5.18	0.0002
	-42	29	28	4.43	0.0062	39	5	34	4.64	0.0032
	-51	23	31	4.28	0.0108	39	41	34	4.97	0.0004
	-33	56	13	4.22	0.0124	27	23	52	4.66	0.0026
	-45	5	52	3.97	0.0256	27	32	40	4.10	0.0168
						33	56	1	4.96	0.0004
Dorsal Parietal ROI	-30	-64	52	5.47	0.0004	33	-67	49	6.35	0.0002
	-48	-49	58	3.95	0.0326	27	-82	46	5.80	0.0002
Anterior task-negative ROI (within activation map) #	15	38	16	3.66	0.0350					
Posterior task-negative ROI (within activation map) #	-6	-76	46	3.55	0.0482	9	-73	43	4.31	0.0050
						24	-76	34	3.77	0.0242
						3	-55	49	3.64	0.0384
*Dorsal parietal (outside prespecified ROI, within activation map)^*	-6	-70	52	4.88	0.0134^	15	-67	64	5.16	0.0042^
	-15	-82	49	4.70	0.0254^	9	-64	52	4.81	0.0178^
*Cerebellum^*	-6	-46	-20	5.22	0.0034^	9	-40	-35	5.03	0.0064^
						6	-46	-20	5.01	0.0076^
*Frontopolar cortex^*						21	62	-8	4.58	0.0346^
Verbal WM fMRI &digit span - negative										
Posterior task-negative ROI						3	-55	25	3.48	0.0544 (0.0008*)
Verbal WM fMRI & verbal 2Back - positive										
FEF/premotor ROI (BA 6-8)	-33	26	31	3.73	0.0406					
Lateral prefrontal ROI (BA 9/46)	-39	5	37	4.72	0.0010	36	41	13	4.18	0.0066
	-33	5	31	4.59	0.0014	36	11	40	3.62	0.0392
	-33	17	25	4.35	0.0030					
Dorsal Parietal ROI	-64	52	-64	3.83	0.0434	36	-49	37	4.19	0.0142
						36	-58	40	4.15	0.0156
						36	-43	37	4.06	0.0212
						42	-46	40	3.82	0.0458
*Dorsal parietal-- outside ROI boundaries^*						36	-46	34	4.61	0.0336^

*Inferior frontal gyrus^*	-33	14	22	4.80	0.0204^					
Verbal WM fMRI & verbal 2Back - negative										
No suprathreshold associations										
2-1 Back visual WM & visual 2Back - positive										
FEF/premotor ROI (BA 6-8)	-42	26	34	5.20	0.0002	33	14	58	5.90	0.0002
	-27	8	64	4.89	0.0004	24	35	43	4.28	0.0048
	-12	14	61	4.05	0.0098	0	26	52	4.90	0.0004
	-15	-7	67	3.56	0.0458	42	35	28	4.56	0.0010
						48	23	31	4.12	0.0080
Lateral prefrontal ROI (BA 9/46)	-42	29	31	5.42	0.0004	33	56	4	5.40	0.0004
	-39	20	28	4.98	0.0008	30	20	52	5.08	0.0008
	-48	8	46	4.02	0.0138	42	41	25	4.98	0.0008
	-39	50	16	3.86	0.0208	39	11	52	4.56	0.0016
Dorsal Parietal ROI						3	-64	49	7.71	0.0002
						27	-73	37	6.12	0.0002
						36	-64	46	5.89	0.0002
*Anterior task-negative ROI (within activation map)**	-6	29	55	4.12	0.0084	9	32	55	3.94	0.0150
						6	41	46	3.90	0.0170
						12	35	46	3.60	0.0364
						18	38	40	3.51	0.0492
Posterior task-negative ROI (within activation map)#	-12	-67	43	4.63	0.0018	3	-61	46	7.22	0.0002
	-6	-76	46	3.89	0.0214	6	-76	46	5.37	0.0004
						24	-76	34	4.93	0.0006
*Inferior frontal gyrus^*	-36	20	-8	6.1	0.0002	33	26	-5	6.72	0.0002
*Pre-supplementary motor area^*						6	29	52	5.05	0.0060
						9	38	40	4.39	0.0492
*Superior occipital gyrus^*	-30	-82	31	4.47	0.0380	6	-7	7	4.43	0.0442
*Thalamus^*	-15	-4	-8	4.94	0.0084					
	-12	-4	7	4.58	0.0284					
*Subthalamus/midbrain^*	-3	-16	-23	5.41	0.0016					
2-1 Back visual WM & visual 2 Back - negative										
No suprathreshold associations										

Abbreviations: BA= Brodmann Area; CTR= controls; FEF= frontal eye field; FWE= family-wise error; MNI= Montreal Neurological Institute; ROI= region of interest. Coordinates of voxels showing significant associations between fMRI activity and cognitive performance are provided in MNI space. Permutation-based multiple regression analyses were conducted with 10000 permutations, and age, sex, and group allocation as covariates of no interest; the associated *p*-values are voxel-wise FWE-corrected for multiple comparisons within predefined regions of interest (Fig. 1), unless otherwise stated. All the reported *p*-values refer to 2-tailed statistical significance. ^area outside of prespecified ROIs, surviving 2-tailed p_{FWE} <0.05, corrected voxel-wise across the whole brain. *P<0.001, uncorrected for multiple comparisons; #Small portions of the superior frontal gyrus/dorsal precuneus, formally belonging to the anterior/ posterior task-negative ROIs, display task-related activation during WM.

Supplementary Table 16. Working Memory fMRI and clinical characteristics, FLE

MNI152 coordinates and test statistics for multiple regressions

Region	**MNI coordinates (x y z)**			***T* score**	***P* value (FWE)**	**MNI coordinates (x y z)**			***T* score**	***P* value (FWE)**
	Left hemisphere					***Right hemisphere***				
Verbal WM fMRI & age at onset – FLE, positive										
Dorsal Parietal ROI	-33	-76	46	4.10	0.1314 (0.0004*)	48	-64	43	3.87	0.1826 (0.0002*)
						33	-88	34	3.41	0.3676 (0.0002*)

Lateral prefrontal ROI (BA 9/46)	-48	8	28	3.91	0.0774 (0.0002*)					
Verbal WM fMRI & duration – FLE, negative										
Dorsal Parietal ROI	-36	-49	37	4.55	0.0138	36	-43	37	4.22	0.0314
	-42	-55	31	4.29	0.0274					
FEF/premotor ROI (BA 6-8)	-39	14	46	3.67	0.0782 (0.0004*)					
Lateral prefrontal ROI (BA 9/46)	-45	14	49	3.72	0.0542 (0.0002*)					
Verbal WM fMRI & seizure frequency – FLE, positive/ negative										
No significant associations										
Verbal WM fMRI & FBTCS – FLE, negative										
Lateral prefrontal ROI (BA 9/46)						33	50	34	3.81	0.0494
Verbal WM fMRI & time since last seizure – FLE, negative										
No significant associations										
Visual WM fMRI (1-0 Back) & age at onset– FLE, positive/negative										
No significant associations										
Visual WM fMRI (1-0 Back) & duration– FLE, negative										
FEF/premotor ROI (BA 6-8)						30	17	49	3.72	0.0822 (0.0006*)
Visual WM fMRI (1-0) & seizure frequency– FLE, positive/negative										
No significant associations										
Visual WM fMRI (1-0 Back) & FBTCS– FLE, positive/negative										
No significant associations										
Visual WM fMRI (1-0 Back) & time since last seizure– FLE, negatives										
Dorsal Parietal ROI	-60	-52	40	3.78	0.1120 (0.0002*)					
Visual WM fMRI (2-1 Back)										
No significant associations with any clinical variable										

Abbreviations: FBTCS= focal-to-bilateral tonic-clonic seizures; FLE= patients with frontal lobe epilepsy; FWE= family-wise error; MNI= Montreal Neurological Institute; ROI= region of interest; WM= working memory. Permutation-based multiple regression analyses were conducted with 10000 permutations, using the following covariates of no interest: sex and side of epilepsy (for age at onset/disease duration models); age, sex, and side of epilepsy (for seizure frequency, FBTCS and time since last seizure models). The associated *p*-values are voxel-wise FWE-corrected for multiple comparisons within predefined regions of interest (Fig. 1), unless otherwise stated. All the reported *p*-values refer to 2-tailed statistical significance. *$P<0.001$, uncorrected for multiple comparisons.

<u>Supplementary Table 17. Working memory fMRI and clinical characteristics, TLE</u>

MNI152 coordinates and test statistics for multiple regressions

Region	**MNI coordinates (x y z)**			***T* score**	***P* value (FWE)**	**MNI coordinates (x y z)**			***T* score**	***P* value (FWE)**
	Left hemisphere					***Right hemisphere***				
Verbal WM fMRI & age at onset – TLE, positive/negative										
No significant associations										
Verbal WM fMRI & duration – TLE, positive/negative										
No significant associations										
Verbal WM fMRI & seizure frequency – TLE, positive/ negative										
No significant associations										
Verbal WM fMRI & FBTCS – TLE, negative										
FEF/premotor ROI (BA 6-8)						12	14	37	3.69	0.1024 (0.0004*)
Lateral prefrontal ROI (BA 9/46)	-36	2	34	3.86	0.0586 (0.0004*)	30	29	31	4.65	0.0072
						42	-1	40	3.88	0.0570 (0.0004*)
						36	44	4	3.64	0.0992 (0.0004*)
Dorsal Parietal ROI	-57	-31	49	3.7	0.1030 (0.0010*)					
	-24	-82	25	3.57	0.1374 (0.001*)					
Anterior task-negative ROI	-18	56	37	3.19	0.1438 (0.0006*)					
*Precentral gyrus^*						33	2	35	4.88	0.0260
						36	-7	37	4.82	0.0304
Verbal WM fMRI & time since last seizure – TLE, negative										
Lateral prefrontal ROI (BA 9/46)						33	26	46	3.1	0.1878 (0.0006*)
Visual WM fMRI (1-0) & age at onset– TLE, positive/negative										
No significant associations										
Visual WM fMRI (1-0) & duration– TLE, negative										
Posterior task-negative ROI	-6	-43	7	3.93	0.0224	6	-46	7	4.03	0.0168
	-3	-46	40	3.74	0.0362	0	-61	16	3.62	0.0470
Visual WM fMRI (1-0) & seizure frequency– TLE, positive/negative										
No significant associations										
Visual WM fMRI (1-0) & FBTCS– TLE, positive/negative										
No significant associations										
Visual WM fMRI (1-0) & time since last seizure– TLE, negatives										
Dorsal Parietal ROI										
Visual WM fMRI (2-1)										
<u>No significant associations with any clinical variable</u>										

Abbreviations: FBTCS= focal-to-bilateral tonic-clonic seizures; FWE= family-wise error; MNI= Montreal Neurological Institute; ROI= region of interest; TLE= patients with temporal lobe epilepsy. Permutation-based multiple

regression analyses were conducted with 10000 permutations, using the following covariates of no interest: sex and side of epilepsy (for age at onset/disease duration models); age, sex, and side of epilepsy (for seizure frequency, FBTCS and time since last seizure models). The associated *p*-values are voxel-wise FWE-corrected for multiple comparisons within predefined regions of interest (Fig. 1). All the reported *p*-values refer to 2-tailed statistical significance. ^Area outside of prespecified ROIs, with voxel-based statistic surviving 2-tailed $p_{FWE}<0.05$, corrected across the whole brain. *$P<0.001$, uncorrected for multiple comparisons.

SUPPLEMENTARY REFERENCES

e1. Caciagli L, Wandschneider B, Centeno M, Vollmar C, Vos SB, Trimmel K, *et al.* Motor hyperactivation during cognitive tasks: An endophenotype of juvenile myoclonic epilepsy. Epilepsia 2020; 61(7): 1438-52.

e2. Dale AM, Fischl B, Sereno MI. Cortical surface-based analysis. I. Segmentation and surface reconstruction. Neuroimage 1999; 9(2): 179-94.

e3. Fischl B, Sereno MI, Dale AM. Cortical surface-based analysis. II: Inflation, flattening, and a surface-based coordinate system. Neuroimage 1999; 9(2): 195-207.

e4. Greve DN, Fischl B. Accurate and robust brain image alignment using boundary-based registration. Neuroimage 2009; 48(1): 63-72.

e5. Fedorenko E, Hsieh PJ, Nieto-Castanon A, Whitfield-Gabrieli S, Kanwisher N. New method for fMRI investigations of language: defining ROIs functionally in individual subjects. J Neurophysiol 2010; 104(2): 1177-94.

e6. Chai LR, Mattar MG, Blank IA, Fedorenko E, Bassett DS. Functional Network Dynamics of the Language System. Cereb Cortex 2016; 26(11): 4148-59.

e7. Fan L, Li H, Zhuo J, Zhang Y, Wang J, Chen L, *et al.* The Human Brainnetome Atlas: A New Brain Atlas Based on Connectional Architecture. Cereb Cortex 2016; 26(8): 3508-26.

e8. Alves PN, Foulon C, Karolis V, Bzdok D, Margulies DS, Volle E, *et al.* An improved neuroanatomical model of the default-mode network reconciles previous neuroimaging and neuropathological findings. Commun Biol 2019; 2: 370.

e9. Wilke M, Lidzba K. LI-tool: a new toolbox to assess lateralization in functional MR-data. J Neurosci Methods 2007; 163(1): 128-36.
