# Supplementary material for: Disorganization of language and working memory systems in frontal versus temporal lobe epilepsy
Source: Brain. 2022 May 2;146(3):935–53. doi: 10.1093/brain/awac150 (PMC9976988; doi:10.1093/brain/awac150)
Supplement: awac150_Supplementary_Data [file awac150_supplementary_data.zip › brain-2021-00401-File011.pdf]

Citation Diversity Statement

Recent work in several fields of science has identified a bias in citation practices such that papers from women and other minority scholars are under-cited relative to the number of such papers in the field.[1-9] Here we sought to proactively consider choosing references that reflect the diversity of the field in thought, form of contribution, gender, race, ethnicity and other factors. First, we obtained the predicted gender of the first and last author of each reference by using databases that store the probability of a first name being carried by a woman[5,10]. By this measure (and excluding self-citations to the first and last authors of our current paper), our references contain 11.11% woman(first)/woman(last), 14.14% man/woman, 27.27% woman/man, and 47.47% man/man. This method is limited in that a) names, pronouns and social media profiles used to construct the databases may not, in every case, be indicative of gender identity, and b) it cannot account for intersex, non-binary, or transgender people. Second, we obtained predicted racial/ethnic category of the first and last author of each reference by databases that store the probability of a first and last name being carried by an author of color.[11,12] By this measure (and excluding self-citations), our references contain 8.38% author of color (first)/author of color(last), 13.06% white author/author of color, 18.66% author of color/white author, and 59.90% white author/white author. This method is limited in that a) names, Census entries, and Wikipedia profiles used to make the predictions may not be indicative of racial/ethnic identity, and b) it cannot account for Indigenous and mixed-race authors, or those who may face differential biases due to the ambiguous racialization or ethnicization of their names. We look forward to future work that could help us to better understand how to support equitable practices in science.

References for the Citation Diversity Statement

1. Mitchell SM, Lange S, Brus H. Gendered citation patterns in international relations journals. *International Studies Perspectives*. 2013;14(4):485-492.
2. Maliniak D, Powers R, Walter BF. The gender citation gap in international relations. *International Organization*. 2013;67(4):889-922.
3. Caplar N, Tacchella S, Birrer S. Quantitative evaluation of gender bias in astronomical publications from citation counts. *Nature Astronomy*. 2017;1(6):1-5.
4. Dion ML, Sumner JL, Mitchell SM. Gendered citation patterns across political science and social science methodology fields. *Political Analysis*. 2018;26(3):312-327.

5. Dworkin JD, Linn KA, Teich EG, Zurn P, Shinohara RT, Bassett DS. The extent and drivers of gender imbalance in neuroscience reference lists. *Nat Neurosci*. Aug 2020;23(8):918-926. doi:10.1038/s41593-020-0658-y
6. Bertolero MA, Dworkin JD, David SU, *et al*. Racial and ethnic imbalance in neuroscience reference lists and intersections with gender. *BioRxiv*. 2020;
7. Wang X, Dworkin JD, Zhou D, *et al*. Gendered citation practices in the field of communication. *Annals of the International Communication Association*. 2021;45(2):134-153.
8. Chatterjee P, Werner RM. Gender disparity in citations in high-impact journal articles. *JAMA Network Open*. 2021;4(7):e2114509-e2114509.
9. Fulvio JM, Akinnola I, Postle BR. Gender (im) balance in citation practices in cognitive neuroscience. *Journal of Cognitive Neuroscience*. 2021;33(1):3-7.
10. Zhou D, Cornblath EJ, Stiso J, *et al*. Gender Diversity Statement and Code Note-Book v1. 0. Zenodo; 2020.
11. Ambekar A, Ward C, Mohammed J, Male S, Skiena S. Name-ethnicity classification from open sources. 49-58.
12. Sood G, Laohaprapanon S. Predicting race and ethnicity from the sequence of characters in a name. *arXiv preprint arXiv:180502109*. 2018;
